# A Rfa1-MN–based system reveals new factors involved in the rescue of broken replication forks

**DOI:** 10.1371/journal.pgen.1011405

**Published:** 2025-04-01

**Authors:** Ana Amiama-Roig, Marta Barrientos-Moreno, Esther Cruz-Zambrano, Luz M. López-Ruiz, Román González-Prieto, Gabriel Ríos-Orelogio, Félix Prado

**Affiliations:** Centro Andaluz de Biología Molecular y Medicina Regenerativa (CABIMER), Consejo Superior de Investigaciones Científicas, Universidad de Sevilla, Universidad Pablo de Olavide, Seville, Spain; National Institute of Environmental Health Sciences, UNITED STATES OF AMERICA

## Abstract

The integrity of the replication forks is essential for an accurate and timely completion of genome duplication. However, little is known about how cells deal with broken replication forks. We have generated in yeast a system based on a chimera of the largest subunit of the ssDNA binding complex RPA fused to the micrococcal nuclease (Rfa1-MN) to induce double-strand breaks (DSBs) at replication forks and searched for mutants affected in their repair. Our results show that the core homologous recombination (HR) proteins involved in the formation of the ssDNA/Rad51 filament are essential for the repair of DSBs at forks, whereas non-homologous end joining plays no role. Apart from the endonucleases Mus81 and Yen1, the repair process employs fork-associated HR factors, break-induced replication (BIR)-associated factors and replisome components involved in sister chromatid cohesion and fork stability, pointing to replication fork restart by BIR followed by fork restoration. Notably, we also found factors controlling the length of G1, suggesting that a minimal number of active origins facilitates the repair by converging forks. Our study has also revealed a requirement for checkpoint functions, including the synthesis of Dun1-mediated dNTPs. Finally, our screening revealed minimal impact from the loss of chromatin factors, suggesting that the partially disassembled nucleosome structure at the replication fork facilitates the accessibility of the repair machinery. In conclusion, this study provides an overview of the factors and mechanisms that cooperate to repair broken forks.

## Author summary

The cellular mechanisms that respond to broken replication forks remain poorly understood, despite the fact that genomic instability arising during DNA replication is a hallmark of early cancer progression. A major limitation in addressing this gap is the absence of robust systems to systematically screen for the genetic factors involved. Recently, genetic systems have been developed to induce replication fork breakage via a DNA nick—an intermediate step that is physiologically relevant in DNA repair and topological regulation. However, the cellular response to direct double-strand breaks (DSBs) at replication forks, such as those resulting from fork collapse or unscheduled nuclease activity, remains largely unexplored. In this study, we engineered a chimeric protein, Rfa1-MN, which fuses the largest subunit of the single-stranded DNA-binding complex RPA with micrococcal nuclease (MN). This chimera preferentially generates DSBs at replication forks, enabling us to screen for mutants impaired in fork repair. Our screening identified novel factors that highlight the significance of error-prone break-induced replication (BIR) restart, fork restoration from BIR-intermediates and rescue by converging forks. Specifically, recombination factors associated with replication forks, replisome components critical for fork stability, and regulators of the G1 phase—controlling replication origin number— are potential players to regulate the efficiency of these pathways and the impact of broken fork repair on genome integrity.

## Introduction

DNA replication duplicates the genome during the S phase of the cell cycle. This essential process requires the coordinated firing of multiple replicons with bidirectional replisomes copying large genomic regions. The integrity of the replication fork is threat by its intrinsically fragile molecular nature (a dynamic nucleosome-free structure with DNA ends and single-stranded DNA; ssDNA) and the presence of multiple factors that hamper its advance (DNA adducts, abasic sites, ribonucleoside monophosphates (rNMPs), specific DNA structures like G-quadruplexes or R-loops, other processes like transcription and unbalanced supplies of deoxynucleoside triphosphates (dNTPs) or histones) [1]. Dealing with these situations is critical not only for a timely completion of genome duplication but also to prevent genetic instability. Accordingly, cells are endowed with different mechanisms that protect and repair stalled forks [2–5]. Much less is known, though, about the mechanisms that deal with double-strand breaks (DSBs) at forks despite DSBs at linear molecules are one of the most deleterious DNA lesions and their repair has been extensively studied from yeast to human [6–9]. The reason is that most of those studies took advantage of DNA sequence-specific endonucleases that allowed to follow the repair process; in contrast, DSBs at forks are spread along the genome at different positions in each cell, making difficult their analysis.

DSBs at forks have been proposed to be repaired by break-induced replication (BIR), a homologous recombination (HR) process in which the homology is restricted to one end; upon invasion of a homologous template, DNA synthesis can proceed for large genomic regions [10]. BIR, which has been extensively characterized in yeast, does not assemble a canonical fork; instead, it proceeds through a conservative DNA synthesis mechanism that is associated with a migrating bubble-like replication fork in which the Polδ subunit Pol32 becomes essential [11]. This structure is highly mutagenic and unstable, leading to multiple template-switching events and genome rearrangements that resemble those occurring in cancer genomes [12,13]. In terms of repair proteins, the most relevant difference with other DSB-induced HR events is that it can occur – though more inefficiently – in the absence of Rad51 [14]. In accordance with BIR acting upon DSBs at forks, mutants defective in replication-coupled nucleosome assembly accumulate broken forks that are rescued by a Rad52-dependent, Rad51-independent HR mechanism [15]. However, it is unknown if this requirement is specific of broken forks under conditions of altered chromatin.

A major handicap to associate BIR with broken forks is that the systems to study BIR follow the repair of a DNA sequence-specific endonuclease-induced DSB with a homologous sequence located on an ectopic region. As an alternative, genetic systems have been used in which an induced nick is converted into a DSB when encountered by a replication fork [16–23]. DNA nicks are physiologically relevant because they are common intermediates of DNA repair and topological processes that are targeted in therapeutic treatments in cancer. A nick at the leading template causes a single-ended DSB (seDSB) that is rescued by an error-prone BIR-like process [17–20]; however, BIR-associated synthesis is limited by two compensatory mechanisms: cleavage by the Mus81 endonuclease to convert the D-loop into a canonical fork and arrival of a converging fork [17]. Actually, the arrival of a converging fork before BIR might explain the detection of double-ended DSB (deDSBs) at some nicks at the leading template [20–22]. A nick at the lagging strand also leads to a seDSB using nicked plasmids in *Xenopus* egg extracts [19], and accordingly, DNA nicks in both leading and lagging strand templates can trigger BIR [22]. However, nickase-induced nicks at the lagging template can be bypassed by the replisome in yeast and mammalian cells leaving a deDSB behind the fork [20–23]. This bypass, though, depends on the nickase and structure of the DNA nick [19,20,22], indicating that the fork can respond to the nick and/or the nickase without collapsing.

Replication forks can also collapse and break directly under genetic or environmental conditions that cause replicative stress as those occurring during tumour development [24]. An *in vitro* approach to this type of DNA lesions treated *Xenopus* egg extracts with ssDNA-specific endonucleases such as S1 or mung bean nuclease, which cut preferentially at the fork where ssDNA accumulates under unperturbed conditions. This study showed that the replisome is partially dismantled after fork breakage but fully re-established by a HR process that requires the nuclease activity of Mre11, Rad51 and the initial DNA synthesis activity of Polε [25].

In this study, we have developed an *in vivo* system that induces DSBs preferentially at the replication forks and searched for mutants defective in their repair. This genetic analysis demonstrated that the HR factors involved in the formation of the ssDNA/Rad51 nucleofilament are essential for the repair of DSBs at forks. In contrast to canonical DSBs at linear molecules, the repair of DSBs at forks is facilitated by fork-associated HR factors, BIR-associated factors, replisome components and a timely G1 phase. These results suggest that cells deal with seDSBs at broken forks by two mechanisms: BIR followed by fork restoration and rescue by converging replication forks, and reveal new player controlling their efficiency.

## Results

### The chimera Rfa1-MN provides a genetic system to study the repair of DSBs at replication forks

**Fig 1:**
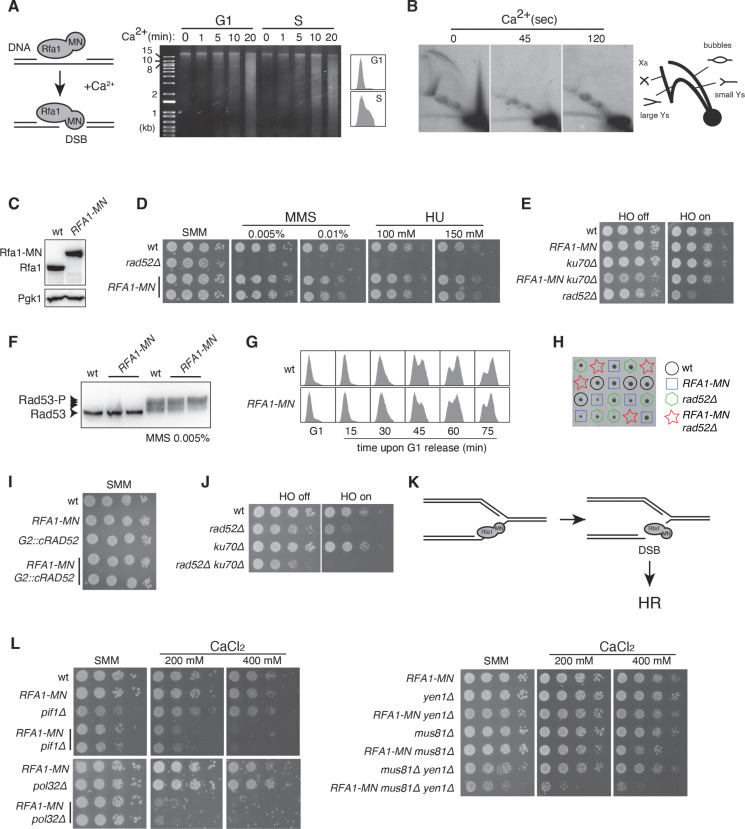
The chimera Rfa1-MN provides a genetic system to study the repair of DSBs at replication forks. **(A)** ChEC analysis of *RFA1-MN* cells arrested in G1 with α-factor and released into S phase for 30 minutes. Total DNA from cells permeabilized and treated with 2 mM CaCl_2_ for different times is shown, as well as the FACS profiles. A scheme with the rational of the ChEC approach is shown on the left. **(B)** 2D/ChEC analysis of replication intermediates of *RFA1-MN* cells synchronised in G1 with α-factor and released into S phase for 30 minutes. Total DNA from cells permeabilized and treated with Ca2+ for different times was digested with specific restriction enzymes and analysed by 2D electrophoresis. A schematic representation of the migration pattern of replication intermediates is shown on the right. **(C)** Rfa1 expression in wild-type and *RFA1-MN* cells from exponentially growing cultures as determined by western blot analysis. **(D)** MMS and HU sensitivity of *RFA1-MN* cells. Wild-type and *rad52∆* cells were included as controls. **(E)** DSB sensitivity of *RFA1-MN* cells transformed with pGAL-HO and grown in glucose (*GAL1p* repression) and galactose-containing medium (*GAL1p* activation). An HO-induced DSB at the *MAT* locus can be repaired by NHEJ or, preferentially, by HR with the *HMR* or *HML* donor. The analysis was performed in wild-type and *ku70∆* background (defective in NHEJ). **(F)** Rad53 activation in wild-type and *RFA1-MN* cells as determined by western blot analysis of exponentially growing cultures either in the absence or presence of 0.005% MMS for 1 hour. **(G)** Cell cycle progression of wild-type and *RFA1-MN* cells synchronised in G1 with α-factor and released into S phase for different times as determined by FACS analysis. **(H)** *RFA1-MN rad52∆* lethality as determined by tetrad analysis. (**I)** Effect of restricting Rad52 expression to G2/M in wild type (*G2::cRAD52*) and *RFA1-MN* cells (*RFA1-MN G2::cRAD52*). **(J)** HO-induced DSB repair in cells defective in HR (*rad52∆*) and/or NHEJ (*ku70∆*). Cells were transformed with pGAL-HO and grown in glucose (*GAL1p* repression) and galactose-containing medium (*GAL1p* activation). **(K)** Proposed model for the essential role of HR in Rfa1-MN expressing cells. **(L)** Effect of the *pif1∆*, *pol32∆*, *yen1∆*, *mus81∆* and *mus81∆ yen1∆* mutations in the growth of *RFA1-MN* cells in the absence and presence of different CaCl_2_ concentrations. At high concentration, CaCl_2_ can form crystals that did not affect the reproducibility of the assay. (D-E, I-J, L) Cell growth was determined by spotting 10-fold serial dilutions of the same number of mid-log growing cells onto the indicated mediums. All the analyses were repeated at least twice with similar results.

Chromatin endogenous cleavage (ChEC) provides a method to detect protein chromatin binding [26]. This assay is based in the expression of a chimera of the protein of interest with the micrococcal nuclease (MN), whose nucleolytic activity is activated with Ca^2+^ ions. If the protein is bound to DNA, activation of the MN domain will induce a detectable cut (Fig 1A, left). Since the intracellular levels of Ca^2+^ ions are low for MN activation, this assay requires cells to be permeabilized with digitonin followed by addition of CaCl_2_ (S1A Fig). This assay has also been used to follow the binding of repair proteins to non-DSBs DNA lesions, as the ssDNA fragments generated by the encounter of replication forks with methyl methanesulfonate (MMS)-induced DNA adducts [27–33].

An example of this approach is the chimera Rfa1-MN, which contains the largest subunit of the ssDNA binding complex RPA. RPA is an essential complex involved in replication fork stability, DNA repair and checkpoint activation [34,35]. After treating permeabilized cells with CaCl_2_ for different times, total DNA was extracted and run into an agarose gel. As expected, the extent of DNA digestion was exacerbated in cells treated with 0.005% MMS (S1A Fig); however, in contrast to other chimeras like Rad52-MN that requires 30 minutes in the absence of MMS [27], DNA digestion by Rfa1-MN was detected after 5 minutes of CaCl_2_ treatment. This digestion was observed both in cells maintained in G1 with α-factor and released into S phase for 30 minutes, although the kinetics of DNA digestion was faster in S phase cells (Fig 1A, compare 10 minutes digestion). This result is consistent with RPA localization at the transcribed regions of active genes [36]. However, RPA accumulates preferentially at replication forks where it protects ssDNA under unperturbed and stressed conditions, as determined by ChIP-seq of whole chromosomes and microscopy analyses of RPA foci in G1 and S phases [37–39]. Since ChEC preferentially detects DNA breaks at lineal molecules as the number of forks relative to the whole genome is low, we studied Rfa1-MN binding to replication forks by 2D/ChEC. In this assay, replication intermediates from ChEC-treated cells are analysed by 2-dimensional (2D) electrophoresis [28]. Activation of the MN activity of Rfa1-MN with Ca^2+^ digested all replication intermediates in less than a minute (Fig 1B), in sharp contrast with other chimeras like Rad52-MN or Rad27-MN that required several minutes for a partial digestion [27]. Thus, although Rfa1-MN can induce DSBs at linear molecules, it preferentially digests replication forks.

The Rfa1-MN chimera is expressed at the same steady state level as the non-tagged protein (Fig 1C) and is proficient in DNA damage tolerance (Fig 1D), DSB repair (total and mediated by HR) (Figs 1E and S1B), checkpoint activation (Fig 1F) and DNA replication (Fig 1G). Only a slight delay from G1 to G2/M was observed by FACS, although the budding index and doubling time were similar in *RFA1-MN* and wild-type cells (S1C Fig). Importantly, the fact that the *RFA1-MN* mutant behaves as the wild-type strain in the presence of high concentrations of MMS and hydroxyurea (HU) indicates that the chimera is also proficient in replication fork processivity and stability even under high replication stress conditions.

Remarkably, the genetic combination *RFA1-MN rad52∆* is lethal as determined by genetic analyses (Figs 1H and S1D). This synthetic lethality suggests that Rfa1-MN causes recombinogenic lesions that need to be repaired. HR deals with two different DNA lesions: DSB and replication associated-ssDNA. A major difference between them is that the former, but not the latter, can be repaired in *G2::cRAD52* cells that restrict the expression of Rad52 to G2/M [27]. We observed that the *RFA1-MN G2::cRAD52* strain grew as the wild type (Fig 1I), suggesting that Rfa1-MN causes DSBs. Rad52 essentiality in Rfa1-MN-expressing cells contrasts with the non-essential role for HR in the repair of mechanically- and HO endonuclease-induced DSBs where NHEJ can operate as a backup mechanism (Fig 1J) [40].

The simplest explanation to these results is that the amount of intracellular Ca^2+^ is sufficient to induce the nucleolytic activity of Rfa1-MN at a rate that has no effect on cell growth unless HR is absent. Although we cannot discard the formation of some DSBs at other regions, the preferential accumulation of RPA at replication forks [37–39], the high efficiency of Rfa1-MN to digest replication forks, and the essential role of HR for *RFA1-MN* cell viability suggest that most of these DSBs stem from Rfa1-MN–cut replication forks, preferentially at the lagging strand that accumulates most ssDNA/RPA (Fig 1K). We do not consider D-loops as a preferential target for Rfa1-MN-induced cleavage, because Rad52 does not promote, but instead prevents *RFA1-MN* cells lethality. Accordingly, *RFA1-MN* cells displayed a wild-type growth in the presence of high concentrations of HU even in plates enriched with CaCl_2_ to increase the rate of cleavage (S1E Fig).

**Fig 2:**
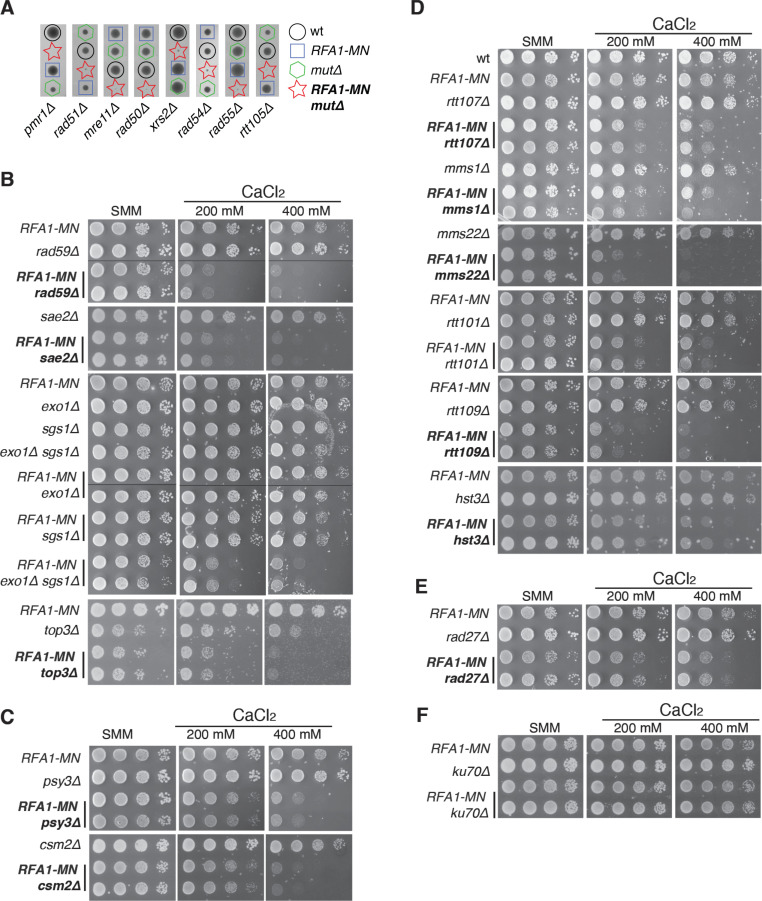
The repair of DSBs at replication forks requires DSB- and replication fork-specific HR activities. **(A)** Synthetic lethality of *RFA1-MN* with the indicated mutants as determined by tetrad analysis. **(B–F)** Effect of the indicated mutations in the growth of *RFA1-MN* cells as determined by spotting 10-fold serial dilutions of the same number of mid-log growing cells onto SMM medium without or with the indicated concentrations of CaCl_2_. The analyses were repeated at least twice with similar results. Mutants scored in the SGA screening are shown in bold.

If most DSBs stem from digested replication forks, the expression of Rfa1-MN should display synthetic growth defects with mutations previously identified as required for the rescue of broken replication forks. We tested the absence of Rad51, Mre11, Pol32, Mus81 and Pif1 (Fig 1L). Just a few double mutants *RFA1-MN rad51∆* and *RFA1-MN mre11∆* germinated leading to microcolonies that grew better after streaking in a new plate, likely by adaptation to grow with lower level of intracellular calcium or the selection of suppressors (Figs 2A and S2A). The growth of the *RFA1-MN* strain was affected to different extents in the absence of Pif1, Pol32 and Mus81, but only in plates enriched with CaCl_2_ (Fig 1L). The sensitivity of *mus81∆* to nick-induced fork breakage is severely aggravated in the absence of Yen1 [17,21], an endonuclease that participates with Mus81 in the resolution of single Holiday junctions (HJs) [41] as those expected from the merging of a BIR-associated migrating D-loop with a converging fork. The absence of Mus81 and Yen1 strongly impaired cell growth in Rfa1-MN-expressing cells, although without being lethal (Fig 1L). In conclusion, Rfa1-MN provides a genetic system to search for factors involved in the repair of DSBs at replication forks.

### Identifying functions required for the repair of DSBs at replication forks

To search for factors required for the repair of DSBs at forks, we followed a Synthetic Genetic Array (SGA) analysis based in the crossing of an ordered array of null mutants to a strain harbouring the query allele *RFA1-MN* and specific markers such that the meiotic progeny with both the *RFA1-MN* allele and the null mutation can be scored for fitness [42]. This customized array encompasses 358 null mutants selected according to their confirmed or putative connection with the DNA damage response (S1 Table). The loss of viability or cell fitness was scored in plates without and with 400 mM CaCl_2_ to increase the sensitivity of the screening. We obtained 62 hits, out of which 6 were wild type for the expected null mutation and 4 could not be validated by PCR (S2 Table). For manual inspection of these genetic interactions, we crossed the original *RFA1-MN* strain with each null mutant (including those scored as synthetically lethal), analysed genetically the dissected spores and studied the loss of fitness by drop assays in medium without and with different concentrations of CaCl_2_. This study revealed 44 genes that are required to a greater or lesser extent for the viability of Rfa1-MN expressing cells (S2 Table). Except for *rad52∆* and *pmr1∆* mutants, we obtained double mutants with the *RFA1-MN* allele for the rest, including those scored as synthetically lethal in the SGA screening. These “lethal” mutants included *rad51∆* and *mre11∆* and displayed a similar behaviour (Figs 2A and S2A). Pmr1 is a Ca2+/Mn2+ ATPase required for Ca^2+^ transport to Golgi whose null mutant accumulates excess Ca^2+^ ions [43], which is likely causing a lethal number of broken forks.

### The repair of DSBs at replication forks requires DSB- and replication fork-specific HR activities

Apart from the mediator Rad52, we scored as synthetically lethal the MRX complex (Mre11, Rad50 and Xrs2), the recombinase Rad51 and its helpers Rad55 and Rad54 (Fig 2A and S2 Table), which are essential components of the HR machinery dealing with DSBs [44]. The recombination protein Rad59 was also found in the screening but only in the presence of CaCl_2_ (Fig 2B), which is consistent with the minor effect of *rad59∆* in HR in the presence of Rad51 [45].

Another hit scored as synthetically lethal was Rtt105 (Fig 2A and S2 Table), despite it encodes a chaperone involved in the transfer to the nucleus and deposition at ssDNA of the RPA complex. However, the reduction in the level of RPA at forks in *rtt105∆* cells is slight under normal conditions [37,46], which might explain why Rfa1-MN is inducing DNA damage as inferred from the lethality. The synthetic lethality of *RFA1-MN rtt105∆* cells might be related to the function of Rtt105 in HR – where it facilitates Rad51 loading at ssDNA for DSB-induced gene conversion and BIR [46] – and to a lesser extent to the role of RPA in replication fork stability and checkpoint activation (see below). Actually, the DSB repair defect in *rtt105∆* is almost as strong as that displayed by *rad52∆* (S2B Fig). Remarkably, ssDNA stabilization by RPA is particularly critical for HR mechanisms that involves long-lived ssDNA intermediates, in particular BIR [47]. Altogether, these results demonstrate that the MRX complex, the Rad51/ssDNA nucleofilament and the factors that promote its assembly are essential for the repair of DSBs at forks.

During DSB-induced HR, Sae2 promotes the nuclease activity of the MRX complex in the initial processing of DSB ends to generate short stretches of 3’-ended ssDNA. This DNA resection is completed by the nuclease and helicase activities of Exo1 and Sgs1/Dna2 through complementary mechanisms [48]. In contrast to MRX, Sae2 was identified in the screening only in the presence of CaCl_2_ (Fig 2B). To address the relevance of long resection, we analysed the effect on cell growth of the single and double *exo1∆* and *sgs1∆* mutants in combination with *RFA1-MN*. Only the *RFA1-MN exo1∆ sgs1∆* displayed a loss of growth in the presence of CaCl_2_ (Fig 2B). Again, the lack of long resection was not essential. It is worth noting the lack of effect of *sgs1∆*, because the helicase Sgs1 is required for the dissolution of double HJ (dHJ) and sister-chromatin junction (SCJ) structures by the Top3/Sgs1/Rmi1 complex [49,50]. Remarkably, another hit of the screening was the topoisomerase Top3, scored as lethal (S2 Table). Since we could not validate the collection mutant by PCR, we generated the *RFA1-MN top3∆* mutant by genetic cross. The lack of Top3 caused a growth defect in the presence of CaCl_2_ (Fig 2B), suggesting a Sgs1-independent role in the rescue of broken forks.

Two components of the helper Shu complex (Psy3 and Csm2) were scored as synthetically sick in the presence of CaCl_2_ (Fig 2C). The Shu complex is also involved in Rad51 filament formation [51], but in contrast to the aforementioned HR mutants, *shu* mutants are primarily sensitive to MMS-induced replication-associated ssDNA lesions but not to DSB-inducing agents [52,53].

Another functional genetic hub identified in the SGA screening is formed by *rtt109∆*, *rtt107∆*, *mms1∆* and *mms22∆* (Fig 2D) [54,55]. Rtt109 is a histone acetyltransferase that acetylates histone H3 at lysine 56 (H3K56) [56,57], which in turn facilitates histone H3/H4 deposition by increasing its interaction with chromatin assembly factors CAF and Rtt106 [58]. This pathway is stimulated after ubiquitylation of the acetylated histone by the Rtt101^Mms22/Mms1^ complex [59], which is associated with the replisome during S phase [60]. The involvement of the ubiquitin ligase Rtt101 was confirmed by manual inspection of *RFA1-MN* *rtt101∆* mutants (Fig 2D). At chromatin, the H3K56ac/ Rtt101^Mms22/Mms1^ pathway promotes the recombinational repair of replication-associated ssDNA lesions but not of DSBs [61–64]. The chromatin assembly and recombinational functions of H3K56ac can be separated in a double mutant *cac1∆* *rtt106∆* (Cac1 encodes the largest subunit of the CAF complex) because the ability of H3K56ac to stimulate nucleosome assembly depends on CAF and Rtt106 [58], whereas its ability to promote HR is independent of CAF and Rtt106 [65–67]. The triple *RFA1-MN* *cac1∆* *rtt106∆* was hardly affected even at high concentrations of CaCl_2_ (S2C Fig), suggesting that chromatin assembly does not play a major role in the repair of DSBs at forks. This was further confirmed by testing the *spt16-m* allele (alone or in combination with *cac1∆* *rtt106∆*), which impairs the replication-coupled nucleosome activity of the FACT complex [68], and the *pol1-2A2*, *mcm2-3A* and *dpb3∆* alleles, defective in the transfer of parental histones to nascent strands [69–71]. Only the *RFA1-MN* *pol1-2A2* mutant displayed a weak effect in plates with high CaCl_2_ concentrations (S2C Fig), which might be related to a subtle defect at its polymerase activity. The recombinational role of H3K56ac in the repair of DSBs at forks was further supported by the finding of the *hst3∆* mutant in the screening (Fig 2D). Hst3 forms with Hst4 a Sirtuin complex that deacetylates chromatin-deposited H3K56ac once the replicative DNA damage is repaired [72,73]. Alternatively, the growth defect of *RFA1-MN* *hst3∆* cells might be due to the inhibitory effect of H3K56 hyper-acetylation on DNA synthesis during BIR [74].

Rtt109 also facilitates the recruitment to stalled forks of Rtt107 through a H3K56ac-independent mechanism [75]. Rtt107 is a protein that acts as a scaffold for three genome maintenance complexes: the Rtt101^Mms22/Mms1^ ubiquitin ligase, the Slx4 scaffold for the Slx1 and Mus81-Mms4 nucleases and the Smc5/6 SUMO ligase [76,77]. To address the role of the Smc5/6 SUMO ligase we tested the *smc6–56* allele and observed no effect on the growth of Rfa1-MN expressing cells (S2D Fig). This result is consistent with the dispensability of Sgs1 in broken fork repair, as the Smc5/6 complex is also required for MMS-induced SCJ dissolution and DSB repair [78]. We have further discarded a role for these structures in the repair of DSBs at forks by analysing the effect of a *rad18∆* mutant, defective in PCNA ubiquitylation and replication stress-associated SCJ formation (S2D Fig) [79,80]. Therefore, the role of Rtt109 and Rtt107 on the growth of Rfa1-MN expressing cells is associated with the ubiquitylation and nuclease functions of Rtt101^Mms22/Mms1^ and Mus81, respectively. In line with the latter function, it is particularly interesting the finding of Rad27 in the SGA screening (Fig 2E), as the physical and functional interactions of this endonuclease with Slx4-Mus81 (including synthetic lethality of the double null mutants) might be critical for the resolution of intermediates during replication stress [81]. Altogether, these results indicate that the repair of DSBs at forks requires replication fork-specific HR activities.

These results demonstrate that DSB- and replication-fork-associated HR functions participate in the repair of broken replication forks. On the contrary, NHEJ seems not to be required for broken fork repair because neither the Ku70/Ku/80 complex nor Nej1 were scored as positive hits. This was confirmed by manual inspection of a *RFA1-MN ku70∆* mutant (Fig 2F).

### Checkpoint factors facilitate the repair of DSBs at forks

**Fig 3:**
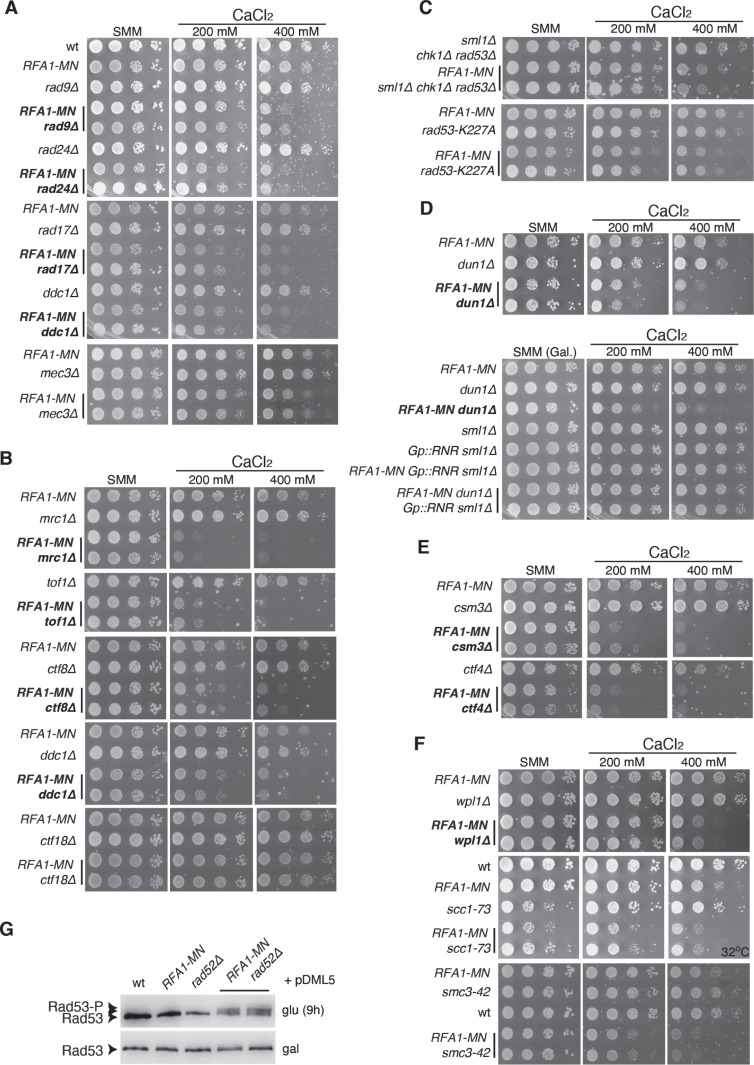
Checkpoint, replication fork stability factors and cohesins facilitate the repair of DSBs at forks. **(A–F)** Effect of the indicated mutations in the growth of *RFA1-MN* cells as determined by spotting 10-fold serial dilutions of the same number of mid-log growing cells onto SMM medium without or with the indicated concentrations of CaCl_2_. Mutants scored in the SGA screening are shown in bold. **(G)** Checkpoint activation of the indicated strains transformed with plasmid pMDL5 (expressing *RAD52* under control of the *GAL1* promoter) in galactose or after 9 hours in glucose, as determined by western blot against Rad53. The analyses were repeated at least twice with similar results.

The second functional group involved in the repair of DSBs at forks encompasses several DNA damage checkpoint (DDC) (Rad9, the 9-1-1 complex (Ddc1/Mec3/Rad17) and its loader Rad24) (Fig 3A) and DNA replication checkpoint (DRC) factors (Mrc1, Tof1, and the Ctf8 and Dcc1 components of the PCNA loader RFC/Ctf18/Ctf8/Dcc1 (Ctf18-RFC complex)) (Fig 3B) [82,83]. All these factors have additional functions apart from checkpoint activation: Rad9 protects DSBs from premature resection [84]; the 9-1-1 complex participates in DDT and replication-coupled nucleosome assembly [85,86]; Mrc1 and Tof1 have roles in coupling helicase and polymerase activities, sister chromatid cohesion (SCC), and in the case of Tof1, stable fork pausing at replication fork blocks [87–91]; the Ctf18-RFC complex is required for replication fork stability upon stress and SCC [92,93]. In this case, since the annotated *ctf18∆* mutant was wild type in the collection, we generated a new one and observed that it did not affect Rfa1-MN growth even at high CaCl_2_ concentrations (Fig 3B). This result is consistent with previous observations showing that Ctf18, Ctf8 and Dcc1 are required for MMS and HU resistance, but only Ctf8 and Dcc1 are required for ionizing radiation (IR) and UV light resistance [94]. Since the whole complex is required for the aforementioned functions including DRC activation, these results suggest that Ctf8 and Ddc1 promotes broken fork repair by a not-yet defined function.

To test the effect of specifically eliminating the DNA damage and replication checkpoints, we generated a *RFA1-MN* strain lacking the checkpoint effectors Rad53 and Chk1 (*RFA1-MN sml1∆ rad53∆ chk1∆*). This mutant displayed a subtle growth defect at high concentrations of CaCl_2_ (Fig 3C). The kinase Dun1, target of Rad53, was also identified in the screening as synthetically sick in the presence of CaCl_2_ (Fig 3D). A major role of Dun1 is to increase the levels of dNTPs during DNA replication and in response to DNA damage and replication stress [82,83]. According with this function, the calcium-induced defect is exacerbated in the presence of HU (S3A Fig), and more importantly, concomitant over-expression of the ribonucleotide reductase (RNR) complex that catalyses the rate-limiting step in dNTP synthesis and elimination of the RNR inhibitor Sml1 rescued the growth defects of the *RFA1-MN dun1∆* mutant (Fig 3D). To determine if the partial checkpoint defect is exclusively due a deficit of dNTPs, we tested the kinase-deficient *rad53-K227A* mutant, which maintains wild-type dNTP levels [95]. This mutant also reduced the viability of Rfa1-MN-expressing cells at high concentrations of CaCl_2_ (Fig 3C). The lack of effect of the checkpoint mutants under normal Ca^2+^ conditions is consistent with the unphosphorylated state of Rad53 (Fig 1F). Unfortunately, we could not assess the checkpoint response to high Ca^2+^ concentration because this condition caused a transient arrest in S phase in which the checkpoint was hardly activated (Fig S3B and C), and at later times (after 2–3 hours), CaCl_2_ precipitates technically impeding western blot analysis. Therefore, we explored the checkpoint response under conditions of defective repair. Repression of *RAD52* in the *RFA1-MN rad52∆* (pDML5) strain led to an accumulation of phosphorylated Rad53 (Fig 3G). This effect is partial, likely as a consequence of the basal expression of the *GAL1* promoter that allows *RFA1-MN GALp::RAD52* cells to slowly grow in glucose-containing medium (S1D Fig). Therefore, checkpoint activation facilitates cell growth under conditions that cause multiple broken replication forks, especially in HR defective cells.

### Replication fork stability factors and cohesins facilitate the repair of DSBs at replication forks

The lack of Mrc1 and Tof1 caused a severe growth defect in Rfa1-MN expressing cells as compared with the rest of checkpoint mutants (Fig 3A–C), suggesting a role for SCC and/or replication fork stability in the repair of DSBs at forks. Supporting this possibility, another hit of the SGA screening was Csm3 (Fig 3E), which together with Tof1 constitute the fork protection complex (FPC) that participate in both SCC and coupling of the helicase and polymerase activities at the fork upon replication stress [87–91]. The involvement of these processes is also supported by the finding of *ctf4∆* in the SGA screening (Fig 3E). Ctf4 is a replisome component that physically bridges the helicase with the polymerase α (Polα) and other factors including Chl1 and Dia2, which in turn link DNA synthesis to SCC and fork assistance to stress, respectively [96–98]. Indeed, we have also found Dia2 in our screening, although a mutant lacking the Ctf4 interacting domain did not affect *RFA-MN* viability (S4A Fig). Additional roles of Ctf4 in MMS-induced SCJ and parental histone recycling are unlikely required for broken fork repair according to the lack of effect of the *rad18∆* and chromatin assembly mutants, respectively (S2C and D Fig) [69,99].

A direct involvement of cohesin dynamics in the repair of broken forks is supported by the identification in the SGA screening of Wpl1 (Fig 3F), a factor needed for the removal of cohesive cohesins from chromatin [100]. To address if a defect in SCC can also impair the repair of broken forks, we tested two thermosensitive mutants affected in subunits of the cohesin complex (*scc1–73* and *smc3–42*) (Fig 3F) [101,102]. The addition of CaCl_2_ to the medium improved the growth of the *scc1–73* mutant, suggesting an activation of stress chaperones. Importantly, the double mutants *RFA1-MN scc1–73* and *RFA1-MN scm3–42* displayed growth defects in the presence of CaCl_2_. This indicates that cells need a sufficient pool of functional cohesins for the repair of DSBs at forks.

It is worth to note that only the absence of some SCC factors has an effect on the rescue of DSBs at forks. For instance, although Mrc1 and the Ctf18-RFC complex are involved in the Scc2/Scc4-dependent cohesin de novo loading, the defects of *ctf8∆* and *dcc1∆* on broken fork repair cannot be attributed to this function because it requires the whole complex [90]. Likewise, the conversion of cohesins at non-replicated DNA ahead of the fork into cohesive structures behind the fork requires Ctf4, Tof1/Csm3 and the helicase Chl1 [90], but the lack of the latter has no effect on the growth of Rfa1-MN expressing cells (S4B Fig).

### Shortening of G1 compromises the rescue of broken replication forks

**Fig 4:**
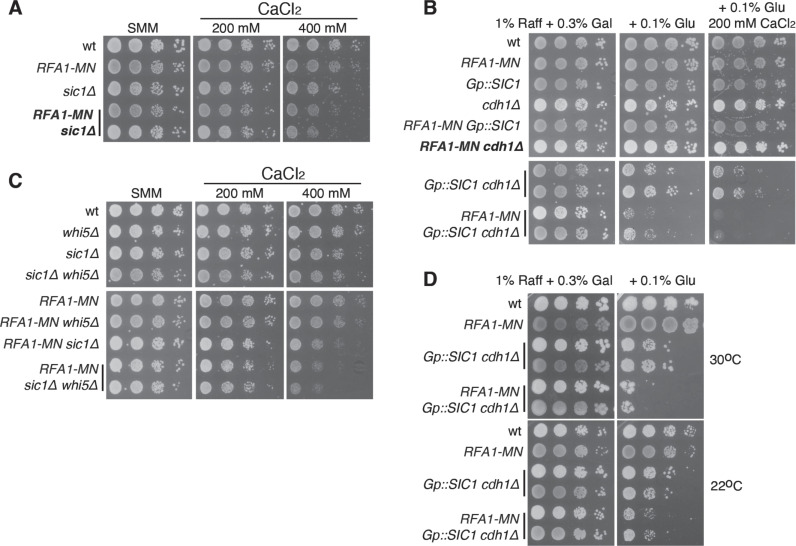
G1 length facilitates the repair of DSBs at forks. **(A–D)** Effect of mutations that shorten the length of G1 in the growth of *RFA1-MN* cells as determined by spotting 10-fold serial dilutions of the same number of mid-log growing cells onto SMM medium without or with the indicated concentrations of CaCl_2_. To study a *sic1 cdh1* mutant (the double null mutant is lethal), we employed a strain where *SIC1* is under control of the *GAL1* promoter and analysed the effect of Rfa1-MN expression under permissive (0.3% galactose) and semi-permissive conditions (0.1% glucose) in the absence and presence of 200 mM CaCl_2_. The analyses were repeated at least twice with similar results. Mutants scored in the SGA screening are shown in bold.

The null mutants *sic1∆* and *cdh1∆* were scored as synthetically sick in the presence of CaCl_2_, but only *sic1∆* was confirmed in the drop test displaying a subtle effect at 400 mM CaCl_2_ (Fig 4A and B). Sic1 and Cdh1 control the G1/S transition through complementary mechanisms; Sic1 is a cyclin-dependent kinase (CDK) inhibitor, whereas Cdh1 is an activator of the anaphase-promoting complex (APC) that promotes cyclin degradation [103]. In accordance with their overlapping functions, the double mutant *sic1∆* *cdh1∆* causes lethality due to a premature entry into S phase and insufficient number of licensed origins [104,105]. However, it is possible to get a G1 phase shorter than the one displayed by the single mutants in a *cdh1∆* strain with *SIC1* under control of the *GAL1* promoter [104]. Under semi-permissive conditions, the expression of Rfa1-MN in a *cdh1∆ Gp::SIC1* strain caused severe growth defects and lethality in the absence and presence of 200 mM CaCl_2_, respectively (Fig 4B). Likewise, the lack of Whi5, a transcriptional repressor of cell-cycle activators controlling the entry into S phase [106], reduced the growth of the *RFA1-MN* *sic1∆* strain in the presence of CaCl_2_ (Fig 4C). These results suggest that the shortening of the G1 phase compromises the rescue of broken forks, likely by reducing the number of licensed origins. Accordingly, slowing down the cell cycle by plating cells at 22 ^o^C slightly rescued the growth defects of *RFA1-MN* *cdh1∆* *Gp::SIC1* cells (Fig 4D).

## Discussion

**Fig 5:**
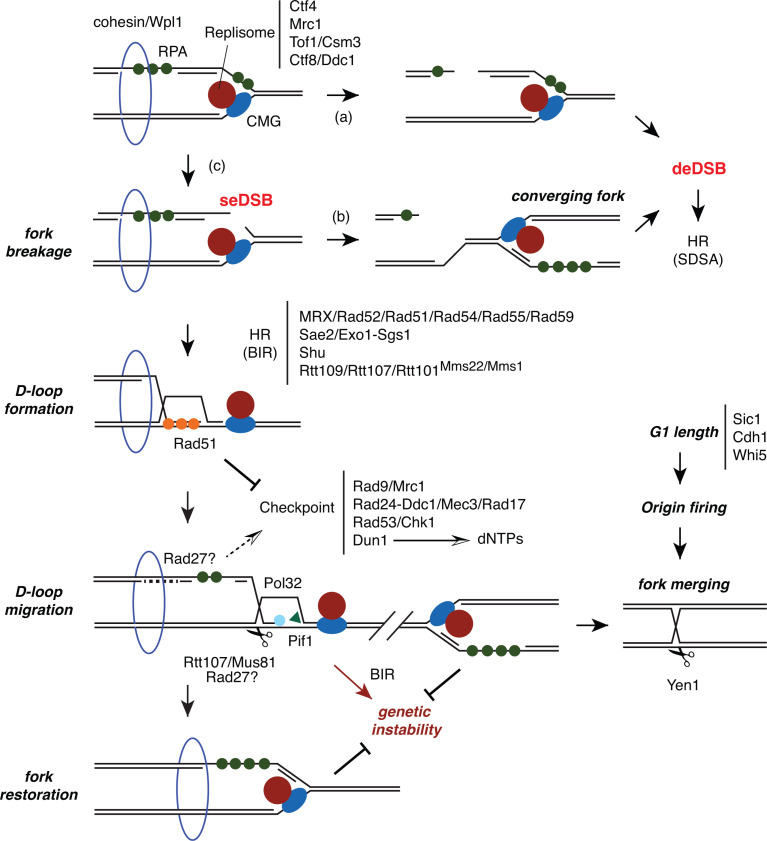
Proposed mechanisms for the repair of a DSB at forks. After fork breakage by Rfa1-MN, HR might be operating at a deDSB generated either between Okazaki fragments (A) or by collapse of a converging fork with the gap left at the non-broken strand **(B)**, or at a seDSB generated after cleavage of the fork junction, more likely at the lagging strand **(C)**. In response to seDSBs, the core HR machinery (with the help of fork-associated HR factors) would promote the invasion of the sister chromatid, generating a D-loop structure that primes a conservative, error-prone replication by a migrating bubble. This BIR-like restart mechanism would be facilitated by cohesins, checkpoint activation, Dun1-mediated increase in dNTPs, and replisome components that would be retained at the proximity of the D-loop for the stability of the migrating D-loop or, alternatively, the conversion of this structure into a canonical fork upon the activity of the Mus81 nuclease. The formation of the D-loop structure would prevent checkpoint activation and inhibition of late replication origins. The activation of these origins would also prevent BIR-associated genetic instability by fork merging with the D-loop structure and subsequent Mus81/Yen1-dependent HJ resolution. In line with this later mechanism, regulation of G1 length by Sic1, Cdh1 and Whi5 would facilitate the rescue of broken replication forks by ensuring a sufficient number of active origins, especially in response to massive fork breakage or fork breakage at specific regions like the end of chromosomes or common fragile sites.

In this study, we have generated a chimera of the largest subunit of the RPA complex with the MN that preferentially generates DSBs at replication forks and searched for mutants affected in their repair. Several major conclusions can be withdrawn. First, the core HR factors required for the detection and processing of DSBs to form a ssDNA/Rad51 filament are essential, as shown for nick-induced fork breakage [17,21]. HR might be operating at a 1) deDSB generated either between Okazaki fragments (Fig 5A) or 2) by collapse of a converging fork with the gap left at the non-broken strand (Fig 5B), or 3) at a seDSB generated after cleavage of the fork junction, more likely at the lagging strand (Fig 5C). In the latter case, a direct cleavage at the fork junction would likely disrupt the replisome structure thus preventing fork progression. The scarce formation of dHJ structures by double strand break repair (DSBR) suggested by the null effect of *sgs1∆* and *smc5–56* mutants could be due to a low accumulation of deDSBs. Alternatively, it might reflect a preferential repair by synthesis-dependent strand annealing (SDSA), as observed for nick-induced deDSBs [22,23]. The accumulation of seDSBs by Rfa1-MN is supported by the finding of BIR-associated factors, fork-associated HR factors, and replisome components for their repair. These requirements are consistent with a BIR-like fork restart mechanism, even it is unlikely that extensive synthesis by BIR suffices for cell viability, as inferred from the growth defects of the triple mutant *RFA1-MN mus81∆ yen1∆* (lacking the enzymes required for D-loop-to-fork conversion and HJ resolution after D-loop/fork merging) [17,21]. The growth defect of this mutant also reinforces the accumulation of seDSBs in *RFA1-MN* cells because Mus81 and Yen1 are not required for SDSA and are a backup mechanism for DSBR [8]. Since the arrival of a converging fork would lead to re-replication if the gap left at the non-broken strand has been repaired, seDSB invasion and D-loop formation could be a mechanism to ensure merging with the converging fork and genetic stability.

Our results also show that the DNA resection factors Sae2 and Sgs1/Exo1 are not essential, which might be explained by the minimal resection that replication-born seDSBs require for strand invasion [107] and the likely preferential cleavage of the lagging strand by Rfa1-MN that would generate a 3’-ended ssDNA. In this regard, the essentiality of the MRX complex, also observed for Mre11 but not for its nuclease activity in nick-induced fork breakage [21], is more likely related to its replication fork stability and sister chromatid tethering activities [108,109]. Sae2 and Sgs1/Exo1 might be preferentially required for those cases in which Rfa1-MN cuts at the leading strand, which would lead to the formation of blunt or 5’-ssDNA ends. Sae2 would facilitate the removal of the Ku complex from these intermediates, in accordance with the increase in nick-induced BIR events in yeast cells lacking Ku70 [23].

Finally, our screening has revealed a Sgs1-independent role for Top3 in the rescue of broken forks. Apart from its dHJ and SCJ dissolution activity in conjunction with Sgs1, the Top3/Rmi1 complex dissolves nascent Rad51-mediated D-loops *in vitro* that might explain the extreme growth defect and hyper-recombination phenotype of the *top3∆* mutant [110]. This anti-recombinogenic activity is in apparent contradiction with the essential function of HR in the rescue of broken forks. However, this activity might be important to prevent template switching during BIR, a genotoxic event that can lead to chromosome rearrangements [13].

Second, the repair of DSBs at forks is facilitated by HR factors that are specific of stalled replication forks. In particular, it requires the Shu complex and the Rtt109/H3K56ac/Rtt101^Mms22/Mms1^ pathway, which promote the recombinational repair of replication-associated ssDNA lesions but not of DSBs [52,53,61–64]. The Shu complex facilitates the formation of the Rad51 filament during ssDNA gap filling by physically recruiting the Rad55/Rad57 heterodimer to stalled forks [111], whereas the Rtt109/H3K56ac/ Rtt101^Mms22/Mms1^ pathway seems to uncouple the DNA polymerases from the CMG helicase to facilitate recombination [60,66]. These requirements suggest that the recombinational repair of broken forks occurs in the context of DNA-bound replication factors, which is supported by the finding of several replisome components in our screening. A concomitant study searching for factors involved in the repair of nick-induced DSBs uncovered a role for the Rtt109/H3K56ac/ Rtt101^Mms22/Mms1^ pathway only when the nick is at the template for the leading strand [21]. Whereas a nick at the template for the lagging strand can be bypassed generating a deDSB behind the fork that is repaired by SDSA, a nick at the leading template can generate a seDSB. Thus, Rtt109/H3K56ac/ Rtt101^Mms22/Mms1^ might not be specifically required for a DSB at the lagging strand, but for the BIR-mediated restart of a seDSB. Accordingly, this pathway was identified – together with the core HR factors and the MRX complex – among the requirements for the repair of seDSBs generated by rNMP-induced nicks, regardless of their position at the leading or lagging strand [112].

Third, the rescue of DSBs at forks is associated with unstable replication intermediates, as inferred from the loss of viability of Rfa1-MN cells lacking Ctf4, Mrc1, Ctf8, Dcc1 or the Tof1/Csm3 FPC. These factors, like Pol32, are dispensable for unperturbed DNA replication. They participate in SCC, stable fork pausing and coupling of the helicase and polymerase activities at the fork upon replication stress [87–93,96,97,113]. The involvement of cohesins is supported by the finding of Wpl1 in the screening, and further demonstrated with specific thermosensitive alleles of the cohesin complex. This finding is expected as holding sister chromatids together by the cohesin complex is needed for the repair of both canonical DSBs and stalled replication forks [114,115]. Another replisome component partially required for *RFA1-MN* cell viability is Rad27, a nuclease that participates in the maturation of the Okazaki fragments [116]. The rescue of nick-induced seDSBs is associated with high rates of mutagenesis and template switching events [17]. BIR studies with ectopic HR systems suggest that mutagenesis stems from an accumulation of ssDNA at the lagging strand behind the migrating D-loop structure [117]. Efficient processing of this strand might be important to prevent excess ssDNA that would destabilise this replication intermediate. Alternatively, the physical and functional interactions of Rad27 endonuclease with Slx4-Mus81 might be critical for the processing of the D-loop [81]. It is important to remark that the growth defects observed in the absence of replication factors are unlikely due to an accumulation of ssDNA and a higher probability of fork breakage by Rfa1-MN or to the additive effects of fork cleavage and replication stress because the *RFA1-MN* mutant behaves both with and without calcium as the wild-type strain even in the presence of high concentrations of MMS and HU that strongly impair cell growth (Figs 1D and S1E).

The establishment of cohesion is achieved through two partially complementary mechanisms: the conversion of cohesins associated with unreplicated DNA ahead of the fork into cohesive structures behind the fork (dependent on Ctf4, Tof1/Csm3 and Chl1) and the loading of nucleoplasmic cohesins onto fork-associated nascent DNA (dependent on the cohesin loader Scc2/Scc4 and the Ctf8-RFC complex) [90]. Our results show that the absence of Chl1 or Ctf18 does not impact the repair of DSBs at forks. Thus, the role of Ctf4, Mrc1, Ctf8, Dcc1 and the Tof1/Csm3 complex in broken fork repair cannot be explained just by a defect in SCC. Conservative replication associated with D-loop migration uncouples the leading and lagging strands [118]. In a canonical fork, they are coupled through physical interactions of Ctf4, Mrc1 and Tof1/Csm3 with the CMG helicase and the DNA polymerases Pol ε and Pol α [87,119]. A potential rearrangement of these interactions in the migrating D-loop structure might be related to the recombinational role of the Rtt109/H3K56ac/ Rtt101^Mms22/Mms1^ pathway, as the sensitivity to replication stress of cells lacking this pathway can be suppressed by mutations in Ctf4, Mrc1, Dpb4 (Pol ε) or Mcm6 that uncouple the CMG helicase from the DNA polymerases [60,66]. A screening for factors involved in the rescue of oncogene-induced stressed forks uncovered, together with the BIR proteins Rad52 and PolD3 (human ortholog of Pol32), the FPC components Tipin and Timeless (human orthologs of Tof1 and Csm3) [120], suggesting a conservation of these factors.

Fourth, shortening of the G1 phase compromises the rescue of broken forks, as inferred by the inverted correlation between G1 length and cell growth defects in *RFA1-MN* cells lacking different inhibitors of the G1/S transition. Converging forks limit the mutagenicity associated with the repair of a nick-induced DSB, likely by merging with the D-loop [17]. Since a premature entry into S phase reduces the number of licensed origins [104,105], the severe growth defects of Rfa1-MN-expressing cells in combination with a shortening of G1 might be due to a reduction in the number of active forks that could rescue the broken forks. In yeast and cancer cells, premature entry into S phase by CDK deregulation in G1 causes a reduction in the number of active replication origins and genome instability. This instability has been proposed to result from a higher frequency of fork collapse and/or the entry into mitosis with incompletely replicated genomes [121]. Our result suggests that it may also arise from unrepaired broken forks and/or excess BIR-induced mutagenesis.

Fifth, the repair of DSBs at forks by HR is an efficient process. The lethality of the double mutant *RFA1-MN rad52∆* suggests that at least one fork per cell cycle is cut by the chimera. However, *RFA1-MN* cells did not display growth defects and the checkpoint was not required except at high levels of CaCl_2_ (consistent with an accumulation of DSBs and/or BIR-associated ssDNA) or in the absence of Rad52 (consistent with the accumulation of DNA resection-mediated ssDNA at broken forks when strand exchange is abolished [107]). Interestingly, we have found Dun1 in our screening and demonstrated that the growth defect is due to a reduction in the levels of dNTPs, in line with the Dun1-dependent increase in both dNTPs and mutagenesis observed during BIR [12].

Taking into account our results and previous studies, we propose the following model for the repair of seDSBs at forks (Fig 5). A Rad51/ssDNA nucleofilament formed at the broken nascent strand would invade the sister chromatid in the context of the replisome machinery with the help of stalled fork-associated HR factors, leading to the formation of a D-loop structure. This invasion step has to occur behind the CMG helicase, which may be retained at the proximity together with replisome components for further restoration of the replication fork. These replisome components might be required for the stability of the migrating D-loop (whose advance would require Pol32 and Pif1) and/or the conversion of this structure into a canonical fork upon the activity of Mus81. Cohesins would also contribute to the stability of this structure and/or to the previous invasion step. Replication fork restart by this BIR-like mechanism is associated with high levels of mutagenesis and template switching events. This genetic instability would be potentially restricted by specific factors like Rad27 and Top3, the conversion of the D-loop into a canonical fork and the merging with a converging fork, favoured by the licensing of sufficient replication origins during G1 phase. In this context, the nucleases Mus81 and Yen1 might also be required for the resolution of the HJ structure generated after fork merging. A major observation of this study is the essential role of the HR machinery. We think that HR-mediated strand exchange would not only promote replication fork restart, but would also prevent inhibition of origin firing by checkpoint activation, as replication is required for the rescue by converging forks.

Apart from the positive hits, some of which requires further investigation to understand their connection with broken fork repair (S4C Fig), our screening revealed a scarce impact by the loss of chromatin factors. This is unexpected taking into account their relevance during DNA replication and DSB repair [122]. Mutants affecting the deposition of newly and parental histones during replication hardly affected the viability of Rfa1-MN-expressing cells. Likewise, histone chaperones that participate in replication-independent nucleosome exchange (HIR, Nap1, Chz1) and chromatin remodelling factors (INO80, SWR1, ISW1, ISW2, SWI/SNF and RSC) were negative hits in the screening, with the exception of Chd1 (S2 Table and S4D Fig). Although the involvement of chromatin in the repair of DSBs at forks requires a more detailed analysis, one possibility to explain its low impact is that the partially disassembled nucleosome structure at the advancing fork facilitates the accessibility of the repair machinery.

A limitation of our system is that many of the hits were identified by adding CaCl_2_ to the medium to increase the number of broken forks. This sudden increase in cytosolic Ca^2+^ triggers the reprogramming of Ca^2+^ transporters to restore physiological levels [123,124]. Thus, we cannot rule out that some of the hits might be specific of this Ca^2+^ stress context. Moreover, the cleavage likely occurs preferentially at the lagging strand, where RPA tends to accumulate. It will be interesting to determine the effect of the analysed mutants if the DSB occurs preferentially at the leading strand.

In summary, our results provide new genetic requirements for the repair of broken forks and highlight the significance of error-prone BIR restart, fork restoration from BIR-intermediates and rescue by converging forks. Specifically, recombination factors associated with replication forks, replisome components critical for fork stability, and regulators of the G1 phase may potentially control the efficiency of these pathways and the impact of broken fork repair on genome integrity, especially in regions with low density of active origins like the end of chromosomes and common fragile sites (CFS) in mammalian genomes [125,126], which relies on BIR-like mechanism: MiDAS (mitotic DNA synthesis) and ALT (alternative lengthening of telomeres) [127]. Future molecular experiments will be required to test the different scenarios inferred from our genetic analyses.

## Materials and methods

### Yeast strains, plasmids and growth conditions

All *Saccharomyces cerevisiae* strains used are haploid derived from BY4741 or W303. Yeast strains used in this study are listed in S3 Table. Most strains were generated by genetic crosses. Tagged and deletion strains were constructed by a PCR-based strategy [128]. pDML5 is a *URA3*-based centromeric plasmid that expresses *RAD52* from the galactose-inducible *GAL1* promoter. pGAL-HO is a *URA3*-based multicopy plasmid expressing the endonuclease HO from the *GAL1* promoter [129]. Yeast cells were grown in supplemented minimal medium (SMM) at 30 °C except for liquid cultures supplemented with 400 mM CaCl_2_, which was performed at 26 °C to reduce Ca^2+^ precipitation. For G1 synchronization, cells were grown to mid-log phase and α-factor was added twice at 60 min intervals at either 1 (*BAR1* strains) or 0.5 μg/ml (*bar1∆* strains). Then, cells were washed three times and released into fresh medium with 50 μg/ml pronase.

### Synthetic genetic array analysis

The synthetic genetic array analysis (SGA) was performed as reported with some modifications [42]. The query strains (*RFA1-MN::NAT* and control *trp1∆::NAT*) were crossed with a customized array of null mutants using a manual replicator. The double mutants with *RFA1-MN* were scored as synthetically lethal or synthetically sick by comparing their growth with the double mutants with *trp1∆::NAT* on the SD_MSG_-His/Arg/Lys-canavanine-thialysine-G418-nourseothricin plates. To address the effect of Ca^2+^, both sets of double mutants were first replica plated to SMM and then to SMM supplemented with 400 mM CaCl_2_.

### DNA damage sensitivity

The sensitivity to Rfa1-MN expression, zeocin, MMS, HU and HO expression was determined by spotting ten-fold serial dilutions of the same number of mid-log growing cells onto SMM medium without or with CaCl_2_, zeocin, MMS and HU, or onto glucose and galactose-containing medium (HO). For ionizing radiation sensitivity spotted cells were irradiated and then grown under unperturbed conditions. All analyses were repeated at least twice with similar results.

### Cell growth analyses

Cell cycle was followed by DNA content. DNA content analysis was performed by flow cytometry as reported previously [130]. Cells were fixed with 70% ethanol, washed with phosphate-buffered saline (PBS), incubated with 1 mg of RNaseA/ ml PBS, and stained with 5 μg/ml propidium iodide. Samples were sonicated to separate single cells and analyzed in a FACSCalibur flow cytometer. The budding index (percentage of cells with bud) was determined by counting 100 cells at each time point and replicate. The doubling time was calculated by measuring the OD_600_ from exponentially growing cultures as previously described [131].

### *In vivo* ChEC and ChEC/2D analyses

Chromatin endogenous cleavage (ChEC) and ChEC/2D analyses of *RFA1-MN* cells were performed as reported [28]. Briefly, cells grown under the indicated conditions were arrested with sodium azide (0.1% final concentration). For cleavage induction, cells were permeabilized with digitonin and incubated with 2 mM CaCl_2_ at 30 °C under gentle agitation. For ChEC analyses, total DNA was isolated and resolved into 0.8% TAE 1× agarose gels. To analyse replication intermediates (ChEC/2D), total DNA was extracted as detailed, digested with *Eco*RV and *Hin*dIII, resolved by neutral/neutral two-dimensional (2D)-gel electrophoresis, blotted to nylon membranes, and analysed by hybridization with the ^32^P-labelled probe Or. Signal was acquired in a Fuji FLA5100 with the ImageGauge analysis program.

### Western blot

Yeast protein extracts to analyse Rad53 phosphorylation and Rfa1/Rfa1-MN expression were prepared using the TCA protocol [132]. Protein samples were resolved by 8% SDS-PAGE, probed with antibodies against Rad53 (Abcam, ab104232), Rfa1 (Abcam, ab221198) or Pgk1 (Invitrogen, 22C5D8) and detected with a peroxidase-conjugate antibody. The immunoluminescent signal was generated with either the WesternBright ECL (Advansta) or the Clarity Western ECL Substrate (BioRad) kit, acquired in a ChemiDoc MP image system and quantified with the Image Lab software (Biorad).

## Supporting information

S1 Fig Characterization of *RFA1-MN* cells.
**(A)** ChEC analysis of exponentially growing cells expressing Rfa1-MN incubated in the absence or presence of 0.005% MMS for 2 h. Total DNA from cells permeabilized and treated with 2 mM CaCl_2_ for different times is shown (left). Addition of Ca^2+^ is required for detection of Rfa1-MN-digested DNA, as determined by running total DNA of wild-type and *RFA1-MN* cells growing in the absence or presence of 0.005% MMS for 2 h (right). **(B)** Ionizing radiation and zeocin sensitivity of *RFA1-MN* cells, as determined by spotting 10-fold serial dilutions of the same number of mid-log growing cells. Wild-type and *rad52∆* cells were included as control. **(C)** Budding index and doubling time of wild-type and *RFA1-MN* cells. The mean and standard deviation of three (budding index) and two (doubling time) independent experiments are shown. **(D)** Effect of the *RFA1-MN* chimera in the viability of wild-type and *rad52∆* cells transformed with the *URA3*-based plasmid pMDL5 expressing Rad52 from the *GAL1* promoter in the indicated media, as determined by spotting 10-fold serial dilutions of the same number of mid-log growing cells. The lethality of the *RFA1-MN rad52∆* strain was rescued with the *URA3*-based plasmid pDML5, which expresses *RAD52* from the galactose-inducible *GAL1* promoter. This strain is able to grow, even though slowly, under glucose-repressing conditions; however, this is due to basal expression from the *GAL1* promoter, as indicated by the lack of growth in the presence of fluoroorotic acid (FOA) where only Ura^-^ cells are able to grow. **(E)** Effect of HU and calcium in the viability of *RFA1-MN* cells. The *rad52∆* strain was included to show the requirement of HR for the repair of HU-induced DNA lesions. The analyses were repeated at least twice with similar results.
(EPS)

S2 Fig HR and chromatin assembly requirements for *RFA1-MN* cell viability in the absence and presence of calcium.
**(A, C, and D)** Effect of the indicated mutations in the growth of *RFA1-MN* cells as determined by spotting 10-fold serial dilutions of the same number of mid-log growing cells onto SMM medium without or with the indicated concentrations of CaCl_2_. **(B)** DSB sensitivity of *rtt105∆* cells to HO-induced DSBs, as determined by spotting 10-fold serial dilutions of the same number of mid-log growing cells. Cells were transformed with pGAL-HO and grown in glucose (*GAL1p* repression) and galactose-containing medium (*GAL1p* activation). Wild-type and *rad52∆* cells were included as control. The analyses were repeated at least twice with similar results. Mutants scored in the SGA screening are shown in bold.
(EPS)

S3 Fig Checkpoint activation facilitates the repair of broken forks.
**(A)** Additive effect of *dun1∆* and HU in the growth of *RFA1-MN* cells as determined by spotting 10-fold serial dilutions of the same number of mid-log growing cells onto SMM medium without or with the indicated concentrations of CaCl_2_ and HU. **(B)** Cell cycle progression and budding index of wild-type cells synchronised in G1 with α-factor and released into S phase in the presence of 400mM CaCl_2_. The mean and standard deviation of three independent experiments are shown. **(C)** Rad53 activation in wild-type cells treated or not with 0.005% MMS for 2 hours in the absence and presence of 400mM CaCl_2_.
(EPS)

S4 Fig Additional genetic requirements for *RFA1-MN* cell viability in the absence and presence of calcium.
**(A–D)** Effect of the indicated mutations in the growth of *RFA1-MN* cells as determined by spotting 10-fold serial dilutions of the same number of mid-log growing cells onto SMM medium without or with the indicated concentrations of CaCl_2_. The analyses were repeated at least twice with similar results. Mutants scored in the SGA screening are shown in bold.
(EPS)

S5 Fig Raw data for figure panels.
Original blots for the indicated figure panels are shown.
(EPS)

S1 Table *Saccharomyces cerevisiae* genes studied in the SGA screening.
Genes analyzed in the customized library of null mutants are shown.
(XLSX)

S2 Table Positive hits from the SGA screening.
The name of the positive hits, their PCR validation and the effect of the null mutant on *RFA1-MN* viability are indicated.
(XLSX)

S3 Table *Saccharomyces cerevisiae* strains used in this study.
Strains, genotypes and references are indicated.
(DOCX)

S4 Table Raw data for figure plots.
Raw values to build budding index and doubling time plots are shown.
(XLSX)

## References

[1] Gaillard H, García-Muse T, Aguilera A. Replication stress and cancer. Nat Rev Cancer. 2015;15(5):276–89. doi: 10.1038/nrc3916 25907220

[2] Berti M, Cortez D, Lopes M. The plasticity of DNA replication forks in response to clinically relevant genotoxic stress. Nat Rev Mol Cell Biol. 2020;21(10):633–51. doi: 10.1038/s41580-020-0257-5 32612242

[3] Prado F. Homologous recombination: to fork and beyond. Genes (Basel). 2018;9(12):603. doi: 10.3390/genes9120603 30518053 PMC6316604

[4] Khatib JB, Nicolae CM, Moldovan G-L. Role of translesion DNA synthesis in the metabolism of replication-associated nascent strand gaps. J Mol Biol. 2024;436(1):168275. doi: 10.1016/j.jmb.2023.168275 37714300 PMC10842951

[5] Galanti L, Pfander B. Right time, right place-DNA damage and DNA replication checkpoints collectively safeguard S phase. EMBO J. 2018;37(21):e100681. doi: 10.15252/embj.2018100681 30287420 PMC6213279

[6] Chapman JR, Taylor MRG, Boulton SJ. Playing the end game: DNA double-strand break repair pathway choice. Mol Cell. 2012;47(4):497–510. doi: 10.1016/j.molcel.2012.07.029 22920291

[7] Scully R, Panday A, Elango R, Willis NA. DNA double-strand break repair-pathway choice in somatic mammalian cells. Nat Rev Mol Cell Biol. 2019;20(11):698–714. doi: 10.1038/s41580-019-0152-0 31263220 PMC7315405

[8] Pâques F, Haber JE. Multiple pathways of recombination induced by double-strand breaks in *Saccharomyces cerevisiae*. Microbiol Mol Biol Rev. 1999;63(2):349–404. doi: 10.1128/MMBR.63.2.349-404.1999 10357855 PMC98970

[9] Waterman DP, Haber JE, Smolka MB. Checkpoint responses to DNA double-strand breaks. Annu Rev Biochem. 2020;89:103–33. doi: 10.1146/annurev-biochem-011520-104722 32176524 PMC7311309

[10] Liu L, Malkova A. Break-induced replication: unraveling each step. Trends Genet. 2022;38(7):752–65. doi: 10.1016/j.tig.2022.03.011 35459559 PMC9197877

[11] Wilson MA, Kwon Y, Xu Y, Chung W-H, Chi P, Niu H, et al. Pif1 helicase and Polδ promote recombination-coupled DNA synthesis via bubble migration. Nature. 2013;502(7471):393–6. doi: 10.1038/nature12585 24025768 PMC3915060

[12] Deem A, Keszthelyi A, Blackgrove T, Vayl A, Coffey B, Mathur R, et al. Break-induced replication is highly inaccurate. PLoS Biol. 2011;9(2):e1000594. doi: 10.1371/journal.pbio.1000594 21347245 PMC3039667

[13] Smith CE, Llorente B, Symington LS. Template switching during break-induced replication. Nature. 2007;447(7140):102–5. doi: 10.1038/nature05723 17410126

[14] Malkova A, Ivanov EL, Haber JE. Double-strand break repair in the absence of RAD51 in yeast: a possible role for break-induced DNA replication. Proc Natl Acad Sci U S A. 1996;93(14):7131–6. doi: 10.1073/pnas.93.14.7131 8692957 PMC38948

[15] Clemente-Ruiz M, Prado F. Chromatin assembly controls replication fork stability. EMBO Rep. 2009;10(7):790–6. doi: 10.1038/embor.2009.67 19465889 PMC2727422

[16] González-Barrera S, Cortés-Ledesma F, Wellinger RE, Aguilera A. Equal sister chromatid exchange is a major mechanism of double-strand break repair in yeast. Mol Cell. 2003;11(6):1661–71. doi: 10.1016/s1097-2765(03)00183-7 12820977

[17] Mayle R, Campbell IM, Beck CR, Yu Y, Wilson M, Shaw CA, et al. DNA REPAIR. Mus81 and converging forks limit the mutagenicity of replication fork breakage. Science. 2015;349(6249):742–7. doi: 10.1126/science.aaa8391 26273056 PMC4782627

[18] Strumberg D, Pilon AA, Smith M, Hickey R, Malkas L, Pommier Y. Conversion of topoisomerase I cleavage complexes on the leading strand of ribosomal DNA into 5’-phosphorylated DNA double-strand breaks by replication runoff. Mol Cell Biol. 2000;20(11):3977–87. doi: 10.1128/MCB.20.11.3977-3987.2000 10805740 PMC85758

[19] Vrtis KB, Dewar JM, Chistol G, Wu RA, Graham TGW, Walter JC. Single-strand DNA breaks cause replisome disassembly. Mol Cell. 2021;81(6):1309–18.e6. doi: 10.1016/j.molcel.2020.12.039 33484638 PMC7979477

[20] Pavani R, Tripathi V, Vrtis KB, Zong D, Chari R, Callen E, et al. Structure and repair of replication-coupled DNA breaks. Science. 2024;385(6710):eado3867. doi: 10.1126/science.ado3867 38900911 PMC11620331

[21] Kimble MT, Sane A, Reid RJD, Johnson MJ, Rothstein R, Symington LS. Repair of replication-dependent double-strand breaks differs between the leading and lagging strands. Mol Cell. 2025;85(1):61–77.e6. doi: 10.1016/j.molcel.2024.10.032 39631395 PMC11698654

[22] Elango R, Nilavar NM, Li AG, Nguyen D, Rass E, Duffey EE, et al. Two-ended recombination at a Flp-nickase-broken replication fork. Mol Cell. 2025;85(1):78–90.e3. doi: 10.1016/j.molcel.2024.11.006 39631396 PMC11733529

[23] XuY, MorrowCA, LaksirY, HoltOM, TaylorK, TsiappourdhiC, et al. DNA nicks in both leading and lagging strand templates can trigger break-induced replication. Mol Cell. 2025;85(1):91-106.e5. doi: 10.1016/j.molcel.2024.10.026 39561776

[24] Macheret M, Halazonetis TD. DNA replication stress as a hallmark of cancer. Annu Rev Pathol. 2015;10:425–48. doi: 10.1146/annurev-pathol-012414-040424 25621662

[25] Hashimoto Y, Puddu F, Costanzo V. RAD51- and MRE11-dependent reassembly of uncoupled CMG helicase complex at collapsed replication forks. Nat Struct Mol Biol. 2011;19(1):17–24. doi: 10.1038/nsmb.2177 22139015 PMC4306020

[26] Schmid M, Durussel T, Laemmli UK. ChIC and ChEC; genomic mapping of chromatin proteins. Mol Cell. 2004;16(1):147–57. doi: 10.1016/j.molcel.2004.09.007 15469830

[27] González-Prieto R, Muñoz-Cabello AM, Cabello-Lobato MJ, Prado F. Rad51 replication fork recruitment is required for DNA damage tolerance. EMBO J. 2013;32(9):1307–21. doi: 10.1038/emboj.2013.73 23563117 PMC3642682

[28] González-Prieto R, Cabello-Lobato MJ, Prado F. In Vivo Binding of Recombination Proteins to Non-DSB DNA Lesions and to Replication Forks. Methods Mol Biol. 2021;2153:447–58. doi: 10.1007/978-1-0716-0644-5_31 32840798

[29] Cano-Linares MI, Yáñez-Vilches A, García-Rodríguez N, Barrientos-Moreno M, González-Prieto R, San-Segundo P, et al. Non-recombinogenic roles for Rad52 in translesion synthesis during DNA damage tolerance. EMBO Rep. 2021;22(1):e50410. doi: 10.15252/embr.202050410 33289333 PMC7788459

[30] Cabello-Lobato MJ, González-Garrido C, Cano-Linares MI, Wong RP, Yáñez-Vílchez A, Morillo-Huesca M, et al. Physical interactions between MCM and Rad51 facilitate replication fork lesion bypass and ssDNA gap filling by non-recombinogenic functions. Cell Rep. 2021;36(4):109440. doi: 10.1016/j.celrep.2021.109440 34320356

[31] González-Garrido C, Prado F. Parental histone distribution and location of the replication obstacle at nascent strands control homologous recombination. Cell Rep. 2023;42(3):112174. doi: 10.1016/j.celrep.2023.112174 36862554

[32] Hung S-H, Wong RP, Ulrich HD, Kao C-F. Monoubiquitylation of histone H2B contributes to the bypass of DNA damage during and after DNA replication. Proc Natl Acad Sci U S A. 2017;114(11):E2205–14. doi: 10.1073/pnas.1612633114 28246327 PMC5358361

[33] Litwin I, Bakowski T, Szakal B, Pilarczyk E, Maciaszczyk-Dziubinska E, Branzei D, et al. Error-free DNA damage tolerance pathway is facilitated by the Irc5 translocase through cohesin. EMBO J. 2018;37(18):e98732. doi: 10.15252/embj.201798732 30111537 PMC6138436

[34] Dueva R, Iliakis G. Replication protein A: a multifunctional protein with roles in DNA replication, repair and beyond. NAR Cancer. 2020;2(3):zcaa022. doi: 10.1093/narcan/zcaa022 34316690 PMC8210275

[35] Zhang S, Wang X, Zhao H, Shi J, Chen X. New insights into the mechanism of RPA in preserving genome stability. GENOME INSTAB DIS. 2022;3(5):255–66. doi: 10.1007/s42764-022-00085-y

[36] Sikorski TW, Ficarro SB, Holik J, Kim T, Rando OJ, Marto JA, et al. Sub1 and RPA associate with RNA polymerase II at different stages of transcription. Mol Cell. 2011;44(3):397–409. doi: 10.1016/j.molcel.2011.09.013 22055186 PMC3227220

[37] Li S, Xu Z, Xu J, Zuo L, Yu C, Zheng P, et al. Rtt105 functions as a chaperone for replication protein A to preserve genome stability. EMBO J. 2018;37(17):e99154. doi: 10.15252/embj.201899154 30065069 PMC6120668

[38] Li L, Wang J, Yang Z, Zhao Y, Jiang H, Jiang L, et al. Metabolic remodeling maintains a reducing environment for rapid activation of the yeast DNA replication checkpoint. EMBO J. 2022;41(4):e108290. doi: 10.15252/embj.2021108290 35028974 PMC8844976

[39] Wong RP, García-Rodríguez N, Zilio N, Hanulová M, Ulrich HD. Processing of DNA Polymerase-Blocking Lesions during Genome Replication Is Spatially and Temporally Segregated from Replication Forks. Mol Cell. 2020;77(1):3–16.e4. doi: 10.1016/j.molcel.2019.09.015 31607544

[40] Kramer KM, Brock JA, Bloom K, Moore JK, Haber JE. Two different types of double-strand breaks in *Saccharomyces cerevisiae* are repaired by similar RAD52-independent, nonhomologous recombination events. Mol Cell Biol. 1994;14(2):1293–301. doi: 10.1128/mcb.14.2.1293-1301.1994 8289808 PMC358484

[41] Wyatt HDM, West SC. Holliday junction resolvases. Cold Spring Harb Perspect Biol. 2014;6(9):a023192. doi: 10.1101/cshperspect.a023192 25183833 PMC4142969

[42] Kuzmin E, Costanzo M, Andrews B, Boone C. Synthetic Genetic Array Analysis. Cold Spring Harb Protoc. 2016;2016(4):pdb.prot088807. doi: 10.1101/pdb.prot088807 27037072

[43] Kellermayer R, Aiello DP, Miseta A, Bedwell DM. Extracellular Ca(2+) sensing contributes to excess Ca(2+) accumulation and vacuolar fragmentation in a *pmr1Delta* mutant of *S. cerevisiae*. J Cell Sci. 2003;116(Pt 8):1637–46. doi: 10.1242/jcs.00372 12640047

[44] San Filippo J, Sung P, Klein H. Mechanism of eukaryotic homologous recombination. Annu Rev Biochem. 2008;77:229–57. doi: 10.1146/annurev.biochem.77.061306.125255 18275380

[45] Prado F, Cortés-Ledesma F, Huertas P, Aguilera A. Mitotic recombination in *Saccharomyces cerevisiae*. Curr Genet. 2003;42(4):185–98. doi: 10.1007/s00294-002-0346-3 12589470

[46] Wang X, Dong Y, Zhao X, Li J, Lee J, Yan Z, et al. Rtt105 promotes high-fidelity DNA replication and repair by regulating the single-stranded DNA-binding factor RPA. Proc Natl Acad Sci U S A. 2021;118(25):e2106393118. doi: 10.1073/pnas.2106393118 34140406 PMC8237652

[47] Ruff P, Donnianni RA, Glancy E, Oh J, Symington LS. RPA stabilization of single-stranded DNA is critical for break-induced replication. Cell Rep. 2016;17(12):3359–68. doi: 10.1016/j.celrep.2016.12.003 28009302 PMC5218512

[48] Symington LS. Mechanism and regulation of DNA end resection in eukaryotes. Crit Rev Biochem Mol Biol. 2016;51(3):195–212. doi: 10.3109/10409238.2016.1172552 27098756 PMC4957645

[49] Ira G, Malkova A, Liberi G, Foiani M, Haber JE. Srs2 and Sgs1-Top3 suppress crossovers during double-strand break repair in yeast. Cell. 2003;115(4):401–11. doi: 10.1016/s0092-8674(03)00886-9 14622595 PMC4493758

[50] Ip SCY, Rass U, Blanco MG, Flynn HR, Skehel JM, West SC. Identification of Holliday junction resolvases from humans and yeast. Nature. 2008;456(7220):357–61. doi: 10.1038/nature07470 19020614

[51] Godin S, Wier A, Kabbinavar F, Bratton-Palmer DS, Ghodke H, Van Houten B, et al. The Shu complex interacts with Rad51 through the Rad51 paralogues Rad55-Rad57 to mediate error-free recombination. Nucleic Acids Res. 2013;41(8):4525–34. doi: 10.1093/nar/gkt138 23460207 PMC3632125

[52] Godin SK, Zhang Z, Herken BW, Westmoreland JW, Lee AG, Mihalevic MJ, et al. The Shu complex promotes error-free tolerance of alkylation-induced base excision repair products. Nucleic Acids Res. 2016;44(17):8199–215. doi: 10.1093/nar/gkw535 27298254 PMC5041462

[53] Shor E, Weinstein J, Rothstein R. A genetic screen for top3 suppressors in *Saccharomyces cerevisiae* identifies *SHU1*, *SHU2*, *PSY3* and *CSM2*: four genes involved in error-free DNA repair. Genetics. 2005;169(3):1275–89. doi: 10.1534/genetics.104.036764 15654096 PMC1449555

[54] Collins SR, Miller KM, Maas NL, Roguev A, Fillingham J, Chu CS, et al. Functional dissection of protein complexes involved in yeast chromosome biology using a genetic interaction map. Nature. 2007;446(7137):806–10. doi: 10.1038/nature05649 17314980

[55] Pan X, Ye P, Yuan DS, Wang X, Bader JS, Boeke JD. A DNA integrity network in the yeast *Saccharomyces cerevisiae*. Cell. 2006;124(5):1069–81. doi: 10.1016/j.cell.2005.12.036 16487579

[56] Driscoll R, Hudson A, Jackson SP. Yeast Rtt109 promotes genome stability by acetylating histone H3 on lysine 56. Science. 2007;315(5812):649–52. doi: 10.1126/science.1135862 17272722 PMC3334813

[57] Han J, Zhou H, Horazdovsky B, Zhang K, Xu R-M, Zhang Z. Rtt109 acetylates histone H3 lysine 56 and functions in DNA replication. Science. 2007;315(5812):653–5. doi: 10.1126/science.1133234 17272723

[58] Li Q, Zhou H, Wurtele H, Davies B, Horazdovsky B, Verreault A, et al. Acetylation of histone H3 lysine 56 regulates replication-coupled nucleosome assembly. Cell. 2008;134(2):244–55. doi: 10.1016/j.cell.2008.06.018 18662540 PMC2597342

[59] Han J, Zhang H, Zhang H, Wang Z, Zhou H, Zhang Z. A Cul4 E3 ubiquitin ligase regulates histone hand-off during nucleosome assembly. Cell. 2013;155(4):817–29. doi: 10.1016/j.cell.2013.10.014 24209620 PMC3994564

[60] Buser R, Kellner V, Melnik A, Wilson-Zbinden C, Schellhaas R, Kastner L, et al. The Replisome-Coupled E3 Ubiquitin Ligase Rtt101Mms22 Counteracts Mrc1 Function to Tolerate Genotoxic Stress. PLoS Genet. 2016;12(2):e1005843. doi: 10.1371/journal.pgen.1005843 26849847 PMC4743919

[61] Prado F, Cortés-Ledesma F, Aguilera A. The absence of the yeast chromatin assembly factor Asf1 increases genomic instability and sister chromatid exchange. EMBO Rep. 2004;5(5):497–502. doi: 10.1038/sj.embor.7400128 15071494 PMC1299049

[62] Luke B, Versini G, Jaquenoud M, Zaidi IW, Kurz T, Pintard L, et al. The cullin Rtt101p promotes replication fork progression through damaged DNA and natural pause sites. Curr Biol. 2006;16(8):786–92. doi: 10.1016/j.cub.2006.02.071 16631586

[63] Duro E, Vaisica JA, Brown GW, Rouse J. Budding yeast Mms22 and Mms1 regulate homologous recombination induced by replisome blockage. DNA Repair (Amst). 2008;7(5):811–8. doi: 10.1016/j.dnarep.2008.01.007 18321796

[64] Wurtele H, Kaiser GS, Bacal J, St-Hilaire E, Lee E-H, Tsao S, et al. Histone H3 lysine 56 acetylation and the response to DNA replication fork damage. Mol Cell Biol. 2012;32(1):154–72. doi: 10.1128/MCB.05415-11 22025679 PMC3255698

[65] Clemente-Ruiz M, González-Prieto R, Prado F. Histone H3K56 acetylation, CAF1, and Rtt106 coordinate nucleosome assembly and stability of advancing replication forks. PLoS Genet. 2011;7(11):e1002376. doi: 10.1371/journal.pgen.1002376 22102830 PMC3213180

[66] Luciano P, Dehé P-M, Audebert S, Géli V, Corda Y. Replisome function during replicative stress is modulated by histone h3 lysine 56 acetylation through Ctf4. Genetics. 2015;199(4):1047–63. doi: 10.1534/genetics.114.173856 25697176 PMC4391565

[67] Erkmann JA, Kaufman PD. A negatively charged residue in place of histone H3K56 supports chromatin assembly factor association but not genotoxic stress resistance. DNA Repair (Amst). 2009;8(12):1371–9. doi: 10.1016/j.dnarep.2009.09.004 19796999 PMC2787813

[68] Yang J, Zhang X, Feng J, Leng H, Li S, Xiao J, et al. The Histone Chaperone FACT Contributes to DNA Replication-Coupled Nucleosome Assembly. Cell Rep. 2016;14(5):1128–41. doi: 10.1016/j.celrep.2015.12.096 26804921

[69] Gan H, Serra-Cardona A, Hua X, Zhou H, Labib K, Yu C, et al. The Mcm2-Ctf4-Polα Axis Facilitates Parental Histone H3-H4 Transfer to Lagging Strands. Mol Cell. 2018;72(1):140–51.e3. doi: 10.1016/j.molcel.2018.09.001 30244834 PMC6193272

[70] Li Z, Hua X, Serra-Cardona A, Xu X, Gan S, Zhou H, et al. DNA polymerase α interacts with H3-H4 and facilitates the transfer of parental histones to lagging strands. Sci Adv. 2020;6(35):eabb5820. doi: 10.1126/sciadv.abb5820 32923642 PMC7449674

[71] Yu C, Gan H, Serra-Cardona A, Zhang L, Gan S, Sharma S, et al. A mechanism for preventing asymmetric histone segregation onto replicating DNA strands. Science. 2018;361(6409):1386–9. doi: 10.1126/science.aat8849 30115745 PMC6597248

[72] Maas NL, Miller KM, DeFazio LG, Toczyski DP. Cell cycle and checkpoint regulation of histone H3 K56 acetylation by Hst3 and Hst4. Mol Cell. 2006;23(1):109–19. doi: 10.1016/j.molcel.2006.06.006 16818235

[73] Celic I, Masumoto H, Griffith WP, Meluh P, Cotter RJ, Boeke JD, et al. The sirtuins hst3 and Hst4p preserve genome integrity by controlling histone h3 lysine 56 deacetylation. Curr Biol. 2006;16(13):1280–9. doi: 10.1016/j.cub.2006.06.023 16815704

[74] Che J, Smith S, Kim YJ, Shim EY, Myung K, Lee SE. Hyper-Acetylation of Histone H3K56 Limits Break-Induced Replication by Inhibiting Extensive Repair Synthesis. PLoS Genet. 2015;11(2):e1004990. doi: 10.1371/journal.pgen.1004990 25705897 PMC4338291

[75] Roberts TM, Zaidi IW, Vaisica JA, Peter M, Brown GW. Regulation of rtt107 recruitment to stalled DNA replication forks by the cullin rtt101 and the rtt109 acetyltransferase. Mol Biol Cell. 2008;19(1):171–80. doi: 10.1091/mbc.e07-09-0961 17978089 PMC2174167

[76] Hang LE, Peng J, Tan W, Szakal B, Menolfi D, Sheng Z, et al. Rtt107 Is a Multi-functional Scaffold Supporting Replication Progression with Partner SUMO and Ubiquitin Ligases. Mol Cell. 2015;60(2):268–79. doi: 10.1016/j.molcel.2015.08.023 26439300 PMC4609303

[77] Princz LN, Wild P, Bittmann J, Aguado FJ, Blanco MG, Matos J, et al. Dbf4-dependent kinase and the Rtt107 scaffold promote Mus81-Mms4 resolvase activation during mitosis. EMBO J. 2017;36(5):664–78. doi: 10.15252/embj.201694831 28096179 PMC5331752

[78] Bermúdez-López M, Villoria MT, Esteras M, Jarmuz A, Torres-Rosell J, Clemente-Blanco A, et al. Sgs1’s roles in DNA end resection, HJ dissolution, and crossover suppression require a two-step SUMO regulation dependent on Smc5/6. Genes Dev. 2016;30(11):1339–56. doi: 10.1101/gad.278275.116 27298337 PMC4911932

[79] Branzei D, Vanoli F, Foiani M. SUMOylation regulates Rad18-mediated template switch. Nature. 2008;456(7224):915–20. doi: 10.1038/nature07587 19092928

[80] Hoege C, Pfander B, Moldovan G-L, Pyrowolakis G, Jentsch S. RAD6-dependent DNA repair is linked to modification of PCNA by ubiquitin and SUMO. Nature. 2002;419(6903):135–41. doi: 10.1038/nature00991 12226657

[81] Kang M-J, Lee C-H, Kang Y-H, Cho I-T, Nguyen TA, Seo Y-S. Genetic and functional interactions between Mus81-Mms4 and Rad27. Nucleic Acids Res. 2010;38(21):7611–25. doi: 10.1093/nar/gkq651 20660481 PMC2995070

[82] Harrison JC, Haber JE. Surviving the breakup: the DNA damage checkpoint. Annu Rev Genet. 2006;40:209–35. doi: 10.1146/annurev.genet.40.051206.105231 16805667

[83] Jossen R, Bermejo R. The DNA damage checkpoint response to replication stress: A Game of Forks. Front Genet. 2013;4:26. doi: 10.3389/fgene.2013.00026 23493417 PMC3595514

[84] Ferrari M, Dibitetto D, De Gregorio G, Eapen VV, Rawal CC, Lazzaro F, et al. Functional interplay between the 53BP1-ortholog Rad9 and the Mre11 complex regulates resection, end-tethering and repair of a double-strand break. PLoS Genet. 2015;11(1):e1004928. doi: 10.1371/journal.pgen.1004928 25569305 PMC4287487

[85] Burgess RJ, Han J, Zhang Z. The Ddc1-Mec3-Rad17 sliding clamp regulates histone-histone chaperone interactions and DNA replication-coupled nucleosome assembly in budding yeast. J Biol Chem. 2014;289(15):10518–29. doi: 10.1074/jbc.M114.552463 24573675 PMC4036173

[86] Karras GI, Fumasoni M, Sienski G, Vanoli F, Branzei D, Jentsch S. Noncanonical role of the 9-1-1 clamp in the error-free DNA damage tolerance pathway. Mol Cell. 2013;49(3):536–46. doi: 10.1016/j.molcel.2012.11.016 23260657

[87] Lou H, Komata M, Katou Y, Guan Z, Reis CC, Budd M, et al. Mrc1 and DNA polymerase epsilon function together in linking DNA replication and the S phase checkpoint. Mol Cell. 2008;32(1):106–17. doi: 10.1016/j.molcel.2008.08.020 18851837 PMC2699584

[88] Katou Y, Kanoh Y, Bando M, Noguchi H, Tanaka H, Ashikari T, et al. S-phase checkpoint proteins Tof1 and Mrc1 form a stable replication-pausing complex. Nature. 2003;424(6952):1078–83. doi: 10.1038/nature01900 12944972

[89] Calzada A, Hodgson B, Kanemaki M, Bueno A, Labib K. Molecular anatomy and regulation of a stable replisome at a paused eukaryotic DNA replication fork. Genes Dev. 2005;19(16):1905–19. doi: 10.1101/gad.337205 16103218 PMC1186190

[90] Srinivasan M, Fumasoni M, Petela NJ, Murray A, Nasmyth KA. Cohesion is established during DNA replication utilising chromosome associated cohesin rings as well as those loaded de novo onto nascent DNAs. Elife. 2020;9:e56611. doi: 10.7554/eLife.56611 32515737 PMC7282809

[91] Tourrière H, Versini G, Cordón-Preciado V, Alabert C, Pasero P. Mrc1 and Tof1 promote replication fork progression and recovery independently of Rad53. Mol Cell. 2005;19(5):699–706. doi: 10.1016/j.molcel.2005.07.028 16137625

[92] Crabbé L, Thomas A, Pantesco V, De Vos J, Pasero P, Lengronne A. Analysis of replication profiles reveals key role of RFC-Ctf18 in yeast replication stress response. Nat Struct Mol Biol. 2010;17(11):1391–7. doi: 10.1038/nsmb.1932 20972444

[93] Lengronne A, McIntyre J, Katou Y, Kanoh Y, Hopfner K-P, Shirahige K, et al. Establishment of sister chromatid cohesion at the *S. cerevisiae* replication fork. Mol Cell. 2006;23(6):787–99. doi: 10.1016/j.molcel.2006.08.018 16962805

[94] Chang M, Bellaoui M, Boone C, Brown GW. A genome-wide screen for methyl methanesulfonate-sensitive mutants reveals genes required for S phase progression in the presence of DNA damage. Proc Natl Acad Sci U S A. 2002;99(26):16934–9. doi: 10.1073/pnas.262669299 12482937 PMC139247

[95] Hoch NC, Chen ES-W, Buckland R, Wang S-C, Fazio A, Hammet A, et al. Molecular basis of the essential s phase function of the rad53 checkpoint kinase. Mol Cell Biol. 2013;33(16):3202–13. doi: 10.1128/MCB.00474-13 23754745 PMC3753913

[96] Samora CP, Saksouk J, Goswami P, Wade BO, Singleton MR, Bates PA, et al. Ctf4 Links DNA Replication with Sister Chromatid Cohesion Establishment by Recruiting the Chl1 Helicase to the Replisome. Mol Cell. 2016;63(3):371–84. doi: 10.1016/j.molcel.2016.05.036 27397686 PMC4980427

[97] Gambus A, van Deursen F, Polychronopoulos D, Foltman M, Jones RC, Edmondson RD, et al. A key role for Ctf4 in coupling the MCM2-7 helicase to DNA polymerase alpha within the eukaryotic replisome. EMBO J. 2009;28(19):2992–3004. doi: 10.1038/emboj.2009.226 19661920 PMC2760104

[98] Morohashi H, Maculins T, Labib K. The amino-terminal TPR domain of Dia2 tethers SCF(Dia2) to the replisome progression complex. Curr Biol. 2009;19(22):1943–9. doi: 10.1016/j.cub.2009.09.062 19913425

[99] Fumasoni M, Zwicky K, Vanoli F, Lopes M, Branzei D. Error-free DNA damage tolerance and sister chromatid proximity during DNA replication rely on the Polα/Primase/Ctf4 Complex. Mol Cell. 2015;57(5):812–23. doi: 10.1016/j.molcel.2014.12.038 25661486 PMC4352764

[100] Kueng S, Hegemann B, Peters BH, Lipp JJ, Schleiffer A, Mechtler K, et al. Wapl controls the dynamic association of cohesin with chromatin. Cell. 2006;127(5):955–67. doi: 10.1016/j.cell.2006.09.040 17113138

[101] Eng T, Guacci V, Koshland D. Interallelic complementation provides functional evidence for cohesin-cohesin interactions on DNA. Mol Biol Cell. 2015;26(23):4224–35. doi: 10.1091/mbc.E15-06-0331 26378250 PMC4642856

[102] Haering CH, Schoffnegger D, Nishino T, Helmhart W, Nasmyth K, Löwe J. Structure and stability of cohesin’s Smc1-kleisin interaction. Mol Cell. 2004;15(6):951–64. doi: 10.1016/j.molcel.2004.08.030 15383284

[103] Barberis M. Sic1 as a timer of Clb cyclin waves in the yeast cell cycle--design principle of not just an inhibitor. FEBS J. 2012;279(18):3386–410. doi: 10.1111/j.1742-4658.2012.08542.x 22356687

[104] Ayuda-Durán P, Devesa F, Gomes F, Sequeira-Mendes J, Avila-Zarza C, Gómez M, et al. The CDK regulators Cdh1 and Sic1 promote efficient usage of DNA replication origins to prevent chromosomal instability at a chromosome arm. Nucleic Acids Res. 2014;42(11):7057–68. doi: 10.1093/nar/gku313 24753426 PMC4066753

[105] Lengronne A, Schwob E. The yeast CDK inhibitor Sic1 prevents genomic instability by promoting replication origin licensing in late G(1). Mol Cell. 2002;9(5):1067–78. doi: 10.1016/s1097-2765(02)00513-0 12049742

[106] Costanzo M, Nishikawa JL, Tang X, Millman JS, Schub O, Breitkreuz K, et al. CDK activity antagonizes Whi5, an inhibitor of G1/S transcription in yeast. Cell. 2004;117(7):899–913. doi: 10.1016/j.cell.2004.05.024 15210111

[107] Jakobsen KP, Nielsen KO, Løvschal KV, Rødgaard M, Andersen AH, Bjergbæk L. Minimal Resection Takes Place during Break-Induced Replication Repair of Collapsed Replication Forks and Is Controlled by Strand Invasion. Cell Rep. 2019;26(4):836–44.e3. doi: 10.1016/j.celrep.2018.12.108 30673606

[108] Tittel-Elmer M, Alabert C, Pasero P, Cobb JA. The MRX complex stabilizes the replisome independently of the S phase checkpoint during replication stress. EMBO J. 2009;28(8):1142–56. doi: 10.1038/emboj.2009.60 19279665 PMC2683708

[109] Zhu M, Zhao H, Limbo O, Russell P. Mre11 complex links sister chromatids to promote repair of a collapsed replication fork. Proc Natl Acad Sci U S A. 2018;115(35):8793–8. doi: 10.1073/pnas.1808189115 30104346 PMC6126713

[110] Fasching CL, Cejka P, Kowalczykowski SC, Heyer W-D. Top3-Rmi1 dissolve Rad51-mediated D loops by a topoisomerase-based mechanism. Mol Cell. 2015;57(4):595–606. doi: 10.1016/j.molcel.2015.01.022 25699708 PMC4338411

[111] Gaines WA, Godin SK, Kabbinavar FF, Rao T, VanDemark AP, Sung P, et al. Promotion of presynaptic filament assembly by the ensemble of *S. cerevisiae* Rad51 paralogues with Rad52. Nat Commun. 2015;6:7834. doi: 10.1038/ncomms8834 26215801 PMC4525180

[112] Schindler N, Tonn M, Kellner V, Fung JJ, Lockhart A, Vydzhak O, et al. Genetic requirements for repair of lesions caused by single genomic ribonucleotides in S phase. Nat Commun. 2023;14(1):1227. doi: 10.1038/s41467-023-36866-6 36869098 PMC9984532

[113] Villa F, Simon AC, Ortiz Bazan MA, Kilkenny ML, Wirthensohn D, Wightman M, et al. Ctf4 Is a Hub in the Eukaryotic Replisome that Links Multiple CIP-Box Proteins to the CMG Helicase. Mol Cell. 2016;63(3):385–96. doi: 10.1016/j.molcel.2016.06.009 27397685 PMC4980431

[114] Tittel-Elmer M, Lengronne A, Davidson MB, Bacal J, François P, Hohl M, et al. Cohesin association to replication sites depends on rad50 and promotes fork restart. Mol Cell. 2012;48(1):98–108. doi: 10.1016/j.molcel.2012.07.004 22885006 PMC3904740

[115] Ström L, Lindroos HB, Shirahige K, Sjögren C. Postreplicative recruitment of cohesin to double-strand breaks is required for DNA repair. Mol Cell. 2004;16(6):1003–15. doi: 10.1016/j.molcel.2004.11.026 15610742

[116] Ayyagari R, Gomes XV, Gordenin DA, Burgers PMJ. Okazaki fragment maturation in yeast. I. Distribution of functions between FEN1 AND DNA2. J Biol Chem. 2003;278(3):1618–25. doi: 10.1074/jbc.M209801200 12424238

[117] Saini N, Ramakrishnan S, Elango R, Ayyar S, Zhang Y, Deem A, et al. Migrating bubble during break-induced replication drives conservative DNA synthesis. Nature. 2013;502(7471):389–92. doi: 10.1038/nature12584 24025772 PMC3804423

[118] Liu L, Yan Z, Osia BA, Twarowski J, Sun L, Kramara J, et al. Tracking break-induced replication shows that it stalls at roadblocks. Nature. 2021;590(7847):655–9. doi: 10.1038/s41586-020-03172-w 33473214 PMC8219245

[119] Simon AC, Zhou JC, Perera RL, van Deursen F, Evrin C, Ivanova ME, et al. A Ctf4 trimer couples the CMG helicase to DNA polymerase α in the eukaryotic replisome. Nature. 2014;510(7504):293–7. doi: 10.1038/nature13234 24805245 PMC4059944

[120] Sotiriou SK, Kamileri I, Lugli N, Evangelou K, Da-Ré C, Huber F, et al. Mammalian RAD52 Functions in Break-Induced Replication Repair of Collapsed DNA Replication Forks. Mol Cell. 2016;64(6):1127–34. doi: 10.1016/j.molcel.2016.10.038 27984746 PMC5179496

[121] Hills SA, Diffley JFX. DNA replication and oncogene-induced replicative stress. Curr Biol. 2014;24(10):R435-44. doi: 10.1016/j.cub.2014.04.012 24845676

[122] Ransom M, Dennehey BK, Tyler JK. Chaperoning histones during DNA replication and repair. Cell. 2010;140(2):183–95. doi: 10.1016/j.cell.2010.01.004 20141833 PMC3433953

[123] Miseta A, Fu L, Kellermayer R, Buckley J, Bedwell DM. The Golgi apparatus plays a significant role in the maintenance of Ca2+ homeostasis in the vps33Delta vacuolar biogenesis mutant of *Saccharomyces cerevisiae*. J Biol Chem. 1999;274(9):5939–47. doi: 10.1074/jbc.274.9.5939 10026219

[124] Cui J, Kaandorp JA, Ositelu OO, Beaudry V, Knight A, Nanfack YF, et al. Simulating calcium influx and free calcium concentrations in yeast. Cell Calcium. 2009;45(2):123–32. doi: 10.1016/j.ceca.2008.07.005 18783827 PMC3130064

[125] Debatisse M, Le Tallec B, Letessier A, Dutrillaux B, Brison O. Common fragile sites: mechanisms of instability revisited. Trends Genet. 2012;28(1):22–32. doi: 10.1016/j.tig.2011.10.003 22094264

[126] Gilson E, Géli V. How telomeres are replicated. Nat Rev Mol Cell Biol. 2007;8(10):825–38. doi: 10.1038/nrm2259 17885666

[127] Epum EA, Haber JE. DNA replication: the recombination connection. Trends Cell Biol. 2022;32(1):45–57. doi: 10.1016/j.tcb.2021.07.005 34384659 PMC8688190

[128] Longtine MS, Mckenzie III A, Demarini DJ, Shah NG, Wach A, Brachat A, et al. Additional modules for versatile and economical PCR-based gene deletion and modification in *Saccharomyces cerevisiae*. Yeast. 1998;14(10):953–61. doi: 10.1002/(sici)1097-0061(199807)14:10<953::aid-yea293>3.0.co;2-u 9717241

[129] Jensen RE, Herskowitz I. Cold Spring Harbor Symposium on Quantitative Biology. 1980. p. 97–104. 10.1101/sqb.1984.049.01.013 6397325

[130] Prado F, Aguilera A. Partial depletion of histone H4 increases homologous recombination-mediated genetic instability. Mol Cell Biol. 2005;25(4):1526–36. doi: 10.1128/MCB.25.4.1526-1536.2005 15684401 PMC548009

[131] Bernstein KA, Shor E, Sunjevaric I, Fumasoni M, Burgess RC, Foiani M, et al. Sgs1 function in the repair of DNA replication intermediates is separable from its role in homologous recombinational repair. EMBO J. 2009;28(7):915–25. doi: 10.1038/emboj.2009.28 19214189 PMC2670856

[132] Foiani M, Marini F, Gamba D, Lucchini G, Plevani P. The B subunit of the DNA polymerase alpha-primase complex in *Saccharomyces cerevisiae* executes an essential function at the initial stage of DNA replication. Mol Cell Biol. 1994;14(2):923–33. doi: 10.1128/mcb.14.2.923-933.1994 8289832 PMC358447

