# Supplementary material for: A Rfa1-MN–based system reveals new factors involved in the rescue of broken replication forks
Source: PLoS Genet. 2025 Apr 1;21(4):e1011405. doi: 10.1371/journal.pgen.1011405 (PMC11984746; doi:10.1371/journal.pgen.1011405)
Supplement: S3 Table — Strains, genotypes and references are indicated. (DOCX) [file pgen.1011405.s008.docx]

**Table S3. Strains used in this study**

| **Strain** | ***Genotype*** | **Source** |
| --- | --- | --- |
| BYRPAMN-SGA-1B | *MAT alfa LYS2 met15∆0 ura3∆0 leu2∆0 his3∆0 bar1ΔHyg can1∆::STE2pr Sp-his5 lyp∆1 RFA1-MN::NAT* | [1] |
| BYtrp1y7092-2A | *MAT alfa LYS2 met15∆0 ura3∆0 leu2∆0 his3∆0 bar1ΔHyg can1∆::STE2pr Sp-his5 lyp∆1 trp1∆NAT* | [1] |
| BY4741 | *MAT a LYS2 met15∆0 ura3∆0 leu2∆0 his3∆0* | Euroscarf |
| BYRfa1MNk | *MAT alfa MET15 lys2∆0 ura3∆0 leu2∆0 his3∆0 RFA1-MN::KAN* | This study |
| BYRfa1MNn | *MAT alfa MET15 lys2∆0 ura3∆0 leu2∆0 his3∆0 RFA1-MN::NAT* | This study |
| W303-1A | *MAT a leu2-3,112 trp1-1 ura3-1 ade2-1 his3-11,15 can1-100* | [2] |
| WRfa1MN | *MAT a leu2-3,112 trp1-1 ura3-1 ade2-1 his3-11,15 can1-100 RFA1-MN::KAN* | This study |
| BYrad52 | *MAT a met15∆0 lys2∆0 ura3∆0 leu2∆0 his3∆0 rad52ΔKAN* | Euroscarf |
| BYG2R52 | *MAT a met15∆0 lys2∆0 ura3∆0 leu2∆0 his3∆0 CLB2p::cRAD52::NAT* | [2] |
| BYRfa1MN G2R52 | *MAT a met15∆0 lys2∆0 ura3∆0 leu2∆0 his3∆0 CLB2p::cRAD52::NAT RFA1-MN::KAN* | This study |
| BYrad51 | *MAT a met15∆0 lys2∆0 ura3∆0 leu2∆0 his3∆0 rad51ΔKAN* | Euroscarf |
| ByRfa1r51-4D  ByRfa1r51-5B | *MAT α met15∆0 lys2∆0 ura3∆0 leu2∆0 his3∆0 bar1ΔHyg RFA1-MN::Kan* | This study |
| BYmre11 | *MAT a met15∆0 lys2∆0 ura3∆0 leu2∆0 his3∆0 mre11ΔKAN* | Euroscarf |
| BYRfa1MNm11-9A  BYRfa1MNm11-2C | *MAT a met15∆0 lys2∆0 ura3∆0 leu2∆0 his3∆0 mre11ΔKAN RFA1-MN::KAN* | This study |
| BYxrs2 | *MAT a met15∆0 lys2∆0 ura3∆0 leu2∆0 his3∆0 xrs2ΔKAN* | Euroscarf |
| BYRfa1MNxr2-1C  BYRfa1MNxr2-2A | *MAT alfa met15∆0 lys2∆0 ura3∆0 leu2∆0 his3∆0 xrs2ΔKAN RFA1-MN::KAN* | This study |
| BYrad55 | *MAT a met15∆0 lys2∆0 ura3∆0 leu2∆0 his3∆0 rad55ΔKAN* | Euroscarf |
| ByRfa1MNr55-3D  ByRfa1MNr55-2D | *MAT alfa met15∆0 lys2∆0 ura3∆0 leu2∆0 his3∆0 rad55ΔKAN RFA1-MN::KAN* | This study |
| BYmms22 | *MAT a met15∆0 lys2∆0 ura3∆0 leu2∆0 his3∆0 mms22ΔKAN* | Euroscarf |
| ByRfa1MNm22-1A  ByRfa1MNm22-3A | *MAT a met15∆0 lys2∆0 ura3∆0 leu2∆0 his3∆0 mms22ΔKAN RFA1-MN::KAN* | This study |
| BYpmr1 | *MAT a met15∆0 lys2∆0 ura3∆0 leu2∆0 his3∆0 pmr1ΔKAN* | Euroscarf |
| NGY004 | *MATa leu2-3,112 trp1-1 ura3-1 ade2-1 his3-11,15 can1-100 pmr1ΔATG::NAT* | Ralf Wellinger |
| BYrtt105 | *MAT a met15∆0 lys2∆0 ura3∆0 leu2∆0 his3∆0 rtt105ΔKAN* | Euroscarf |
| BYRfa1MNrt105-1C | *MAT a met15∆0 lys2∆0 ura3∆0 leu2∆0 his3∆0 rtt105ΔKAN RFA1-MN::KAN* | This study |
| BYtof1 | *MAT a met15∆0 lys2∆0 ura3∆0 leu2∆0 his3∆0 tof1ΔKAN* | Euroscarf |
| BYRfa1MNtf1-5B  BYRfa1MNtf1-5D | *MAT a MET15 lys2∆0 ura3∆0 leu2∆0 his3∆0 tof1ΔKAN RFA1-MN::KAN*  *MAT a met15∆0 LYS2 ura3∆0 leu2∆0 his3∆0 tof1ΔKAN RFA1-MN::KAN* | This study |
| BYlrp1 | *MAT a met15∆0 lys2∆0 ura3∆0 leu2∆0 his3∆0 lrp1ΔKAN* | Euroscarf |
| BYRfa1MNlp1-1C  BYRfa1MNlp1-1D | *MAT alfa MET15 lys2∆0 ura3∆0 leu2∆0 his3∆0 lrp1ΔKAN RFA1-MN::NAT*  *MAT a MET15 LYS2 ura3∆0 leu2∆0 his3∆0 lrp1ΔKAN RFA1-MN::NAT* | This study |
| BYrad50 | *MAT a met15∆0 lys2∆0 ura3∆0 leu2∆0 his3∆0 rad50ΔKAN* | Euroscarf |
| BYRfa1MNr50-3D  BYRfa1MNr50-4B | *MAT a met15∆0 lys2∆0 ura3∆0 leu2∆0 his3∆0 rad50ΔKAN RFA1-MN::KAN* | This study |
| BYchl1 | *MAT a met15∆0 lys2∆0 ura3∆0 leu2∆0 his3∆0 chl1ΔKAN* | Euroscarf |
| BYRfa1MNch1-1  BYRfa1MNch1-2 | *MAT a met15∆0 lys2∆0 ura3∆0 leu2∆0 his3∆0 chl1ΔKAN RFA1-MN::KAN* | This study |
| BYydj1 | *MAT a met15∆0 lys2∆0 ura3∆0 leu2∆0 his3∆0 ydj1ΔKAN* | Euroscarf |
| BYRfa1MNy1-2D  BYRfa1MNy1-3D | *MAT a MET15 LYS2 ura3∆0 leu2∆0 his3∆0 ydj1ΔKAN RFA1-MN::NAT* | This study |
| BYdia2 | *MAT a met15∆0 lys2∆0 ura3∆0 leu2∆0 his3∆0 dia2ΔKAN* | Euroscarf |
| BYRfa1MNd2-2A  BYRfa1MNd2-2B | *MAT a MET15 LYS2 ura3∆0 leu2∆0 his3∆0 dia2ΔKAN RFA1-MN::NAT* | This study |
| BYnup120 | *MAT a met15∆0 lys2∆0 ura3∆0 leu2∆0 his3∆0 nup120ΔKAN* | Euroscarf |
| BYRfa1MNn120-2C  BYRfa1MNn120-2D | *MAT a met15∆0 LYS2 ura3∆0 leu2∆0 his3∆0 nup120ΔKAN RFA1-MN::NAT*  *MAT a MET15 lys2∆0 ura3∆0 leu2∆0 his3∆0 nup120ΔKAN RFA1-MN::NAT* | This study |
| BYrad17 | *MAT a met15∆0 lys2∆0 ura3∆0 leu2∆0 his3∆0 rad17ΔKAN* | Euroscarf |
| BYRfa1MNr17-1C  BYRfa1MNr17-2A | *MAT alfa met15∆0 lys2∆0 ura3∆0 leu2∆0 his3∆0 rad17ΔKAN RFA1-MN::KAN*  *MAT alfa MET15 LYS2 ura3∆0 leu2∆0 his3∆0 rad17ΔKAN RFA1-MN::KAN* | This study |
| BYddc1 | *MAT a met15∆0 lys2∆0 ura3∆0 leu2∆0 his3∆0 ddc1ΔKAN* | Euroscarf |
| BYRfa1MNdd1-1B  BYRfa1MNdd1-2C | *MAT a MET15 lys2∆0 ura3∆0 leu2∆0 his3∆0 ddc1ΔKAN RFA1-MN::KAN*  *MAT a MET15 LYS2∆0 ura3∆0 leu2∆0 his3-0 ddc1ΔKAN RFA1-MN::KAN* | This study |
| BYhst3 | *MAT a met15∆0 lys2∆0 ura3∆0 leu2∆0 his3∆0 hst3ΔKAN* | Euroscarf |
| BYRfa1MNh3-4D  BYRfa1MNh3-9C | *MAT a MET15 lys2∆0 ura3∆0 leu2∆0 his3∆0 hst3ΔKAN RFA1-MN::KAN*  *MAT a met15∆0 LYS2 ura3∆0 leu2∆0 his3∆0 hst3ΔKAN RFA1-MN::KAN* | This study |
| BYmrc1 | *MAT a met15∆0 lys2∆0 ura3∆0 leu2∆0 his3∆0 mrc1ΔKAN* | Euroscarf |
| BYRfa1MNmc1-1C  BYRfa1MNmc1-6D | *MAT a MET15 LYS2 ura3∆0 leu2∆0 his3∆0 mrc1ΔKAN RFA1-MN::NAT* | This study |
| BYmec3 | *MAT a met15∆0 lys2∆0 ura3∆0 leu2∆0 his3∆0 mec3ΔKAN* | Euroscarf |
| BYRfa1MNmc3-8A  BYRfa1MNmc3-8B | *MAT a met15∆0 lys2∆0 ura3∆0 leu2∆0 his3∆0 mec3ΔKAN RFA1-MN::KAN*  *MAT a met15∆0 LYS2 ura3∆0 leu2∆0 his3∆0 mec3ΔKAN RFA1-MN::KAN* | This study |
| BYmms1 | *MAT a met15∆0 lys2∆0 ura3∆0 leu2∆0 his3∆0 mms1ΔKAN* | Euroscarf |
| BYRfa1MNmm1-8B  BYRfa1MNmm1-8D | *MAT a met15∆0 LYS2 ura3∆0 leu2∆0 his3∆0 mms1ΔKAN RFA1-MN::KAN*  *MAT a MET15 lys2∆0 ura3∆0 leu2∆0 his3∆0 mms1ΔKAN RFA1-MN::KAN* | This study |
| BYrtt107 | *MAT a met15∆0 lys2∆0 ura3∆0 leu2∆0 his3∆0 rtt107ΔKAN* | Euroscarf |
| BYRfa1MNr107-3A  BYRfa1MNr107-3D | *MAT a MET15 LYS2 ura3∆0 leu2∆0 his3∆0 rtt107ΔKAN RFA1-MN::KAN*  *MAT alfa MET15 LYS2 ura3∆0 leu2∆0 his3∆0 rtt107ΔKAN RFA1-MN::KAN* | This study |
| BYnpt1 | *MAT a met15∆0 lys2∆0 ura3∆0 leu2∆0 his3∆0 npt1ΔKAN* | Euroscarf |
| BYRfa1MNnp1-1A  BYRfa1MNnp1-1C | *MAT alfa MET15 lys2∆0 ura3∆0 leu2∆0 his3∆0 npt1ΔKAN RFA1-MN::KAN*  *MAT a MET15 lys2∆0 ura3∆0 leu2∆0 his3∆0 npt1ΔKAN RFA1-MN::KAN* | This study |
| BYpsy3 | *MAT a met15∆0 lys2∆0 ura3∆0 leu2∆0 his3∆0 psy3ΔKAN* | Euroscarf |
| BYRfa1MNpy3-5C  BYRfa1MNpy3-6A | *MAT a MET15 lys2∆0 ura3∆0 leu2∆0 his3∆0 psy3ΔKAN RFA1-MN::KAN*  *MAT a met15∆0 LYS2 ura3∆0 leu2∆0 his3∆0 psy3ΔKAN RFA1-MN::KAN* | This study |
| BYctf8 | *MAT a met15∆0 lys2∆0 ura3∆0 leu2∆0 his3∆0 ctf8ΔKAN* | Euroscarf |
| BYRfa1MNcf8-1A  BYRfa1MNcf8-1D | *MAT a met15∆0 LYS2 ura3∆0 leu2∆0 his3∆0 ctf8ΔKAN RFA1-MN::KAN*  *MAT a MET15 lys2∆0 ura3∆0 leu2∆0 his3∆0 ctf8ΔKAN RFA1-MN::KAN* | This study |
| BYrad61 | *MAT a met15∆0 lys2∆0 ura3∆0 leu2∆0 his3∆0 rad61ΔKAN* | Euroscarf |
| BYRfa1MNr61-1B  BYRfa1MNr61-1D | *MAT alfa MET15 LYS2 ura3∆0 leu2∆0 his3∆0 rad61ΔKAN RFA1-MN::KAN* | This study |
| BYpol32 | *MAT a met15∆0 lys2∆0 ura3∆0 leu2∆0 his3∆0 pol32ΔKAN* | Euroscarf |
| BYRfa1MNp32-7A  BYRfa1MNp32-77C | *MAT a met15∆0 LYS2 ura3∆0 leu2∆0 his3∆0 pol32ΔKAN RFA1-MN::KAN* | This study |
| BYdun1 | *MAT a met15∆0 lys2∆0 ura3∆0 leu2∆0 his3∆0 dun1ΔKAN* | Euroscarf |
| BYRfa1MNdu1-10A  BYRfa1MNdu1-10C | *MAT a MET15 LYS2 ura3∆0 leu2∆0 his3∆0 dun1ΔKAN RFA1-MN::NAT* | This study |
| WOE 11B | *MATalfa sml1Δ::kanMX trp1::pRS304-GAL-RNR2-TRP1 his3::pRS303-GAL-RNR3-HIS3 leu2::pRS305-GAL-RNR4-LEU2* | This study |
| WRfa1MN OE | *MATa sml1Δ::kanMX trp1::pRS304-GAL-RNR2-TRP1 his3::pRS303-GAL-RNR3-HIS3 leu2::pRS305-GAL-RNR4-LEU2 RFA1-MN::NAT* | This study |
| WOEdu1 | *MATa sml1Δ::kanMX trp1::pRS304-GAL-RNR2-TRP1 his3::pRS303-GAL-RNR3-HIS3 leu2::pRS305-GAL-RNR4-LEU2 dun1ΔHYG* | This study |
| WRfa1MN OEdu1 | *MATa sml1Δ::kanMX trp1::pRS304-GAL-RNR2-TRP1 his3::pRS303-GAL-RNR3-HIS3 leu2::pRS305-GAL-RNR4-LEU2 dun1ΔHYG RFA1-MN::NAT* | This study |
| BYcsm3 | *MAT a met15∆0 lys2∆0 ura3∆0 leu2∆0 his3∆0 csm3ΔKAN* | Euroscarf |
| BYRfa1MNcs3-3A  BYRfa1MNcs3-3C | *MAT a met15∆0 LYS2 ura3∆0 leu2∆0 his3∆0 csm3ΔKAN RFA1-MN::NAT*  *MAT a MET15 lys2∆0 ura3∆0 leu2∆0 his3∆0 csm3ΔKAN RFA1-MN::NAT* | This study |
| BYchd1 | *MAT a met15∆0 lys2∆0 ura3∆0 leu2∆0 his3∆0 chd1ΔKAN* | Euroscarf |
| BYRf1MNch1-4A  BYRf1MNch1-4C | *MAT alfa met15∆0 lys2∆0 ura3∆0 leu2∆0 his3∆0 chd1ΔKAN RFA1-MN::KAN*  *MAT a met15∆0 lys2∆0 ura3∆0 leu2∆0 his3∆0 chd1ΔKAN RFA1-MN::KAN* | This study |
| BYrad24 | *MAT a met15∆0 lys2∆0 ura3∆0 leu2∆0 his3∆0 rad24ΔKAN* | Euroscarf |
| BYRfa1MNr24-4C  BYRfa1MNr24-7A | *MAT a MET15 lys2∆0 ura3∆0 leu2∆0 his3∆0 rad24ΔKAN RFA1-MN::NAT*  *MAT a MET15 LYS2 ura3∆0 leu2∆0 his3∆0 rad24ΔKAN RFA1-MN::NAT* | This study |
| BYsch9 | *MAT a met15∆0 lys2∆0 ura3∆0 leu2∆0 his3∆0 sch9ΔKAN* | Euroscarf |
| BYRfa1MNs9-1A  BYRfa1MNs9-3D | *MAT a met15∆0 LYS2 ura3∆0 leu2∆0 his3∆0 sch9ΔKAN RFA1-MN::NAT* | This study |
| BYrtt109 | *MAT a met15∆0 lys2∆0 ura3∆0 leu2∆0 his3∆0 rtt109ΔKAN* | Euroscarf |
| BYRfa1MNr19-2A  BYRfa1MNr19-2B | *MAT a MET15 lys2∆0 ura3∆0 leu2∆0 his3∆0 rtt109ΔKAN RFA1-MN::KAN*  *MAT alfa MET15 lys2∆0 ura3∆0 leu2∆0 his3∆0 rtt109ΔKAN RFA1-MN::KAN* | This study |
| BYdcc1 | *MAT a met15∆0 lys2∆0 ura3∆0 leu2∆0 his3∆0 dcc1ΔKAN* | Euroscarf |
| BYRfa1MNdc1-1B  BYRfa1MNdc1-2C | *MAT a MET15 lys2∆0 ura3∆0 leu2∆0 his3∆0 dcc1ΔKAN RFA1-MN::KAN* | This study |
| BYsir3 | *MAT a met15∆0 lys2∆0 ura3∆0 leu2∆0 his3∆0 sir3ΔKAN* | Euroscarf |
| BYRfa1MNsr3-1  BYRfa1MNsr3-1 | *MAT a met15∆0 lys2∆0 ura3∆0 leu2∆0 his3∆0 sir3ΔKAN RFA1-MN::HIS* | This study |
| BYsir2 | *MAT a met15∆0 lys2∆0 ura3∆0 leu2∆0 his3∆0 sir2ΔKAN* | Euroscarf |
| BYRfa1MNsr2-1  BYRfa1MNsr2-1 | *MAT a met15∆0 lys2∆0 ura3∆0 leu2∆0 his3∆0 sir2ΔKAN RFA1-MN::HIS* | This study |
| BYrad59 | *MAT a met15∆0 lys2∆0 ura3∆0 leu2∆0 his3∆0 rad59ΔKAN* | Euroscarf |
| BYRfa1MNr59-3C  BYRfa1MNr59-4D | *MAT a MET15 LYS2 ura3∆0 leu2∆0 his3∆0 rad59ΔKAN RFA1-MN::NAT*  *MAT a met15∆0 lys2∆0 ura3∆0 leu2∆0 his3∆0 rad59ΔKAN RFA1-MN::NAT* | This study |
| BYbre1 | *MAT a met15∆0 lys2∆0 ura3∆0 leu2∆0 his3∆0 bre1ΔKAN* | Euroscarf |
| BYRfa1MNbe1-5A  BYRfa1MNbe1-5B | *MAT alfa MET15 lys2∆0 ura3∆0 leu2∆0 his3∆0 bre1ΔKAN RFA1-MN::KAN*  *MAT alfa MET15 LYS2 ura3∆0 leu2∆0 his3∆0 bre1ΔKAN RFA1-MN::KAN* | This study |
| BYarp5 | *MAT a met15∆0 lys2∆0 ura3∆0 leu2∆0 his3∆0 arp5ΔKAN* | Euroscarf |
| BYRfa1MNap5-1B  BYRfa1MNap5-2D | *MAT a met15∆0 LYS2 ura3∆0 leu2∆0 his3∆0 arp5ΔKAN RFA1-MN::NAT*  *MAT a MET15 lys2∆0 ura3∆0 leu2∆0 his3∆0 arp5ΔKAN RFA1-MN::NAT* | This study |
| BYrad9 | *MAT a met15∆0 lys2∆0 ura3∆0 leu2∆0 his3∆0 rad9ΔKAN* | Euroscarf |
| BYRfa1MNr9-7A  BYRfa1MNr9-7B | *MAT a MET15 LYS2 ura3∆0 leu2∆0 his3∆0 rad9ΔKAN RFA1-MN::NAT*  *MAT alfa MET15 lys2∆0 ura3∆0 leu2∆0 his3∆0 rad9ΔKAN RFA1-MN::NAT* | This study |
| BYsic1 | *MAT a met15∆0 lys2∆0 ura3∆0 leu2∆0 his3∆0 sic1ΔKAN* | Euroscarf |
| BYRfa1MNsc1-1D  BYRfa1MNsc1-2B | *MAT a met15∆0 lys2∆0 ura3∆0 leu2∆0 his3∆0 sic1ΔKAN RFA1-MN::KAN*  *MAT alfa MET15 LYS2 ura3∆0 leu2∆0 his3∆0 sic1ΔKAN RFA1-MN::KAN* | This study |
| BYrvs161 | *MAT a met15∆0 lys2∆0 ura3∆0 leu2∆0 his3∆0 rvs161ΔKAN* | Euroscarf |
| BYRfa1MNr161-1A  BYRfa1MNr161-1C | *MAT alfa MET15 lys2∆0 ura3∆0 leu2∆0 his3∆0 rvs161ΔKAN RFA1-MN::KAN*  *MAT a MET15 LYS2 ura3∆0 leu2∆0 his3∆0 rvs161ΔKAN RFA1-MN::KAN* | This study |
| BYctf4 | *MAT a met15∆0 lys2∆0 ura3∆0 leu2∆0 his3∆0 ctf4ΔKAN* | Euroscarf |
| BYRfa1MNcf4-1A  BYRfa1MNcf4-1D | *MAT alfa met15∆0 LYS2 ura3∆0 leu2∆0 his3∆0 ctf4ΔKAN RFA1-MN::KAN*  *MAT a MET15 lys2∆0 ura3∆0 leu2∆0 his3∆0 ctf4ΔKAN RFA1-MN::KAN* | This study |
| W303dia2 | *MATa leu2-3,112 trp1-1 ura3-1 ade2-1 his3-11,15 can1-100 dia2ΔHIS* | [3] |
| WRfa1MNd2-1D  WRfa1MNd2-2A | *MATalfa leu2-3,112 trp1-1 ura3-1 ade2-1 his3-11,15 can1-100 dia2ΔHIS RFA1-MN::KAN* | This study |
| W303dia2::TPR | *MATa leu2-3,112 trp1-1 ura3-1 ade2-1 his3-11,15 can1-100 dia2ΔTPR::HYG* | [3] |
| WRfa1MNd2TPR-1C  WRfa1MNd2TPR-3A | *MATa leu2-3,112 trp1-1 ura3-1 ade2-1 his3-11,15 can1-100 dia2ΔTPR::HYG RFA1-MN::KAN* | This study |
| BYexo1 | *MAT a met15∆0 lys2∆0 ura3∆0 his3∆0 exo1ΔLEU2* | This study |
| BYRfa1MNe1-2B  BYRfa1MNe1-2D | *MAT a met15∆0 LYS2 ura3∆0 his3∆0 exo1ΔLEU2 RFA1-MN::KAN* | This study |
| BYe1s1 | *MAT alfa met15∆0 lys2∆0 ura3∆0 his3∆0 exo1ΔLEU2 sgs1ΔKAN bar1ΔHYG* | This study |
| BYRfa1MNs1-2A  BYRfa1MNs1-2D | *MAT a met15∆0 LYS2 ura3∆0 his3∆0 exo1ΔLEU2 RFA1-MN::NAT* | This study |
| BYsae2 | *MAT a met15∆0 lys2∆0 ura3∆0 leu2∆0 his3∆0 sae2ΔKAN* | Euroscarf |
| BYRfa1MNs2-9B  BYRfa1MNs2-9C | *MAT alfa met15∆0 LYS2 ura3∆0 leu2∆0 his3∆0 sae2ΔKAN RFA1-MN::KAN*  *MAT a MET15 LYS2 ura3∆0 leu2∆0 his3∆0 sae2ΔKAN RFA1-MN::KAN* | This study |
| Wsmc3-42-1D | *MAT alfa leu2-3,112 trp1-1 ura3-1 ade2-1 his3-11,15 can1-100 smc3-42* | This study |
| WRfa1MN sc3-42-1A  WRfa1MN sc3-42-2D | *MATa leu2-3,112 trp1-1 ura3-1 ade2-1 his3-11,1515 can1-100 BAR1 RAD5 smc3-42 RFA1-MN::KAN* | This study |
| BYdat1 | *MAT a met15∆0 lys2∆0 ura3∆0 leu2∆0 his3∆0 dat1ΔKAN* | Euroscarf |
| BYRfa1MNdt1-10B  BYRfa1MNdt1-10D | *MAT alfa MET15 lys2∆0 ura3∆0 leu2∆0 his3∆0 dat1ΔKAN RFA1-MN::KAN* | This study |
| BYcdh1 | *MAT a met15∆0 lys2∆0 ura3∆0 leu2∆0 his3∆0 cdh1ΔKAN* | Euroscarf |
| BYRfa1MNch1-4A  BYRfa1MNch1-5B | *MAT a MET15 LYS2 ura3∆0 leu2∆0 his3∆0 cdh1ΔKAN RFA1-MN::KAN*  *MAT a met15∆0 lys2∆0 ura3∆0 leu2∆0 his3∆0 cdh1ΔKAN RFA1-MN::KAN* | This study |
| BYmdm20 | *MAT a met15∆0 lys2∆0 ura3∆0 leu2∆0 his3∆0 mdm20ΔKAN* | Euroscarf |
| BYRfa1MNm20-2A | *MAT alfa MET15 lys2∆0 ura3∆0 leu2∆0 his3∆0 mdm20ΔKAN RFA1-MN::NAT* | This study |
| BYchk1sml1rad53 | *MAT a met15∆0 lys2∆0 ura3∆0 leu2∆0 his3∆0 chk1ΔKAN sml1ΔHYG rad53ΔNAT* | This study |
| BYRfa1MNck1sm1 r53-1A  BYRfa1MNck1sm1 r53-4A | *MAT a met15∆0 lys2∆0 ura3∆0 leu2∆0 his3∆0 chk1ΔKAN sml1ΔHYG rad53ΔNAT RFA1-MN::KAN* | This study |
| BYrad27 | *MAT a met15∆0 lys2∆0 ura3∆0 leu2∆0 his3∆0 rad27ΔKAN* | Euroscarf |
| BYRfa1MNr27-8A  BYRfa1MNr27-10C | *MAT a met15∆0 LYS2 ura3∆0 leu2∆0 his3∆0 rad27ΔKAN RFA1-MN::NAT*  *MAT a MET15 lys2∆0 ura3∆0 leu2∆0 his3∆0 rad27ΔKAN RFA1-MN::NAT* | This study |
| BYcsm2 | *MAT a met15∆0 lys2∆0 ura3∆0 leu2∆0 his3∆0 csm2ΔKAN* | Euroscarf |
| BYRfa1MNcs2-1B  BYRfa1MNcs2-2B | *MAT a MET15 lys2∆0 ura3∆0 leu2∆0 his3∆0 csm2ΔKAN RFA1-MN::NAT* | This study |
| BYxrn1 | *MAT a met15∆0 lys2∆0 ura3∆0 leu2∆0 his3-0 xrn1ΔKAN* | Euroscarf |
| BYRfa1MNx1-1C  BYRfa1MNx1-2D | *MAT a met15∆0 LYS2 ura3∆0 leu2∆0 his3∆0 xrn1ΔKAN RFA1-MN::NAT*  *MAT a met15∆0 lys2∆0 ura3∆0 leu2∆0 his3∆0 xrn1ΔKAN RFA1-MN::NAT* | This study |
| BYrad18 | *MAT a met15∆0 lys2∆0 ura3∆0 leu2∆0 his3-0 rad18ΔKAN* | Euroscarf |
| BYRfa1MNr18-7A  BYRfa1MNr18-11A | *MAT a MET15 lys2∆0 ura3∆0 leu2∆0 his3∆0 rad18ΔKAN RFA1-MN::NAT* | This study |
| BYrtt101 | *MAT a met15∆0 lys2∆0 ura3∆0 leu2∆0 his3-0 rtt101ΔHYG* | This study |
| BYRfa1MNr101-1  BYRfa1MNr101-2 | *MAT alfa MET15 lys2∆0 ura3∆0 leu2∆0 his3∆0 rtt101ΔHYG RFA1-MN::NAT* | This study |
| BYcac1rtt106-3B | *MAT a met15∆0 lys2∆0 ura3∆0 leu2∆0 his3-0 bar1ΔHYG cac1ΔKAN rtt106ΔKAN* | This study |
| BYRfa1MNc1r106-8A  BYRfa1MNc1r106-8D | *MAT a met15∆0 lys2∆0 ura3∆0 leu2∆0 his3∆0 cac1ΔKAN rtt106ΔKAN RFA1-MN::NAT* | This study |
| BYctf18 | *MAT a met15∆0 lys2∆0 ura3∆0 leu2∆0 his3-0 ctf18ΔHYG* | This study |
| BYRfa1MNcf18-1  BYRfa1MNcf18-2 | *MAT alfa MET15 lys2∆0 ura3∆0 leu2∆0 his3∆0 ctf18ΔHYG RFA1-MN::NAT* | This study |
| W303sc6-56-4D | *MATa leu2-3,112 trp1-1 ura3-1 ade2-1 his3-11,1515 can1-100 BAR1 RAD5 smc6-56::KAN* | This study |
| WRfa1MNsc6-56-5C  WRfa1MNsc6-56-16B | *MATa leu2-3,112 trp1-1 ura3-1 ade2-1 his3-11,1515 can1-100 BAR1 RAD5 smc6-56::KAN RFA1-MN::KAN* | This study |
| W303scc1-73 | *MATa leu2-3,112 trp1-1 ura3-1 ade2-1 his3-11,1515 can1-100 BAR1 RAD5 scc1-73::TRP1* | This study |
| WRfa1MNscc1-73-3C  WRfa1MNscc1-73-5C | *MATa leu2-3,112 trp1-1 ura3-1 ade2-1 his3-11,1515 can1-100 BAR1 RAD5 scc1-73::TRP1 RFA1-MN::KAN* | This study |
| BYgcn5 | *MAT alfa met15∆0 lys2∆0 ura3∆0 leu2∆0 his3∆0 gcn5ΔHYG* | This study |
| BYRfa1MNgcn5-1  BYRfa1MNgcn5-2 | *MAT alfa MET15 lys2∆0 ura3∆0 leu2∆0 his3∆0 gcn5ΔHYG RFA1-MN::NAT* | This study |
| BYku70 | *MAT a met15∆0 lys2∆0 ura3∆0 leu2∆0 his3∆0 ku70ΔKAN* | Euroscarf |
| BYMNku70-2D  BYMNku70-5C | *MAT a MET15 lys2∆0 ura3∆0 leu2∆0 his3∆0 ku70ΔKAN RFA1-MN::NAT* | This study |
| BYku70r52 | *MAT a met15∆0 lys2∆0 ura3∆0 leu2∆0 his3∆0 ku70ΔKAN rad52 ΔKAN* | This study |
| DMY155-39C | *MATa leu2-3,112 trp1-1 ura3-1 ade2-1 his3-11,15 can1-100 bar1ΔNAT spt16-m::NAT* | [4] |
| WRfa1MNsm-7B | *MATa leu2-3,112 trp1-1 ura3-1 ade2-1 his3-11,15 can1-100 bar1ΔNAT spt16-m::NAT RFA1-MN::KAN* | This study |
| W303sm-32A | *MATa leu2-3,112 trp1-1 ura3-1 ade2-1 his3-11,15 can1-100 bar1ΔNAT HMLΔLEU spt16-m::NAT cac1ΔKAN rtt106ΔHYG* | [4] |
| W303Rfa1MNsm-1B  W303Rfa1MNsm-2A | *MATa leu2-3,112 trp1-1 ura3-1 ade2-1 his3-11,15 can1-100*  *HMLΔLEU spt16-m::NAT cac1ΔKAN rtt106ΔHYG RFA1-MN::KAN* | This study |
| BYpif1-3 | *MAT a met15∆0 LYS2 ura3∆0 leu2∆0 his3∆0 pif1ΔKAN* | This study |
| BYRfa1MNpf1-5C  BYRfa1MNpf1-5D | *MAT a MET15 LYS2 ura3∆0 leu2∆0 his3∆0 pif1ΔKAN RFA1-MN::KAN* | This study |
| BYmus81 | *MAT a MET15 LYS2 ura3∆0 leu2∆0 his3∆0 mus81ΔKAN* | This study |
| BYRfa1MNm81-1B  BYRfa1MNm81-51B | *MAT a MET15 LYS2 ura3∆0 leu2∆0 his3∆0 mus81ΔKAN RFA1-MN::KAN bar1ΔHYG* | This study |
| BYyen1 | *MAT a met15∆0 lys2∆0 ura3∆0 leu2∆0 his3∆0 yen1ΔKAN* | Euroscarf |
| BYRfa1MNyen1 | *MAT a met15∆0 lys2∆0 ura3∆0 leu2∆0 his3∆0 yen1ΔKAN RFA1-MN::NAT* | This study |
| BYm81yen1 | *MAT a met15∆0 lys2∆0 ura3∆0 leu2∆0 his3∆0 mus81ΔKAN yen1ΔKAN* | This study |
| BYRfa1MNm81yen1 | *MAT a met15∆0 lys2∆0 ura3∆0 leu2∆0 his3∆0 mus81ΔKAN yen1ΔKAN RFA1-MN::NAT* | This study |
| W303mcm2-3A | *MAT a leu2-3,112 trp1-1 ura3-1 ade2-1 his3-11,15 can1-100 mcm2-3A::HYG* | This study |
| WRfa1MNmc2-2A  WRfa1MNmc2-8A | *MAT a leu2-3,112 trp1-1 ura3-1 his3-11,15 can1-100 mcm2-3A::HYG RFA1-MN::KAN*  *MAT alfa leu2-3,112 trp1-1 ura3-1 his3-11,15 can1-100 mcm2-3A::HYG RFA1-MN::KAN* | This study |
| Wpol1-2A2 | *MATa leu2-3,112 trp1-1 ade2-1 his3-11,15 can1-100 pol1-2a2::HYG* | [5] |
| WRfa1MNp1-6A | *MATalfa leu2-3,112 trp1-1 ura3-1 ade2-1 his3-11,15 can1-100 pol1-2A2::HYG RFA1-MN::KAN* | This study |
| BYwhi5 | *MAT a met15∆0 lys2∆0 ura3∆0 leu2∆0 his3∆0 whi5ΔKAN* | Euroscarf |
| BYRfa1MNw5-20D  BYRfa1MNw5-20C | *MAT a met15∆0 ura3∆0 leu2∆0 his3∆0 whi5ΔKAN RFA1-MN::NAT*  *MAT alfa lys2∆0 ura3∆0 leu2∆0 his3∆0 whi5ΔKAN RFA1-MN::NAT* | This study |
| BYs1w5-5C  BYs1w5-3A | *MAT a met15∆0 lys2∆0 ura3∆0 leu2∆0 his3∆0 sic1ΔKAN whi5ΔKAN*  *MAT alfa lys2∆0 ura3∆0 leu2∆0 his3∆0 sic1ΔKAN whi5ΔKAN* | This study |
| BYRfa1MNs1w5-2A  BYRfa1MNs1w5-5D | *MAT a met15∆0 lys2∆0 ura3∆0 leu2∆0 his3∆0 sic1ΔKAN whi5ΔKAN RFA1-MN::NAT* | This study |
| WGpSIC1-3A | *MAT a leu2-3,112 TRP1 ade2-1 his3-11,15 can1-100 sic1::(TRP1)GAL1,10p-SIC1* | This study |
| WRfa1MNGpS1-7A | *MATalfa leu2-3,112 TRP1 ade2-1 his3-11,15 can1-100 sic1::(TRP1)GAL1,10p-SIC1 RFA1-MN::KAN* | This study |
| WGpSIC1c1-4B  WGpSIC1c1-5C | *MATalfa leu2-3,112 TRP1 ura3-1 ade2-1 HIS3 can1-100 sic1::(TRP1)GAL1,10p-SIC1 cdh1ΔHIS3* | This study |
| WRfa1MNGpS1c1-3B  WRfa1MNGpS1c1-6C | *MATalfa leu2-3,112 TRP1 ura3-1 ade2-1 HIS3 can1-100 sic1::(TRP1)GAL1,10p-SIC1 cdh1ΔHIS3 RFA1-MN::KAN* | This study |
| Wtop3 | *MAT a leu2-3,112 trp1-1 ura3-1 ade2-1 his3-11,15 can1-100 top3ΔNAT* | This study |
| WRfa1MNt3 | *MAT a leu2-3,112 trp1-1 ura3-1 ade2-1 his3-11,15 can1-100 top3ΔNAT RFA1-MN::KAN* | This study |
| Wr53K227A | *MAT a leu2-3,112 trp1-1 ura3-1 ade2-1 his3-11,15 can1-100 RAD5 rad53-K227A::KAN* | This study |
| WRfa1MNr53K227A | *MAT a leu2-3,112 trp1-1 ura3-1 ade2-1 his3-11,15 can1-100 RAD5 rad53-K227A::KAN RFA1-MN::KAN* | This study |
| Wdpb3 | *MAT a leu2-3,112 trp1-1 ura3-1 ade2-1 his3-11,15 can1-100 dpb3ΔKAN* | This study |
| WRfa1MNdp3 | *MAT a leu2-3,112 trp1-1 ura3-1 ade2-1 his3-11,15 can1-100 dpb3ΔKAN RFA1-MN::KAN* | This study |

All strains are isogenic to BY4741 (BY strains) or W303-1A (W strains and NGY004)

1. Kuzmin E, Costanzo M, Andrews B, Boone C. Synthetic Genetic Array Analysis. Cold Spring Harb Protoc. 2016;2016: pdb.prot088807. doi:10.1101/pdb.prot088807

2. González-Prieto R, Muñoz-Cabello AM, Cabello-Lobato MJ, Prado F. Rad51 replication fork recruitment is required for DNA damage tolerance. EMBO J. 2013;32: 1307–1321. doi:10.1038/emboj.2013.73

3. Morohashi H, Maculins T, Labib K. The Amino-Terminal TPR Domain of Dia2 Tethers SCFDia2 to the Replisome Progression Complex. Curr Biol. 2009;19: 1943–1949. doi:10.1016/j.cub.2009.09.062

4. Barrientos-Moreno M, Maya-Miles D, Murillo-Pineda M, Fontalva S, Pérez-Alegre M, Andujar E, et al. Transcription and FACT facilitate the restoration of replication-coupled chromatin assembly defects. Sci Rep. 2023;13: 11397. doi:10.1038/s41598-023-38280-w

5. González-Garrido C, Prado F. Parental histone distribution and location of the replication obstacle at nascent strands control homologous recombination. Cell Rep. 2023;42: 112174. doi:10.1016/j.celrep.2023.112174
