# Supplementary material for: A Rfa1-MN–based system reveals new factors involved in the rescue of broken replication forks
Source: PLoS Genet. 2025 Apr 1;21(4):e1011405. doi: 10.1371/journal.pgen.1011405 (PMC11984746; doi:10.1371/journal.pgen.1011405)
Supplement: Attachment — Submitted filename: Amiama-Roy PLoS Genetics.pdf [file pgen.1011405.s010.pdf]

Amiama-Roig et al PLoS Genetics

In this study, the authors exploited a Rfa1-micrococcal nuclease (MN) chimera to induce double-strand breaks (DSBs) in the budding yeast genome. Given that RPA (Rfa1 encodes the largest subunit of the RPA complex) coated single-stranded DNA (ssDNA) is generated during DNA replication, the authors predicted that Rfa1-MN would generate DSBs specifically at replication forks. Cells expressing the Rfa1-MN chimera grow normally but are dependent on the Rad52 protein for viability suggesting that native levels of Ca^{2+} can activate MN nuclease activity and create lesions during DNA replication that are repaired by homologous recombination (HR). To increase the damage induced by this system, the authors added Ca^{2+} to the growth media and demonstrated time dependent degradation of bulk genomic DNA. The authors screened a collection of strains involved in the DNA damage response to identify mutants defective for repair of DSBs at replication forks. The group most sensitive to Rfa1-MN expression were core HR genes including the *RAD52* epistasis group. Core HR mutants were found to be synthetic lethal when combined with the Rfa1-MN chimera allele, even in the absence of additional Ca^{2+} to the growth media. The authors also identified several pathways that are important for repair of DSBs at replication forks including the DNA replication checkpoint, the break-induced replication (BIR) pathway, replication coupled nucleosome assembly (RCNA), sister-chromatid cohesion and factors involved in G1 cell cycle regulation that showed growth defects. Mutants defective for these pathways showed Rfa1-MN sensitivity only when Ca^{2+} was added to the growth media

While this paper identifies numerous factors involved in repair of Rfa1-MN induced DSBs, questions remain about the specificity of this system to generate DSBs specifically at replication forks. The authors also make several vague or overinterpreted statements that are not well supported by the data presented in the manuscript.

Major comments:

1. While Rfa1-MN is most likely making breaks at the lagging strand of replication forks, the authors should consider the possibility that Rfa1-MN is making breaks in other contexts, such as single-strand gaps behind replication forks, at persistent R-loops, or at ssDNA revealed by the resection machinery. The authors might consider arresting cells in G1 or G2 to ensure that Ca^{2+}-dependent genomic DNA degradation requires passage through S- phase. At a minimum, the possibility of these non-replication fork breaks should be discussed.
2. Can the authors provide an estimate for how many breaks Rfa1-MN is generating with/without Ca^{2+} added to the growth media?
3. Interestingly, Rad53 is not phosphorylated in cells expressing Rfa1-MN, even after addition of 400 mM Ca^{2} to the growth medium. Is Rad53 activation observed in the repair-deficient *RFA1-MN rad51*Δ (or *rtt109*Δ) strain? It seem possible that the ssDNA required for Mec1 activation is being destroyed by Rfa1-MN.
4. If Rad53 is responsible for phosphorylating Dun1, it is confusing that the Rad53 mutant would not be sensitive to Rfa1-MN. It is possible that *sml1*Δ suppresses the *rad53*Δ phenotype by raising nucleotide pools. The authors should consider the use of a Rad53 kinase dead allele (such as *rad53K227A*) which does not require the additional deletion of *SML1* for cell viability. Similarly, since the authors argue that Dun1 activity

raises nucleotide pools for the repair process, they should test *sml1*Δ for suppression of the *RFA1-MN dun1*Δ phenotype.

5. The authors show that the fork protection complex (Tof1, Csm3) is required for viability of cells expressing Rfa1-MN. If forks are uncoupled in the absence of FPC, then more ssDNA may be generated at forks providing more substrates for MN cleavage. Thus, the synthetic defect could simply be due to increased damage as opposed to a direct role for these factors in repair. The same argument could be made for the *rad27*Δ mutation which is known to be synthetic lethal with HR defects, presumably due to a higher burden of S-phase associated DNA damage.
6. Figure 4 presents the most novel aspect of this paper by discussing the possibility that a short G1 phase limits origin licensing, which leads to hypersensitivity of replication fork breakage during S phase. However, the authors do not provide concrete mechanistic evidence that the mutants described have an origin licensing defect, or that this defect would in fact complicate repair of breaks at replication forks.

Minor Points:

1. Many of the spot assays have a high degree of background colonies. Additionally, it would be more visually appealing to display these assays in grayscale/black and white as the many different colors currently displayed is a bit distracting.
2. The authors note that Rfa1-MN *rad52*Δ is synthetic lethal and do not perform spot assays with this strain. The same is essentially true for Rfa1-MN combined with *pmr1*Δ*, rad51*Δ*, mre11*Δ*, rad50*Δ*, xrs2*Δ*, rad54*Δ*, rad55*Δ*, and rtt105*Δ. Double mutants isolated from pinprick colonies post dissection likely have suppressor mutations. As such, it does not seem appropriate to include spot assays for these mutants in the manuscript and to compare them with mutants that only show Rfa1-MN sensitivity in the presence of Ca^{2+} (e.g., *pol32*Δ)
3. On the last sentence of the first paragraph on page 12, I believe the authors meant to write “Ctf8 and Dcc1” instead of “Ctf18 and Ddc1”.
4. Figure S2B: At what temperature was the sensitivity of *smc6-56* mutant to Rfa1-MN tested?
5. With the exception of unique sequence specific or genetic contexts, Rad51 is required for BIR in yeast. BIR at generic replication fork breaks as described in this manuscript is Rad51 dependent, as indicated by the synthetic lethality for *RFA1-MN rad51*, and in other studies showing that Rad51 is required for nick-induced repair. As reported by Mayle et al (2015) for Flp-nick and in this study, Pol32 is not as important as Rad51.
6. The authors fail to cite work from the Luke lab showing that mis-regulation of RNaseH2 creates DNA damage during S-phase that requires HR and the RCNA pathway for repair (Schindler et al., 2023). This work should be cited in the Discussion.
