# Supplementary material for: A Rfa1-MN–based system reveals new factors involved in the rescue of broken replication forks
Source: PLoS Genet. 2025 Apr 1;21(4):e1011405. doi: 10.1371/journal.pgen.1011405 (PMC11984746; doi:10.1371/journal.pgen.1011405)
Supplement: Attachment — Submitted filename: Review_Plos Genetics-PGENETICS-D-24-00968.docx [file pgen.1011405.s011.docx]

In this manuscript, Amiama-Roig et al. have modified a previously used system in yeast, Chromatin Endogenous Cleavage (ChEC), to induce DNA double-strand breaks (DSB) at replication forks. The authors characterize the genetic requirements of a broken replication fork versus a broken linear DNA molecule. The authors performed a screen to identify several players involved in the repair of DSBs at replication forks. In particular, the authors implicate the requirement of various HR factors, BIR factors, and cell cycle factors in the repair of DSBs at replication forks.

Overall, although the authors have a novel tool to test the genetic requirements of broken forks, the system needs to be further validated, and the candidates identified require further investigation. Data obtained from the screen is over interpreted and provides limited mechanistic insight into the repair of broken replication forks.

Major comments:

1. Although the ChEC system has been previously used successfully, the Rfa1-MN chimera used in this manuscript needs to be further validated. The authors need to test the Rfa1-MN system in the presence of DSB-inducing drugs like bleomycin, zeocin and following exposure to ionizing radiation.

2. The authors need to provide evidence that the Rfa1-MN chimera is functional and makes physiological amounts of protein.

3. The authors need to discuss the precise nature of the substrate, and the frequency of DNA lesions formed in the lagging strand using the Rfa1-MN system in response to Ca2+ treatment, which remains unclear.

Minor comments:

1. Figure 1A: The concentration of Ca2+ has not been mentioned in the figure or the legend.

2. Figure 1E: The authors note that Rfa1-MN and rad52∆ are synthetic lethal, but Rfa1-MN and rad51∆ are viable in Figure 1H. The authors need to elaborate on the nature of the DNA lesion requiring Rad52.

3. Figure 1H: Why have the authors not included 400mM Ca2+ data for Rfa1-MN rad51∆. This is an important concentration, since the authors performed SGA analysis at this concentration.

4. Figure 1H: The labeling on the most bottom lane is unclear. Is it RFA1-MN, pol32∆ or double?

5. Figure 2B: The authors mention Rfa1-MN rad59∆ cells are synthetic sick. This would imply an annealing step in the repair of broken forks. The authors need to discuss this possibility in their model or discussion.

6. Figure 2B: The authors need to confirm the effects of Rfa1-MN rtt105∆ on conventional DSB repair in parallel to broken fork repair

7. Figure 3C: The Dun1 data is overinterpreted. There is no direct evidence provided suggesting an increase in dNTP pool facilitates Rfa1-MN mediated broken fork repair. This interpretation should be moved to the discussion. The authors could also test the strains in the presence of HU and Ca2+ if possible.

8. Figure 2H: It is relevant to test the role of yen1∆ and yen1∆ mus81∆ in their assays, since they mention it in the model

9. Figure 4B legend: needs to be elaborated

10. Discussion:

The authors imply seDSBs formed in the lagging strand at broken replication forks is mutagenic.

This is in stark contrast with recent data that the authors have cited (Pavani et al., Kimble et al., and Elango et al.), that do not require the arrival of a second fork. Please discuss.

The statement ‘*this result strongly suggests that converging forks also participate in the rescue of DSBs at forks.*’ needs to be re-worded since there is no direct quantification of the number of licensed origins.

The statement ‘*This situation was reverted in HR mutants, which is consistent with the restrictive effect of strand invasion on DNA resection at seDSBs’* needs to be re-worded. It is unclear what HR mutants and phenotypes the author is referring to.
