# Supplementary material for: A Rfa1-MN–based system reveals new factors involved in the rescue of broken replication forks
Source: PLoS Genet. 2025 Apr 1;21(4):e1011405. doi: 10.1371/journal.pgen.1011405 (PMC11984746; doi:10.1371/journal.pgen.1011405)
Supplement: Attachment — Submitted filename: Amiama.Barrientos.Rebuttal letter.docx [file pgen.1011405.s012.docx]

We appreciate the constructive comments and suggestions, which are discussed below. In addition to answer each specific point, the text has been extensively modified to accommodate all reviewer’ concerns and to get a more comprehensible reading. Note that we have slightly modified the title as the previous one suggested that all required factors had been identified in this screening. Now it reads: **A Rfa1-MN–based system reveals new factors involved in the rescue of broken replication forks.** In addition, we have also included the analysis of *top3∆*, a mutant that was identified in the initial screening but could not be validated by PCR in the collection mutant. This study provides a global overview of the process of broken fork repair through the identification of a number of mutans. In some cases, we could demonstrate the genetic pathway in which they are involved, but in general we suggest different possibilities inferred from their known phenotypes. We are aware that future molecular experiments will be required to test these hypotheses, as indicated in the final sentence of Discussion.

**Reviewer #1:** A Rfa1-MN–based system reveals the factors that participate in the rescue of broken replication forks

Broken replication forks are a major source of genomic instability, and the repair of broken forks is incompletely understood. Using budding yeast as a model system, this manuscript reports the use of a fusion protein between the large subunit of the single-stranded DNA binding protein RPA and micrococcal nuclease (MN) termed RFA1-MN. This genetic tool induces double-stranded DNA breaks near RPA bound to DNA upon the addition of calcium, which activates MN. Remarkably the fusion protein does not elicit MMS or HU sensitivity in otherwise wild type cells. The study reports a systematic screen with custom array of 358 gene deletion and identify some expected hits (HR, BIR proteins), as well as some unexpected hits (replisome factors, replisome stability factors, cohesion factors). Also of interest are the negative results as well as well the limited genetic interactions with chromatin components. Another interesting result concerns DDR signaling, in that Rad53 is not activated but Dun1 shows genetic interaction with RFA1-MN. This is unexpected from the canonical Mec1-Rad53-Dun1 pathway concept. Finally, the screen produced evidence that shortening the G1 cell cycle phase has an impact on RFA1-MN sensitivity.

Overall, the data are well presented and the conclusions generally well supported by the results. The hits in the screen have been validated, and some unexpected results were followed up with additional genetic experiments to support the conclusions. This screen opens many doors for follow-up and mechanistic analysis, producing a comprehensive view on processes and factors affecting repair of broken replication forks.

Major comments:

1) The premise appears to be that Rpa1-MN only cleaves replication forks. What is the evidence? There are other potential sources of ssDNA bound by RPA from other nuclear processes such as BER, NER, MMR, and transcription (R-loops). There should be some discussion of this point.

This has been supported with additional information and experiments. However, we have tried not to be so categoric as we cannot rule out additional cuts at other regions.

Supporting cleavage at non-replication forks, we show by ChEC that Rfa1-MN can cut DNA in G1 cells (new Figure 1A; previous ChEC is now as Figure S1A and it has been corrected because by mistake the kinetics was reported in seconds instead of minutes). This result is consistent with RPA localization at the transcribed regions of active genes (Sikorski et al. Mol Cell 2011). This is included in the characterization of the system in Results.

Supporting preferential cleavage at replications forks: We show that the kinetics of ChEC digestion is faster in S phase cells (new Figure 1A) and, importantly, Rfa1-MN is extremely efficient in digesting replication forks, as determined by 2D/ChEC, where replication intermediates from ChEC-treated cells are analysed by 2-dimensional gel electrophoresis (new Figure 1B; replication intermediates are completely digested in just 45 seconds) (we have performed this approach as ChEC preferentially detects DNA breaks at lineal molecules as the amount of forks relative to the whole genome is low). In addition, we confirmed previous studies by Haber’ group (Kramer et al. MCB 1994) showing that, in contrast to Rfa1-MN-induced DSBs where HR is essential, NHEJ can operate as a backup mechanism at canonical DSBs (new Figure 1J). These results are consistent with previous studies by ChIP-seq of whole chromosomes and microscopy analyses of RPA foci in G1 and S phases (Li et al. 2018 EMBO J, Li et al. 2022 EMBO J, Wong et al 2019 Mol Cell) that show a preferential accumulation of RPA at replication forks as compared with non-replicated regions in S phase and G1 (ChIP) and of RPA foci in S phase relative to G1 (microscopy). In summary, we conclude from our and previous results: “Although we cannot discard the formation of some DSBs at other regions, the preferential accumulation of RPA at replication forks [37–39], the high efficiency of Rfa1-MN to digest replication forks, and the essential role of HR for *RFA1-MN* cell viability suggest that most of these DSBs stem from Rfa1-MN–cut replication forks, preferentially at the lagging strand that accumulates most ssDNA/RPA (Figure 1K).” Likewise, we start Discussion by claiming: “In this study, we have generated a chimera of the largest subunit of the RPA complex with the MN that preferentially generates DSBs at replication forks and searched for mutants affected in their repair.” Actually, as now explained in Discussion to introduce the putative substrate for Rfa1-MN (graphically illustrated in new Figure 5), deDSBs can also be generated behind the fork between Okazaki fragments.

It is worth to note that the accumulation of seDSBs (that requires fork breakage) is also supported by the severe growth defect of the *RFA1-MN mus81∆ yen1∆*, because Mus81 and Yen1 are not required for SDSA (the main deDSB repair mechanism in mitotic cells). This argument has also been included in the Discussion.

2) In Figure 1A, there is some evidence of cleavage independent of exogenously added calcium, which is augmented in the presence of MMS. This should be discussed.

Rfa1-MN-induced cleavage in vivo, inferred from the lethality with HR core mutants, is not detected by ChEC. A slight smear is sometimes obtained below the major band due to fragmentation during DNA extraction. The observed profile is the one obtained upon DNA extraction regardless of the presence of Rfa1-MN or calcium (with and without MMS) (see new Figure S1A, right). In results, it reads: “Since the intracellular levels of Ca^2+^ ions are low for MN activation, this assay requires cells to be permeabilized with digitonin followed by addition of CaCl_2_ (Figure S1A).” An in the legend of Figure S1A: “Addition of Ca^2+^ is required for detection of Rfa1-MN-digested DNA, as determined by running total DNA of wild-type and *RFA1-MN* cells growing in the absence or presence of 0.005% MMS for 2 h (right).”

3) It is stated on page 7 (near top) that that RPA is primarily at the fork. What is the evidence for this, and please give references.

Please see our answer to point 1.

4) I was surprised that the MRN complex along with Sae2 is needed here, as it is not required in frank DSB repair unless the DSB ends are modified or covalently bound by a protein. Does this suggest that the ends or not free or otherwise modified that requires processing for Rad51 filament assembly? This aspect should be discussed.

Done as suggested; now it reads in Discussion: “Our results also show that the DNA resection factors Sae2 and Sgs1/Exo1 are not essential, which might be explained by the minimal resection that replication-born seDSBs require for strand invasion [105] and the likely preferential cleavage of the lagging strand by Rfa1-MN that would generate a 3’-ended ssDNA. In this regard, the essentiality of the MRX complex, also observed for Mre11 but not for its nuclease activity in nick-induced fork breakage [106], is likely more related to its replication fork stability and sister chromatid tethering activities [107,108]. Sae2 and Sgs1/Exo1 might be preferentially required for those cases in which Rfa1-MN cuts at the leading strand, which would lead to the formation of blunt or 5’-ssDNA ends. Sae2 would facilitate the removal of the Ku complex from these intermediates, in accordance with the increase in nick-induced BIR events in yeast cells lacking Ku70 [23].”

5) The effect of Rtt105 is curious, as it would been expected to suppress a negative effect of RFA1-MN, as it is a chaperone for RPA to bind ssDNA. This suggests that Rtt105 has other client proteins that are relevant here. This should be discussed more in detail.

Done as suggested; it was observed that the lack of Rtt105 has a strong impact in the amount of RPA at forks only under replicative stress (not under normal conditions), what would explain why Rfa1-MN causes DNA damage in the mutant. Although we cannot formally rule out another client, it is more likely that the lethality is due to a HR defect. Note that the impact of *rtt109∆* on HO-induced HR is almost as strong as the one by *rad52∆* (new Figure S2B). Now it reads: “Another hit scored as synthetically lethal was Rtt105 (Figure 2A and Table S2), despite it encodes a chaperone involved in the transfer to the nucleus and deposition at ssDNA of the RPA complex. However, the reduction in the level of RPA at forks in *rtt105∆* cells is slight under normal conditions [37,46], which might explain why Rfa1-MN is inducing DNA damage as inferred from the lethality. Thus, the strong dependency of *RFA1-MN* cells on Rtt105 is unlikely associated with the role of RPA in replication fork stability and checkpoint activation, as suggested by the observation that defects in those processes caused less severe growth defects in *RFA1-MN* cells (see below). This dependency is more likely related to the function of Rtt105 in HR, where it facilitates Rad51 loading at ssDNA for DSB-induced gene conversion and BIR [46]. Actually, the DSB repair defect in *rtt105∆* is almost as strong as that displayed by *rad52∆* (Figure S2B). Remarkably, ssDNA stabilization by RPA is particularly critical for HR mechanisms that involves long-lived ssDNA intermediates, in particular BIR.”

6) The requirement for Dun1 in the absence of Rad53 activation is curious and unexpected from the Mec1-Rad53-Dun1 pathway concept. There are other pieces of evidence for Rad53-independent activation of Dun1 that should be discussed here. These results could help illuminate this non-canonical signaling axis. See for example PMID: 12556502 and discussion therein of other cases.

A more detailed analyses of the checkpoint response requested by the reviewers showed that the *rad53K227A* mutant – catalytically deficient but proficient in dNTP synthesis –affected the growth of Rfa1-MN expressing cells in the presence of CaCl_2_ (new Figure 3C). We have revised the Rad53 western experimental conditions (previous Figure 1D with calcium) and observed that a shift to calcium-containing medium in a wild-type strain causes a transient arrest in S phase that prevents Rad53 activation in the presence of MMS for 2 hours (new Figure S3B). Therefore, we have removed that experiment. Unfortunately, we cannot perform the experiment at longer times because the CaCl_2_ precipitates in liquid medium after 2-3 hours, as explained in the text. From the genetic analysis with the *rad53K227A* mutant, we infer that partial checkpoint activation occurs at high concentrations of calcium, consistent with an accumulation of DSBs and/or BIR-associated ssDNA. Regarding Dun1, we have demonstrated that is required to increase the levels of dNTPs, as the defect of *RFA1-MN dun1* can be rescued by overexpressing RNR and eliminating Sml1 (new Figure 3D). The text has been modified accordingly.

7) The result that a short G1 phase has a negative consequence in RFA1-MN cells may suggest that converging forks play a role, as discussed in the manuscript. Does a slower cell cycle, for example growth on minimal media, rescue this defect, as is observed in bacteria in mutants with replication issues?

We have done the experiment at 22^o^C versus 30^o^C to slow down cell growth, and observed a partial rescue of the defect, according to the expectation (new Figure 4D).

Additional points:

8) I know there is a discussion about this, but I thought it was settled that Sae2 itself is not a nuclease but a cofactor of MRN.

Since this debate is not relevant for our study, we refer to Sae2 just as a factor required to promote the nuclease activity of Mre11, removing “helicase Srs2”. Now it reads: “During DSB-induced HR, Sae2 promotes the nuclease activity of the MRX complex in the initial processing of DSB ends to generate short stretches of 3’-ended ssDNA.”

9) Rad27 is not structure specific but cleaves multiple substrates, see PMID: 10567561 for example. This may affect the discussion of potential mechanisms involved.

“Structure-specific” has been removed in the introduction of Rad27, according to the mentioned paper. In any case, this does not affect the discussion because the two suggested possibilities are related with the known role of Rad27 in the processing of Okazaki fragments and the interaction of Rad27 with the endonuclease Slx4-Mus81 in processing the D-loop, where we do not speculate about the role of Rad27 in this interaction.

10) BIR is only Rad51-independent in one special chromosomal context, and Pol32 cannot mechanistically substitute its role in homology search and strand invasion. The argument at the beginning of the discussion should be eliminated.

We agree that Pol32 cannot substitute Rad51’ homology search and strand exchange activities. It can compensate for the lack of Rad51 in the rescue of DNA lesions generated in a *rad3-102* mutant employed in the referred study, likely stabilizing the D-loop structure (Rad51 by strand exchange and Pol32 by DNA synthesis). In both cases, the annealing activity of Rad52 is essential, likely to invade and initiate HR. In any case, we agree that this situation seems to be specific of this particular allele and the argument has been removed to make Discussion more comprehensible.

11) There is a reference on page 20 to Figure S4B, but there is no such figure. Is Table S2 meant here. Please correct.

It was Table S2 and former S3B; now Table S2 and Figure S4D. Corrected.

12) FigS1A: There is a slight G1-S or S cell cycle delay in the Rfa1-MN construct. Please comment and discuss.

We have observed a slight delay by FACS (see repeat in new Figure 1G). However, budding index and doubling time analyses show similar behaviors in *RFA1-MN* and wild-type cells (new Figure S1C). This, together with the fact that Rfa1-MN expression does not affect cell growth even in the presence of genotoxic agents that affect DNA replication like MMS and HU, prompt us to discard a loss of functionality of the chimera in DNA replication. This has been discussed in the text.

13) FigS2A: RFA1-MN is combined with several factors involved in the chromatin assembly mechanism leading to the conclusion that the pol1-2A2 mutant is the only one that displayed a weak effect on plates with high CaCl2 concentrations, which might be related to a subtle defect in its polymerase activity. It is suggested that the chromatin assembly mechanism does not play a major role in the repair of DSBs at forks. To provide a more comprehensive analysis, the RFA1-MN dpb3∆ strain should be analyzed as well.

Done as suggested; the mutant does not affect *RFA1-MN* cell growth, as the rest of chromatin assembly mutants (new Figure S2C).

14) Fig3A: One of the replicates of the Rfa1-MN mec3∆ strain showed no reduced viability compared to the Rfa1-MN and mec3∆ single mutants, while the other replicate exhibited reduced viability. Please comment on this inconsistency.

This spore is incorrect. Modified with new spores.

15) Please correct typo on page 14: change ‘Chd1’ to ‘Cdh1’.

Corrected.

**Reviewer #2:**

In this manuscript, Amiama-Roig et al. have modified a previously used system in yeast, Chromatin Endogenous Cleavage (ChEC), to induce DNA double-strand breaks (DSB) at replication forks. The authors characterize the genetic requirements of a broken replication fork versus a broken linear DNA molecule. The authors performed a screen to identify several players involved in the repair of DSBs at replication forks. In particular, the authors implicate the requirement of various HR factors, BIR factors, and cell cycle factors in the repair of DSBs at replication forks.

Overall, although the authors have a novel tool to test the genetic requirements of broken forks, the system needs to be further validated, and the candidates identified require further investigation. Data obtained from the screen is over interpreted and provides limited mechanistic insight into the repair of broken replication forks.

Apart from the requested experiments to further validate the system reported below, we have provided additional data supporting:

1) Cleavage at non-replication forks: we show by ChEC that Rfa1-MN can cut DNA in G1 cells (new Figure 1A; previous ChEC is now as Figure S1A and it has been corrected because by mistake the kinetics was reported in seconds instead of minutes). This result is consistent with RPA localization at the transcribed regions of active genes (Sikorski et al. Mol Cell 2011). This is included in the characterization of the system in Results.

2) Supporting preferential cleavage at replications forks: We show that the kinetics of ChEC digestion is faster in S phase cells (new Figure 1A) and, importantly, Rfa1-MN is extremely efficient in digesting replication forks, as determined by 2D/ChEC, where replication intermediates from ChEC-treated cells are analysed by 2-dimensional gel electrophoresis (new Figure 1B; replication intermediates are completely digested in just 45 seconds) (we have performed this approach as ChEC preferentially detects DNA breaks at lineal molecules as the amount of forks relative to the whole genome is low). In addition, we confirmed previous studies by Haber’ group (Kramer et al. MCB 1994) showing that, in contrast to Rfa1-MN-induced DSBs where HR is essential, NHEJ can operate as a backup mechanism at canonical deDSBs (new Figure 1J). These results are consistent with previous studies by ChIP-seq of whole chromosomes and microscopy analyses of RPA foci in G1 and S phases (Li et al. 2018 EMBO J, Li et al. 2022 EMBO J, Wong et al 2019 Mol Cell) that show a preferential accumulation of RPA at replication forks as compared with non-replicated regions in S phase and G1 (ChIP) and of RPA foci in S phase relative to G1 (microscopy). In summary, we conclude from our and previous results: “Although we cannot discard the formation of some DSBs at other regions, the preferential accumulation of RPA at replication forks [37–39], the high efficiency of Rfa1-MN to digest replication forks, and the essential role of HR for *RFA1-MN* cell viability suggest that most of these DSBs stem from Rfa1-MN–cut replication forks, preferentially at the lagging strand that accumulates most ssDNA/RPA (Figure 1K).”

We have included new experiments to respond to some open questions and rephased the different possibilities generated by our genetic analyses, trying to avoid any over-interpretation of the results. In this regard, and following some of the reviewers’ suggestions, we have clarified the roles of DNA resection factors Sae2 and MRX, checkpoint activation, Dun1 and G1 length in the response to broken forks. Please see specific answers to these points.

Finally, we would like to emphasize that we employ a new system where the DSBs are directly generated at the fork, in contrast to previous studies where the fork collapses as a consequence of its encounter with a nick, which is a physiologically different scenario. Our results confirm and extend the genetic requirements obtained in those studies. Actually, our study finds, for the first time to our knowledge, a number of factors that allow to draw a global genetic picture of the cell response to broken forks. In this sense, it involves for the first time (apart from expected ones like HR, checkpoint and cohesin factors), replication-associated HR factors, replisome components and G1-controlling factors that allow to infer new mechanistic pathways. This is a genetic study; we are aware that further molecular analyses of specific mutants are required to propose molecular details, as now indicated in the final sentence of Discussion.

Major comments:

1. Although the ChEC system has been previously used successfully, the Rfa1-MN chimera used in this manuscript needs to be further validated. The authors need to test the Rfa1-MN system in the presence of DSB-inducing drugs like bleomycin, zeocin and following exposure to ionizing radiation.

Done as requested; the *RFA1-MN* mutant displays a wild-type behavior in the presence of high doses of zeocin and ionizing radiation (new Figure S1B). These results support the complete DSB repair functionality of Rfa1-MN previously observed with HO-induced DSBs, in line with its wild-type response to high doses of MMS and HU.

2. The authors need to provide evidence that the Rfa1-MN chimera is functional and makes physiological amounts of protein.

Done as suggested. The Rfa1-MN chimera is expressed as the non-tagged protein (new Figure 1C) and is proficient in DNA damage tolerance (Figure 1D; MMS and HU resistance), DSB repair (Figures 1E and new S1B; zeocin, ionizing radiation and HO-induced DSBs), checkpoint activation (Figure 1F; MMS-induced Rad53 phosphorylation) and DNA replication (new Figure 1G). Only a slight delay from G1 to G2/M was observed by FACS, although the budding index and doubling time were similar in *RFA1-MN* and wild-type cells (new Figure S1C). Importantly, the fact that the *RFA1-MN* mutant behaves as the wild type strain in the presence of high concentrations of MMS and hydroxyurea (HU) indicates that the chimera is also proficient in replication fork processivity and stability even under high replication stress conditions.

3. The authors need to discuss the precise nature of the substrate, and the frequency of DNA lesions formed in the lagging strand using the Rfa1-MN system in response to Ca2+ treatment, which remains unclear.

As previously mentioned, although we cannot discard the formation of some DSBs at other regions, the preferential accumulation of RPA at replication forks [37–39], the high efficiency of Rfa1-MN to digest replication forks, and the essential role of HR for *RFA1-MN* cell viability suggest that most of these DSBs stem from Rfa1-MN–cut replication forks, preferentially at the lagging strand that accumulates most ssDNA/RPA. In addition, we have introduced at the beginning of Discussion putative substrates for Rfa1-MN according to RPA activity, as well as the expected outputs (illustrated in new Figure 5). Now it reads: “HR might be operating at a 1) deDSB generated either between Okazaki fragments (Figure 5a) or 2) by collapse of a converging fork with the gap left at the non-broken strand (Figure 5b), or 3) at a seDSB generated after cleavage of the fork junction, more likely at the lagging strand (Figure 5c).”

Concerning the frequency of broken forks, it is not technically easy to address this point. From the lethality in *rad52* background, we infer that there may be at least one broken fork per cell cycle in the absence of calcium, being this number increased in calcium. We did not detect any increase in HR in cells expressing Rfa1-MN with and without calcium using an unequal sister chromatid exchange system (data not shown). Although in this approach only the forks that break at the repeats of the system can induce HR, this observation suggests that the frequency of broken forks is low. This is a limitation for molecular assays. Nevertheless, as in the case of many other previous (and critical for our understanding of genome integrity) genetic screenings searching for factors involved in the repair of genotoxic conditions (agents like IR, MMS, CPT or HU or mutants affecting genome integrity processes), the frequency of damage is unknown (and the molecular nature of the lesion not completely defined) but it fulfills two important requisites; it is high enough to find hits in the screening but not that high that cells are too sick (note that even at 400mM CaCl_2_, *RFA1-MN* and wild-type cells display a similar growth that is only slightly slower than without CaCl_2_).

Minor comments:

1. Figure 1A: The concentration of Ca2+ has not been mentioned in the figure or the legend.

This Figure has been relocated in panel S1A and the concentration of calcium is indicated in the legend.

2. Figure 1E: The authors note that Rfa1-MN and rad52∆ are synthetic lethal, but Rfa1-MN and rad51∆ are viable in Figure 1H. The authors need to elaborate on the nature of the DNA lesion requiring Rad52.

We realized that this part was confusing and has been restructured and rephrased to make it clear. Not only *rad52∆* but also *rad51∆* (and *rad54∆, rad55∆, mre11∆, rad50∆, xrs2∆* and *rtt105∆*) are lethal in combination with *RFA1-MN*. The difference with *rad52∆* is that in the rest of mutants a few spores could germinate leading to microcolonies that grew better after streaking in a new plate, likely by adaptation to grow with lower level of intracellular calcium or the selection of suppressors. In those mutants, the drop tests were performed with these survivors. To clarify this point, the explanation has been included as soon as mutants *rad51∆* and *mre11∆* are analysed. In addition, all these tests have been relocated in Figure S2A, as the important result is that they are lethal in combination with *RFA1-MN* as determined in the SGA and tetrads analyses. We do not speculate about the differential behaviour with *rad52∆*, but it is likely due to the fact that Rad52 is the only essential HR factor for all recombination mechanisms in yeast, and *rad52* is the yeast mutant with the most severe effects in all HR assays.

3. Figure 1H: Why have the authors not included 400mM Ca2+ data for Rfa1-MN rad51∆. This is an important concentration, since the authors performed SGA analysis at this concentration.

This mutant (a selected survivor, as indicated before) is already dead at 100 and at 200mM. In this first characterization of the system (previous to the screening), we wanted to confirm our hypothesis that mutants previously involved in the rescue of broken forks should display sensitivity when combined with *RFA1-MN*. We selected 400mM because *RFA1-MN* still grew well and detected mutants with low sensitivity (e.g., *RFA1-MN mus81∆*).

4. Figure 1H: The labeling on the most bottom lane is unclear. Is it RFA1-MN, pol32∆ or double?

It is the double mutant; it was included to compare with *rad51*. However, following reviewer 3 suggestion, it has been removed as it compares *pol32∆* with a selected survivor of *rad51∆*.

5. Figure 2B: The authors mention Rfa1-MN rad59∆ cells are synthetic sick. This would imply an annealing step in the repair of broken forks. The authors need to discuss this possibility in their model or discussion.

ssDNA annealing activity is required for all HR events, including BIR. Actually, the essential role for Rad52 in HR is partially due to its annealing activity. If only the mediator activity of Rad52 were essential for HR, *rad52∆* would display similar or even less severe defects than *rad51∆*. Indeed, only the absence of Rad51 and Rad59 generate defects as severe as the absence of Rad52, indicating that the annealing activity of Rad59 can complement for the lack of Rad52’ ssDNA annealing activity, likely in the stabilization of the strand invasion intermediate (reviewed in Prado et al 2003 Current Genetics). Accordingly, now it reads: “The recombination protein Rad59 was also found in the screening but only in the presence of CaCl_2_ (Figure 2B), which is consistent with the essential role of its ssDNA annealing activity specifically in the absence of Rad51 [45].”

6. Figure 2B: The authors need to confirm the effects of Rfa1-MN rtt105∆ on conventional DSB repair in parallel to broken fork repair

We do not fully understand this point. The double mutant *RFA1-MN rtt105∆* is lethal and the one selected in Figure S2A is a survivor that is already sick by the expression of Rfa1-MN. We guess the reviewer means the *rtt105∆* mutant; in agreement with the reported roles of Rtt105 in HR, *rtt105* is deficient in HO-induced DSB repair (see new Figure S2B). As observed for Rfa1-MN-induced broken forks, the defect of *rtt105* in HO-induced DSB repair was as strong as that displayed by *rad52∆*, but in this case none of them is essential as these HO-induced DSBs can also be repaired by NHEJ in the absence of HR (Kramer et al. MCB 1994).

7. Figure 3C: The Dun1 data is overinterpreted. There is no direct evidence provided suggesting an increase in dNTP pool facilitates Rfa1-MN mediated broken fork repair. This interpretation should be moved to the discussion. The authors could also test the strains in the presence of HU and Ca2+ if possible.

We agree that the effect of *dun1* might be associated with some of the roles of Dun1 that are not related to dNTP control. We have performed the requested experiment and observed that the calcium-induced defect is exacerbated in the presence of HU (new Figure S3A), consistent with a deficit of dNTPs as the cause of the *RFA1-MN dun1* growth defect. To confirm this possibility more directly, we have augmented the levels of dNTPs by over-expressing the RNR complex and eliminating the RNR inhibitor Sml1. This background rescued the *RFA1-MN dun1* growth defects (new Figure 3D). Results and Discussion have been modified accordingly.

8. Figure 2H: It is relevant to test the role of yen1∆ and yen1∆ mus81∆ in their assays, since they mention it in the model.

Done as requested (new Figure 1L). Now it reads in the analysis of mutants previously shown to participate in the repair of broken forks: “The sensitivity of *mus81∆* to nick-induced fork breakage is severely aggravated in the absence of Yen1 [17,21], an endonuclease that participates with Mus81 in the resolution of single Holiday junctions (HJs) [41] as those expected from the merging of a BIR-associated migrating D-loop with a converging fork. The absence of Mus81 and Yen1 strongly impaired cell growth in Rfa1-MN-expressing cells, although without being lethal (Figure 1L).” The relevance of this observation is commented in Discussion: “These requirements are consistent with a BIR-like fork restart mechanism, even it is unlikely that extensive synthesis by BIR suffices for cell viability, as inferred from the growth defects of the triple mutant *RFA1-MN mus81∆ yen1∆* (lacking the enzymes required for D-loop-to-fork conversion and HJ resolution after D-loop/fork merging) [17,21]. This growth defect also reinforces the accumulation of seDSBs in *RFA1-MN* cells because Mus81 and Yen1 are not required for SDSA [8].”

9. Figure 4B legend: needs to be elaborated

Done as requested.

10. Discussion:

The authors imply seDSBs formed in the lagging strand at broken replication forks is mutagenic.

This is in stark contrast with recent data that the authors have cited (Pavani et al., Kimble et al., and Elango et al.), that do not require the arrival of a second fork. Please discuss.

A nick at the lagging strand can be bypassed generating a deDSB, as shown in the cited studies. But this bypass depends on the nickase and the structure of the nick, as discusses in Pavani et al (Science 2024) and Elango et al. (Mol Cell 2025). For instance, the nCas9^D^ (employed in those studies) causes a nick that can be bypassed, while the nCas9^H^ leads to the collapse of the fork and a seDSB (the same output is observed when the fork encounter a simple, nickase-independent nick) (Vrtis et al 2021 Mol Cell). Accordingly, BIR can be induced by nicks at both strands (Xu et al. Whitby 2025 Mol Cell). These results indicate that the fork can respond to the nick and/or nickase. To clarify this, now it reads in Introduction: “Actually, the arrival of a converging fork before BIR might explain the detection of double-ended DSB (deDSBs) at some nicks at the leading template [20–22]. A nick at the lagging strand also leads to a seDSB using nicked plasmids in *Xenopus* egg extracts [19], and accordingly, DNA nicks in both leading and lagging strand templates can trigger BIR [22]. However, nickase-induced nicks at the lagging template can be bypassed by the replisome in yeast and mammalian cells leaving a deDSB behind the fork [20–23]. This bypass, though, depends on the nickase and structure of the DNA nick [19,20,22], indicating that the fork can respond to the nick and/or the nickase without collapsing.”

In our system the Rfa1-MN chimera introduces a direct DSB at the fork, which may lead to its collapse. Our finding of mutants supporting HR in the context of replication supports the formation of seDSBs that would be rescued by BIR-like mechanisms.

The statement ‘*this result strongly suggests that converging forks also participate in the rescue of DSBs at forks.*’ needs to be re-worded since there is no direct quantification of the number of licensed origins.

It is well established that a reduction in the length of G1 reduces the number of licensed origins (Ayuda-Durán et al. 2014; Lengronne et al. 2002). Now we show that the strong defect in growth observed in the *RFA1-MN* *GAL1p::SIC1 cdh1∆* mutant even in the absence of supplemental calcium can be partially rescue if the cell cycle is slowed down (new Figure 4D), providing more time for origin licensing. Taking into account that converging forks have been shown to facilitate broken fork repair, we think that a simple explanation for the phenotype of the mutants affecting G1 length is a reduction in the number of converging forks. We have toned down the statement and now it reads in Discussion: “It has been shown that converging forks limit the mutagenicity associated with the repair of a nick-induced DSB, likely by merging with the D-loop [17]. Since a premature entry into S phase reduces the number of licensed origins [102,103], the severe growth defects of Rfa1-MN-expressing cells in combination with a shortening of the G1 phase might be due to a reduction in the number of active forks that could rescue the broken forks.”

The statement ‘*This situation was reverted in HR mutants, which is consistent with the restrictive effect of strand invasion on DNA resection at seDSBs’* needs to be re-worded. It is unclear what HR mutants and phenotypes the author is referring to.

Rephrased; now it reads: “However, *RFA1-MN* cells did not display growth defects and the checkpoint was not required except at high levels of CaCl_2_ (consistent with an accumulation of DSBs and/or BIR-associated ssDNA) or in the absence of Rad52 (consistent with the accumulation of DNA resection-mediated ssDNA at broken forks when strand exchange is abolished [106]).”

**Reviewer #3:**

In this study, the authors exploited a Rfa1-micrococcal nuclease (MN) chimera to induce double-strand breaks (DSBs) in the budding yeast genome. Given that RPA (Rfa1 encodes the largest subunit of the RPA complex) coated single-stranded DNA (ssDNA) is generated during DNA replication, the authors predicted that Rfa1-MN would generate DSBs specifically at replication forks. Cells expressing the Rfa1-MN chimera grow normally but are dependent on the Rad52 protein for viability suggesting that native levels of Ca2+ can activate MN nuclease activity and create lesions during DNA replication that are repaired by homologous recombination (HR). To increase the damage induced by this system, the authors added Ca2+ to the growth media and demonstrated time dependent degradation of bulk genomic DNA. The authors screened a collection of strains involved in the DNA damage response to identify mutants defective for repair of DSBs at replication forks. The group most sensitive to Rfa1-MN expression were core HR genes including the RAD52 epistasis group. Core HR mutants were found to be synthetic lethal when combined with the Rfa1-MN chimera allele, even in the absence of additional Ca2+ to the growth media. The authors also identified several pathways that are important for repair of DSBs at replication forks including the DNA replication checkpoint, the break-induced replication (BIR) pathway, replication coupled nucleosome assembly (RCNA), sister-chromatid cohesion and factors involved in G1 cell cycle regulation that showed growth defects. Mutants defective for these pathways showed Rfa1-MN sensitivity only when Ca2+ was added to the growth media.

While this paper identifies numerous factors involved in repair of Rfa1-MN induced DSBs, questions remain about the specificity of this system to generate DSBs specifically at replication forks. The authors also make several vague or overinterpreted statements that are not well supported by the data presented in the manuscript.

Major comments:

1. While Rfa1-MN is most likely making breaks at the lagging strand of replication forks, the authors should consider the possibility that Rfa1-MN is making breaks in other contexts, such as single-strand gaps behind replication forks, at persistent R-loops, or at ssDNA revealed by the resection machinery. The authors might consider arresting cells in G1 or G2 to ensure that Ca2+-dependent genomic DNA degradation requires passage through S-	phase. At a minimum, the possibility of these non-replication fork breaks should be discussed.

This is an important question that has been addressed with additional information and experiments, and above all, we have tried not to be so categoric as we cannot rule out additional cuts at other regions.

Supporting cleavage at non-replication forks, we show by ChEC that Rfa1-MN can cut DNA in G1 cells (new Figure 1A; previous ChEC is now as Figure S1A and it has been corrected because by mistake the kinetics was reported in seconds instead of minutes). This result is consistent with RPA localization at the transcribed regions of active genes (Sikorski et al. Mol Cell 2011). This is included in the characterization of the system in Results.

Supporting preferential cleavage at replications forks: We show that the kinetics of ChEC digestion is faster in S phase cells (new Figure 1A) and, importantly, Rfa1-MN is extremely efficient in digesting replication forks, as determined by 2D/ChEC, where replication intermediates from ChEC-treated cells are analysed by 2-dimensional gel electrophoresis (new Figure 1B; replication intermediates are completely digested in just 45 seconds) (we have performed this approach as ChEC preferentially detects DNA breaks at lineal molecules as the amount of forks relative to the whole genome is low). In addition, we confirmed previous studies by Haber’ group (Kramer et al. MCB 1994) showing that, in contrast to Rfa1-MN-induced DSBs where HR is essential, NHEJ can operate as a backup mechanism at canonical deDSBs (new Figure 1J). These results are consistent with previous studies by ChIP-seq of whole chromosomes and microscopy analyses of RPA foci in G1 and S phases (Li et al. 2018 EMBO J, Li et al. 2022 EMBO J, Wong et al 2019 Mol Cell) that show a preferential accumulation of RPA at replication forks as compared with non-replicated regions in S phase and G1 (ChIP) and of RPA foci in S phase relative to G1 (microscopy). In summary, we conclude from our and previous results: “Although we cannot discard the formation of some DSBs at other regions, the preferential accumulation of RPA at replication forks [37–39], the high efficiency of Rfa1-MN to digest replication forks, and the essential role of HR for *RFA1-MN* cell viability suggest that most of these DSBs stem from Rfa1-MN–cut replication forks, preferentially at the lagging strand that accumulates most ssDNA/RPA (Figure 1K).” Likewise, we start Discussion by claiming: “In this study, we have generated a chimera of the largest subunit of the RPA complex with the MN that preferentially generates DSBs at replication forks and searched for mutants affected in their repair.” Actually, as now explained in Discussion to introduce the putative substrate for Rfa1-MN (graphically illustrated in new Figure 5), deDSBs can also be generated behind the fork between Okazaki fragments.

It is worth to note that the accumulation of seDSBs (that requires fork breakage) is also supported by the severe growth defect of the *RFA1-MN mus81∆ yen1∆*, because Mus81 and Yen1 are not required for SDSA (the main deDSB repair mechanism in mitotic cells). This argument has also been included in the Discussion.

2. Can the authors provide an estimate for how many breaks Rfa1-MN is generating with/without Ca2+ added to the growth media?

Concerning the frequency of broken forks, it is not technically easy to address this point. From the lethality in *rad52* background, we infer that there may be at least one broken fork per cell cycle in the absence of calcium, being this number increased in calcium. We did not detect any increase in HR in cells expressing Rfa1-MN with and without calcium using an unequal sister chromatid exchange system (data not shown). Although in this approach only the forks that break at the repeats of the system can induce HR, this observation suggests that the frequency of broken forks is low. This is a limitation for molecular assays. Nevertheless, as in the case of many other previous (and critical for our understanding of genome integrity) genetic screenings searching for factors involved in the repair of genotoxic conditions (agents like IR, MMS, CPT or HU or mutants affecting genome integrity processes), the frequency of damage is unknown but it fulfills two requisites; it is high enough to find hits in the screening but not that high that cells are too sick (note that even at 400mM CaCl_2_, *RPA1-MN* and wild-type cells display a similar growth that is only slightly slower than without CaCl_2_).

3. Interestingly, Rad53 is not phosphorylated in cells expressing Rfa1-MN, even after addition of 400 mM Ca2 to the growth medium. Is Rad53 activation observed in the repair-deficient RFA1-MN rad51∆ (or rtt109∆) strain? It seems possible that the ssDNA required for Mec1 activation is being destroyed by Rfa1-MN.

Further experiments have clarified this point. The requested experiment with the *rad53K227A* mutant showed that checkpoint activity (regardless of its function in dNTP levels) is required at high concentrations of calcium (new Figure 3C). We have revised the Rad53 western experimental conditions (previous Figure 1D with calcium) and observed that a shift to calcium-containing medium in a wild-type strain causes a transient arrest in S phase that prevents Rad53 activation in the presence of MMS for 2 hours (new Figures S3B-C). Therefore, we have removed that experiment. Unfortunately, we cannot perform the experiment at longer times because the CaCl_2_ precipitates in liquid medium after 2-3 hours. From the genetic analysis with the *rad53K227A* mutant, we infer that checkpoint activation occur at high concentrations of calcium, consistent with an accumulation of DSBs and/or BIR-associated ssDNA.

We have checked Rad53 phosphorylation in a *RFA1-MN rad50∆* survivor (*RFA1-MN rad59∆* growth is not affected without supplemented calcium) and hardly detected activation (see below). However, this experiment is not conclusive because, as previously mentioned, Rad50 is essential and the *RFA1-MN rad50∆* mutant has been selected from microcolonies where cells have likely been adapted to grow with low intracellular concentrations of calcium or have accumulated partial suppressors. However, we already showed that switching off Rad52 expression in a *RFA1-MN GALp::RAD52* mutant leads to partial Rad53 phosphorylation (now in Figure 3G). The activation is low, likely as a consequence of the basal expression of the *GAL1* promoter that allows *RFA1-MN GALp::RAD52* cells to slowly grow in glucose-containing medium (Figure S1D). These arguments have been included in Results.

4. If Rad53 is responsible for phosphorylating Dun1, it is confusing that the Rad53 mutant would not be sensitive to Rfa1-MN. It is possible that sml1∆ suppresses the rad53∆ phenotype by raising nucleotide pools. The authors should consider the use of a Rad53 kinase dead allele (such as rad53K227A) which does not require the additional deletion of SML1 for cell viability. Similarly, since the authors argue that Dun1 activity raises nucleotide pools for the repair process, they should test sml1∆ for suppression of the RFA1-MN dun1∆ phenotype.

Done as suggested (New Figure 3C). The result demonstrates that checkpoint is partially required at high concentrations of calcium, regardless of its effect on dNTP levels.

The absence Sml1 is not sufficient to rescue the growth defects of *RFA1-MN dun1∆* cells (see below). However, in combination with RNR over-expression, it completely suppressed the growth defect (new Figure 3D), indicating that the effect of *dun1∆* in the rescue of broken forks is due to Dun1’ role in dNTP upregulation in response to DNA damage.

5. The authors show that the fork protection complex (Tof1, Csm3) is required for viability of cells expressing Rfa1-MN. If forks are uncoupled in the absence of FPC, then more ssDNA may be generated at forks providing more substrates for MN cleavage. Thus, the synthetic defect could simply be due to increased damage as opposed to a direct role for these factors in repair. The same argument could be made for the rad27∆ mutation which is known to be synthetic lethal with HR defects, presumably due to a higher burden of S-phase associated DNA damage.

We already discarded this possibility in the characterization of the chimera, where we observed that it grew as the wild type even at MMS and HU concentrations that strongly slow down replication and lead to an accumulation of ssDNA. In results, it read: “Importantly, the fact that the *RFA1-MN* mutant behaves as the wild-type strain in the presence of high concentrations of MMS and hydroxyurea (HU) indicates that the chimera is also proficient in replication fork processivity and stability even under high replication stress conditions.” We have reinforced this critical point in Discussion. Now it reads: “It is important to remark that the growth defects observed in the absence of replication factors are unlikely due to an accumulation of ssDNA and a higher probability of fork breakage by Rfa1-MN or to the additive effects of fork cleavage and replication stress because the *RFA1-MN* mutant behaves as the wild-type strain even in the presence of high concentrations of MMS and HU that strongly impair cell growth.”

6. Figure 4 presents the most novel aspect of this paper by discussing the possibility that a short G1 phase limits origin licensing, which leads to hypersensitivity of replication fork breakage during S phase. However, the authors do not provide concrete mechanistic evidence that the mutants described have an origin licensing defect, or that this defect would in fact complicate repair of breaks at replication forks.

Following a reviewer suggestion, we have demonstrated that slowing down cell cycle at 22^o^C partially rescue the growth defects of the *RFA1-MN GAL1p::SIC1 cdh1∆* mutant at semi-permissive conditions (new Figure 4D). This suggests that the defect is associated with the length of the G1 phase and not with additional functions of these factors (also supported by the observation that growth defects are obtained with different mutants affecting G1 length). The facts that a reduction in G1 phase reduces the number of licensed origins (Ayuda-Durán et al. 2014; Lengronne et al. 2002) and that converging forks seem to help to repair broken forks (Mayle et al 2015) are consistent with our hypothesis that the observed defects are associated with fewer licensed origins. We are aware that future experiments will be required to better understand the molecular role of many of these factors in the repair and rescue of broken forks. We have toned down the statement and now it reads: “It has been shown that converging forks limit the mutagenicity associated with the repair of a nick-induced DSB, likely by merging with the D-loop [17]. Since a premature entry into S phase reduces the number of licensed origins [102,103], the severe growth defects of Rfa1-MN-expressing cells in combination with a shortening of the G1 phase might be due to a reduction in the number of active forks that could rescue the broken forks.”

Minor Points:

1. Many of the spot assays have a high degree of background colonies. Additionally, it would be more visually appealing to display these assays in grayscale/black and white as the many different colors currently displayed is a bit distracting.

They are not background colonies, but CaCl_2_ crystals that sometimes form at high concentrations. These precipitates did not affect the reproducibility of the results. This has now been indicated in the Figure 1 legend. “At high concentration, CaCl_2_ can form crystals that did not affect the reproducibility of the assay.”

Figures are now displayed in grayscale as suggested.

2. The authors note that Rfa1-MN rad52∆ is synthetic lethal and do not perform spot assays with this strain. The same is essentially true for Rfa1-MN combined with pmr1∆, rad51∆, mre11∆, rad50∆, xrs2∆, rad54∆, rad55∆, and rtt105∆. Double mutants isolated from pinprick colonies post dissection likely have suppressor mutations. As such, it does not seem appropriate to include spot assays for these mutants in the manuscript and to compare them with mutants that only show Rfa1-MN sensitivity in the presence of Ca2+ (e.g., pol32∆)

We agree with the argument; nevertheless, we think it is interesting to show that even the selected cells cannot grow in the presence of calcium. For that reason, we have sent these assays to supplementary information (new Figure S2A), except for the comparison between pol32∆ and rad51∆ that has been removed. The text has been appropriately modified.

3. On the last sentence of the first paragraph on page 12, I believe the authors meant to write “Ctf8 and Dcc1” instead of “Ctf18 and Ddc1”.

Corrected.

4. Figure S2B: At what temperature was the sensitivity of smc6-56 mutant to Rfa1-MN tested?

It was tested at 30^o^C. Note that *smc6-56* is defective in the DNA damage response at this temperature (e.g., see Choi et al 2010 MBC; García-Rodriguez et al 2018 NAR or González-Garrido and Prado 2023 Cell R).

5. With the exception of unique sequence specific or genetic contexts, Rad51 is required for BIR in yeast. BIR at generic replication fork breaks as described in this manuscript is Rad51 dependent, as indicated by the synthetic lethality for RFA1-MN rad51, and in other studies showing that Rad51 is required for nick-induced repair. As reported by Mayle et al (2015) for Flp-nick and in this study, Pol32 is not as important as Rad51.

We agree that the compensation effect of Pol32 in the absence of Rad51 is likely specific of the *rad3-102* mutant in the referred study by Aguilera’ group and thereby it has been removed as suggested. Likewise, as mentioned before, the drop assay comparing *rad51∆* and *pol32∆* has also been removed.

6. The authors fail to cite work from the Luke lab showing that mis-regulation of RNaseH2 creates DNA damage during S-phase that requires HR and the RCNA pathway for repair (Schindler et al., 2023). This work should be cited in the Discussion.

Done as requested. Now it reads: “Thus, Rtt109/H3K56ac/ Rtt101^Mms22/Mms1^ might not be specifically required for a DSB at the lagging strand, but for the BIR-mediated restart of a seDSB. Accordingly, this pathway was identified – together with the core HR factors and the MRX complex – among the factors required for the repair of seDSBs generated by rNMP-induced nicks, regardless of their position at the leading or lagging strand [112].”
