# Supplementary material for: A Rfa1-MN–based system reveals new factors involved in the rescue of broken replication forks
Source: PLoS Genet. 2025 Apr 1;21(4):e1011405. doi: 10.1371/journal.pgen.1011405 (PMC11984746; doi:10.1371/journal.pgen.1011405)
Supplement: Attachment — Submitted filename: Amiama.Barrientos.Rebuttal letter.re.docx [file pgen.1011405.s013.docx]

**We are glad that the revised version of the manuscript had adequately responded the reviewer concerns, and strongly appreciate their constructive suggestions to improve the manuscript. An answer to the specific points that still require further clarification can be found below.**

**Reviewers' comments:**

Reviewer's Responses to Questions

Reviewer #1: Amiama-Roig et al.
Revised manuscript

In the revision the authors did a nice job to address my comments with new experimentation, clarifications, and text changes. The consideration of and experimental evidence for non-replication fork targets of Rfa1-MN is a good addition. I do agree that the likely major target will be replication forks and postreplication gaps, as supported by the new experiment shown in Figure 4d showing some suppression at slower growth.

In their model and the discussion, the authors may want to consider the D-loop as an additional target. There is good evidence that RPA binds the displaced strand of the D-loop to stabilize. However, the data show lethality with an HR defect and no HR-dependent lethality, suggesting that D-loop cleavage is not an issue. Maybe this is a point to make in the discussion?

We agree that this possibility should be commented. We have included the arguments and an additional experiment (new Figure S1E) where we show that expression of Rfa1-MN under conditions of high cleavage (200 mM calcium) do not affect the viability of *RFA1-MN* cells in the presence of high levels of HU-induced recombination intermediates (lethal in *rad52∆* cells). Now it reads in Results when arguing about replication forks as the major targets for Rfa1-MN: “We do not consider D-loops as a preferential target for Rfa1-MN-induced cleavage, because Rad52 does not promote, but instead prevents *RFA1-MN* cells lethality. Accordingly, *RFA1-MN* cells displayed a wild-type growth in the presence of high concentrations of HU even in plates enriched with CaCl_2_ to increase the rate of cleavage (Figure S1E).”

We have also mentioned this new Figure in Discussion to reinforce the idea that the defects observed in replication mutants were “unlikely due to an accumulation of ssDNA and a higher probability of fork breakage by Rfa1-MN or to the additive effects of fork cleavage and replication stress because the *RFA1-MN* mutant behaves both with and without calcium as the wild-type strain even in the presence of high concentrations of MMS and HU that strongly impair cell growth (Figures 1D and S1E).” It is important to show this wild-type growth under conditions of high cleavage because some mutants displayed the defect only in those conditions.

Reviewer #2: The authors have adequately addressed my comments, and the revised manuscript is much improved. There are still a few points that could be clarified by revisions to the text:

1. Page 8, line 1: I suggest changing to “..is expressed at the same steady state level as..”

Changed as suggested.

2. Page 10: The rad59 mutation does confer DNA damage sensitivity in RAD51 cells indicating that its activity is not specific to RAD51-deficient cells.

We agree that Rad59 activity is not specific of Rad51 deficient cells, but the defect associated with its absence is strongly aggravated in the absence of Rad51. We have replaced the sentence. Now it reads: “The recombination protein Rad59 was also found in the screening but only in the presence of CaCl_2_ (Figure 2B), which is consistent with the minor effect of *rad59∆* in HR in the presence of Rad51 [45].”

3. The rtt105 mutant has a much less severe phenotype in assays for DSB-induced recombination than canonical HR mutants, such as rad51 (Wang et al., 2021; 2024). Thus, it seems unlikely that loss of the Rad51 mediator function is responsible for the rtt105 RFA1-MN lethality. I wonder if an additional/alternative explanation for the RFA1-MN rtt105 lethality is that there are more MNase-induced breaks in these cells. In the rtt105 background, there would be sub-saturating levels of RPA on ssDNA leaving more ssDNA exposed for MNase cleavage. In the presence of Rtt105, ssDNA would be saturated by RPA shielding the ssDNA from MNase cleavage.

Our HO-sensitivity assay shows a defect in DSB repair almost as strong as that displayed by *rad52∆* (Figure S2B). The role of *rtt105∆* in the mentioned studies is not compared in parallel with other HR mutants, even though the effect in GC and BIR seems to be milder than the one described for *rad51∆* or *rad52∆* cells in other studies. We do not favor a suboptimal amount of Rfa1-MN at ssDNA as the cause of the lethality observed in *rtt105∆*, because as we already mention in the text, the reduction in the level of RPA at forks in *rtt105∆* cells is slight under normal conditions [37,46]. Furthermore, an increase in DNA cleavage does not affect the viability of *RFA1-MN* cells in a wild-type background (cells just grow a bit slower; compare wild type and *RFA1-MN* cells at 0- and 400-mM calcium).

We think that the lethality of *rtt105∆* is mostly due to defective HR, but also aggravated by the role of Rtt105 in checkpoint and replication fork stability, which are also required – to a lesser extent than HR – in the repair of Rfa1-MN-induced broken forks. We have changed the sentence and now it reads: “The synthetic lethality of *RFA1-MN rtt105∆* cells might be related to the function of Rtt105 in HR – where it facilitates Rad51 loading at ssDNA for DSB-induced gene conversion and BIR [46] – and to a lesser extent to the role of RPA in replication fork stability and checkpoint activation (see below).”

4. Page 18: The lack of a requirement for NHEJ to repair damage caused by RFA1-MN does not imply seDSB formation. Recent papers using nCas9 and Flp-nick showed no contribution of NHEJ to repair, even for nicks on the lagging-strand template that give rise to deDSBs. It seems more likely that the ends of replication-coupled deDSBs are not amenable to NHEJ.

The reviewer is right. The sentence “in accordance with the incapacity of NHEJ to operate as a backup mechanism for HR” has been removed.

Since unwinding by CMG would be required to generate ssDNA on the lagging strand template for RPA to bind, it is not clear to me how seDSBs could form using this system. Once CMG has separated the parental strands it should continue translocation on the leading strand template. In vitro studies from the Walter lab showed that CMG can translocate onto the parental duplex at a pre-existing nick on the lagging strand template to make a seDSB but that requires the nick to be ahead of CMG.

In our system, the cleavage can occur directly at the fork junction, which likely disrupts the replisome structure preventing fork progression. This is now indicated in Discussion when referring to the formation of a seDSB: “In the latter case, a direct cleavage at the fork junction would likely disrupt the replisome structure thus preventing fork progression.” This is a major difference with systems where the fork encounters a nick in the template, because in those cases the replisome is not altered and can in principle respond to the nick, as discussed previously. In our system the CMG might also be translocated onto the parental duplex ahead of the broken fork, even if there is no a preexisting nick. It would require elimination of the ssDNA flap generated after the cleavage (see Figure 5c). We prefer not to speculate about this because the nature of these broken intermediates (here and in nick-based systems) is unknown.

Since there could be 100s of DSBs in the presence of Ca ions, the sensitivity of some mutants, such as mus81 yen1, could be due to a fraction of these DSBs being processed by DSBR instead of SDSA.

We agree that a fraction of deDSBs could be processed by DSBR instead of SDSA. However, Mus81 and Yen1 are not the primary mechanism to deal with DSBR-associated dHJs in mitotic cells, but the Sgs1/Top3/Rmi1 dissolvase complex, whose absence does not significatively affect *RFA1-MN* viability even at high concentration of calcium. The resolvase Mus81 and Yen1 would be required only under conditions of massive accumulation of deDSBs to act upon non-dissolved dHJs. Even though it is difficult to determine the number of Rfa1-MN-induced deDSBs, it is highly unlikely that they are in the 100s even at high concentrations of calcium. Since replication forks are the major target of Rfa1-MN, such a high number of deDSB would mean that most if not all forks would break, which would strongly delay cell growth and cause lethality. But even if 100s of deDSBs formed, *RFA1-MN* cells should then be more sensitive to the absence of Sgs1 than to the absence of Mus81 and Yen1. We have clarified in the text the critical point of this argument. Now it reads: “The growth defect of this mutant (*RFA1-MN mus81∆ yen1∆*) also reinforces the accumulation of seDSBs in *RFA1-MN* cells because Mus81 and Yen1 are not required for SDSA and are a backup mechanism for DSBR [8].”

The revised text covering point 4 now reads:

“HR might be operating at a 1) deDSB generated either between Okazaki fragments (Figure 5a) or 2) by collapse of a converging fork with the gap left at the non-broken strand (Figure 5b), or 3) at a seDSB generated after cleavage of the fork junction, more likely at the lagging strand (Figure 5c). In the latter case, a direct cleavage at the fork junction would likely disrupt the replisome structure thus preventing fork progression. The scarce formation of dHJ structures by double strand break repair (DSBR) suggested by the null effect of *sgs1∆* and *smc5-56* mutants could be due to a low accumulation of deDSBs. Alternatively, it might reflect a preferential repair by synthesis-dependent strand annealing (SDSA), as observed for nick-induced deDSBs [22,23]. The accumulation of seDSBs by Rfa1-MN is supported by the finding of BIR-associated factors, fork-associated HR factors, and replisome components for their repair. These requirements are consistent with a BIR-like fork restart mechanism, even it is unlikely that extensive synthesis by BIR suffices for cell viability, as inferred from the growth defects of the triple mutant *RFA1-MN mus81∆ yen1∆* (lacking the enzymes required for D-loop-to-fork conversion and HJ resolution after D-loop/fork merging) [17,21]. The growth defect of this mutant also reinforces the accumulation of seDSBs in *RFA1-MN* cells because Mus81 and Yen1 are not required for SDSA and are a backup mechanism for DSBR [8]. Since the arrival of a converging fork would lead to re-replication if the gap left at the non-broken strand has been repaired, seDSB invasion and D-loop formation could be a mechanism to ensure merging with the converging fork and genetic stability.”
